# The detailed 3D multi-loop aggregate/rosette chromatin architecture and functional dynamic organization of the human and mouse genomes

**DOI:** 10.1186/s13072-016-0089-x

**Published:** 2016-12-24

**Authors:** Tobias A. Knoch, Malte Wachsmuth, Nick Kepper, Michael Lesnussa, Anis Abuseiris, A. M. Ali Imam, Petros Kolovos, Jessica Zuin, Christel E. M. Kockx, Rutger W. W. Brouwer, Harmen J. G. van de Werken, Wilfred F. J. van IJcken, Kerstin S. Wendt, Frank G. Grosveld

**Affiliations:** 1 Biophysical Genomics, Department of Cell Biology and Genetics, Erasmus MC, Wytemaweg 80, 3015 CN Rotterdam, The Netherlands; 2 Cell Biology and Biophysics Unit, European Molecular Biology Laboratory, Meyerhofstr. 1, 69117 Heidelberg, Germany; 3 Cell Biology, Department Cell Biology and Genetics, Erasmus MC, Dr. Molewaterplein 50, 3015 GE Rotterdam, The Netherlands; 4 Genome Organization and Function, BioQuant and German Cancer Research Center, Im Neuenheimer Feld 267, 69120 Heidelberg, Germany; 5 Cohesin in Chromatin Structure and Gene Regulation, Department of Cell Biology and Genetics, Erasmus MC, Dr. Molewaterplein 50, 3015 GE Rotterdam, The Netherlands; 6 Center for Biomics, Department of Cell Biology and Genetics, Erasmus MC, Dr. Molewaterplein 50, 3015 GE Rotterdam, The Netherlands

**Keywords:** Genome organization, Cell nucleus architecture, Nucleosome, Chromatin fibre, Chromatin loops, Chromatin rosettes, Targeted chromatin capture, Polymer physics simulation, DNA sequence organization

## Abstract

**Background:**

The dynamic three-dimensional chromatin architecture of genomes and its co-evolutionary connection to its function—the storage, expression, and replication of genetic information—is still one of the central issues in biology. Here, we describe the much debated 3D architecture of the human and mouse genomes from the nucleosomal to the megabase pair level by a novel approach combining selective high-throughput high-resolution chromosomal interaction capture (*T2C*), polymer simulations, and scaling analysis of the 3D architecture and the DNA sequence.

**Results:**

The genome is compacted into a chromatin quasi-fibre with ~5 ± 1 nucleosomes/11 nm, folded into stable ~30–100 kbp loops forming stable loop aggregates/rosettes connected by similar sized linkers. Minor but significant variations in the architecture are seen between cell types and functional states. The architecture and the DNA sequence show very similar fine-structured multi-scaling behaviour confirming their co-evolution and the above.

**Conclusions:**

This architecture, its dynamics, and accessibility, balance stability and flexibility ensuring genome integrity and variation enabling gene expression/regulation by self-organization of (in)active units already in proximity. Our results agree with the heuristics of the field and allow “architectural sequencing” at a genome mechanics level to understand the inseparable systems genomic properties.

**Electronic supplementary material:**

The online version of this article (doi:10.1186/s13072-016-0089-x) contains supplementary material, which is available to authorized users.

## Background

The structure and function of genomes obviously co-evolved as an inseparable system allowing the physical storage, replication, and expression of genetic information [1–4]. However, the dynamic three-dimensional higher-order architecture of genomes, their spatial and temporal modifications and/or relation to functional multi-dimensional interaction and regulatory networks have yet to be determined in detail (e.g. [4–11]). The DNA double helix and the nucleosome [12–14] have been determined in general structurally at the very highest level of detail including genome sequences and histone modifications. Additionally, it became apparent that genome organization and function indeed form a systems genomic entity ([4, 6, 9, 10, 15–17]; see also references within all these) responsible for gene expression (e.g. [18, 19]) and form the basis for individual differences and disease.

However, the immense size and structural complexity of genomes spanning many orders of magnitude impose huge experimental challenges and hence the higher-order architecture is still widely discussed. How nucleosomes are positioned, spaced, remodelled, and whether and how nucleosome chains fold into fibres at physiological salt concentrations have been matters of continuing debate (e.g. [20]): Finch and Klug [21] proposed a relatively regular solenoid and *in vivo* neutron scattering experiments revealed a compacted fibre with a diameter of 30 ± 5 nm as a dominant nuclear feature [22–25]. In contrast other and especially more recent suggestions range from basically no compaction at all (rev. [26–28]), to highly polymorphic compacted [29, 30] nucleosome position [31] and function-dependent structures [32, 33]. The latter are essential to explain nucleosome concentration distributions [34–37], or chromatin dynamics [38] and functional properties such as the nuclear diffusion of macromolecules [5, 39]. Notably, the fine-structured multi-scaling long-range correlation behaviour of the DNA sequence also predicts a compacted chromatin fibre [5, 16, 40].

The higher-order chromatin architecture has been a matter of even greater debate: Pioneering light microscopy studies by Rabl (1885, [41]) and Boveri (1909, [42]) suggested a hierarchical self-similar, territory like organization. Electron microscopy suggested a more random interphase organization as in the models of Comings (1968, [43, 44]) and Vogel and Schroeder (1974, [45]). In the radial loop scaffold model of Paulson and Laemmli (1980, [46]), ~60 kbp-sized chromatin loops attached to a nuclear matrix/scaffold should explain the condensation degree of metaphase chromosomes. According to Pienta and Coffey (1977, [47]), these loops persisted in interphase and formed stacked rosettes in metaphase. Micro-irradiation studies by Cremer and Cremer (1974, [48, 49]) and fluorescence *in situ* hybridization (FISH) (1988, [4, 50]) and studies thereafter finally confirmed a territorial organization of chromosomes, their arms, and stable subchromosomal domains during interphase, including their structural persistence during metaphase (de-)condensation (see [6, 17, 51]). The assumption since then has been that the ~850 G, Q, R, and C ideogram bands (Additional file 1: Refs. [S1, S2]) split into ~2500 subchromosomal interphase domains. Chromatin rosettes explaining the (sub)territorial folding were visualized by electron microscopy (1989, [52–54]) but remained unappreciated, whereas Belmont and Bruce proposed the EM-based helical hierarchy chromonema fibre (CF) model (1994, [55]). Spatial distance measurements between small FISH-labelled genetic regions led to the Random-Walk/Giant-Loop (RW/GL) model with the first analytical looped polymer description (1995, [56–58]). Here, 1 to 5 Mbp loops are attached to a non-protein backbone, following the line of Pienta and Coffey [47]. Later, a combination of distance measurements by more structure preserving FISH, high-resolution microscopy, and massive parallel polymer simulations of chromosomes and entire cell nuclei, was only compatible with the rosette-like Multi-Loop-Subcompartment (MLS) model. In this model 60 to 120 kbp loops form rosettes connected by a similar sized linker [3, 5, 7, 8, 15, 16, 59]. The MLS model is also in agreement with studies of transcription (e.g. [52, 60]) and replication ([52], and thereafter [61]). *In vivo* FCS measurements of nucleosome concentration distributions and the dynamic and functional properties such as the architectural stability and dynamics of chromosomes [5, 31, 36, 62] or the diffusion of macromolecules [5, 36, 63] are essentially also in agreement with a small loop aggregate/rosette like chromatin folding [5, 35–37, 59, 64]. Fine-structured multi-scaling long-range correlations of the DNA sequence again predict this [5, 16, 40].

However, to further investigate various aspects and to distinguish better between the different architecture proposals crosslinking techniques (used since the last century) were developed into a family of interaction capture techniques (Additional file 2: Table S1) such as 3C [65, 66], 3C-qPCR [67], 4C [68], 3C-seq/4C-seq [69], 5C [70], and Hi-C [71]. They once more confirmed the existence of looping and subchromosomal domains [72], now referred to as topologically associating domains (TADs) with a higher localization accuracy when compared to FISH. These led to a number of suggestions, such as the fractal globule model [71], the loop array architecture in mitotic chromosomes [73], and the highly dynamic loop formation based on single cell ([74]; compatible with a switch and binder model [75]), or cell population experiments [76]. However, these suggestions are based on experimental (raw) data that are open to other interpretations (this publication [5, 11, 37, 62, 64], Imam et al., in prep.) and are in contrast to previous observations (see above). Nevertheless, whatever the suggested architectural model, these methods clearly showed, that physical interactions between functional elements proposed earlier ([77–79]; see review [19]), are at the heart of genome function by regulating gene transcription. These often take place over huge genomic separations by direct contact via a preformed architecture and its modification [7, 8] or the formation of complexes such as in transcription factories [19, 80–82]. Additionally, more structural factors such as CTCF and/or cohesin play a role here ([83] and references therein), which seems obvious also from co-evolutionary considerations.

Here we use *T2C*, a novel selective high-throughput high-resolution chromosomal interaction capture developed by us [84, 85], which detects all probable physical genomic interactions (selective everything with everything) for a specific genomic region. Thus, it provides the means for efficient and cost effective “architectural genome sequencing” and allows to approach the major open questions discussed above with high quality: (i) Whether a chromatin fibre exists and how it is compacted, (ii) how it is folded, (iii) whether there is a general scaling behaviour of this architecture in agreement with the fine-structured multi-scaling long-range correlations of the DNA sequence organization, (iv) whether this satisfies also the functional requirements with respect to the genomic life-cycle as well as dynamic *in vivo* properties, and (v) whether all this is consistent with earlier experiments from a few to the megabase pair level. First we briefly describe the *T2C* design used here to investigate the human chromosome 11p 15.5–15.4 IGF/H19 locus, the mouse chromosome 7qE3–F1 β-globin region, as well as 15 regions under different differentiation and functional aspects basically from the base pair to the entire chromosome level. Next we show that *T2C* reaches the fundamental resolution limits where “genomic” statistical mechanics and uncertainty principles apply which is of fundamental importance for architectural *T2C* result interpretation. Thereafter, we show the high interaction frequency range, the reproducible detection of rare interaction events, and the high signal-to-noise ratio >10^5^–10^6^—all at the statistical limit. Next we further analyse these loci in terms of the 3D architecture which suggests that a chromatin quasi-fibre with ~5 ± 1 nucleosomes/11 nm forms stable ~30–100 kbp loops clustered into stable aggregate/rosette like subchromosomal domains connected by a similar sized linker, with only minor but significant variations in the architecture in terms of cell types/functional states. In depth combination with super-computer polymer simulations as well as scaling analysis of the 3D architecture and the DNA sequence itself (where this architecture is represented by sequence specific “footprints”) results in the same conclusion and confirms the tight co-evolutionary entanglement between genome architecture and sequence. This is in excellent agreement with recent *in vivo* FCS measurements of the dynamics of the chromatin quasi-fibre and a developed analytical polymer model [11]. Consequently, *T2C*, polymer simulations, DNA sequence organization, *in vivo* dynamic FCS measurements, and an analytical model are all in agreement. Since this is also consistent with the heuristics of the field, we finally conclude this architecture, its dynamics, and accessibility balance stability and flexibility ensuring genome integrity and variation enabling gene expression/regulation by self-organization of (in)active units already in proximity.

## Results

### *T2C* a novel selective high-resolution high-throughput chromosome interaction capture

**Fig. 1:**
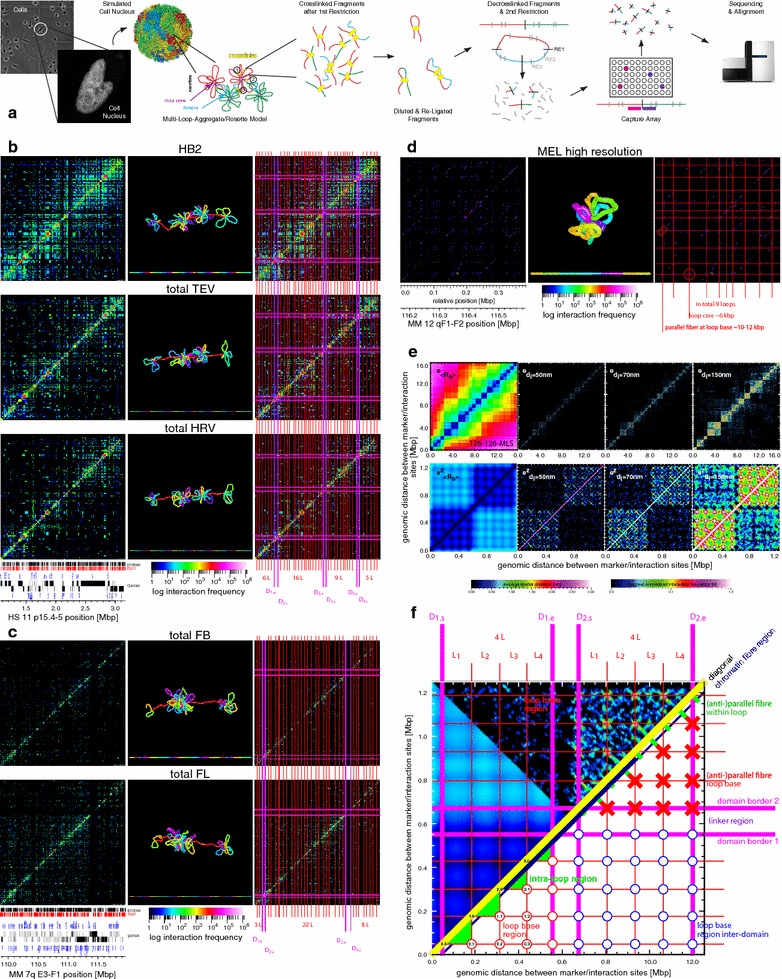
*T2C* description, interaction mapping, and direct determination of the chromatin quasi-fibre and the aggregated loop/rosette 3D architecture of the human and mouse genomes: **a** Cell nuclei in a population of cells (transmission light and fluorescence microscopy, [89]) have an underlying chromatin architecture (simulated cell nucleus containing 1.2 million polymer segments; resolution 5.2 kbp, i.e. ~50 nucleosomes; Multi-Loop-Subcompartment (MLS) rosette model with 126 kbp loops and linkers; [5]). After crosslinking the DNA is restricted within the nucleus by a 1st restriction enzyme, before the crosslinked fragments are extracted and diluted such that intra-fragment re-ligation is favoured. After de-crosslinking, the re-ligated material is shortened by a 2nd restriction enzyme or sonication and purified by a capture array with oligos designed next to the 1st restriction enzyme, before paired-end-sequencing over the ligation position. After alignment to the reference genome, this results in interactions frequency matrices (b–d) and scaling curves (Fig. 2). **b**, **c** Interaction matrices (logarithmic and colour-coded scale; *left* and *right*) of the human IGF/H19 11p 15.5-15.4 region (**b**) in HB2, HEK293T TEV (intact cohesin) and HEK293T HRV (cleaved cohesin) as well as the mouse β-globin 7qE3-F1 region (**c**) for fetal brain (inactive β-globin) and liver cells (active β-globin) show the formation of subchromosomal domains separated by a linker (borders: *pink lines, right*; D1s, D1e: start and end of domains), which consist of loops (*red lines*; 8L: number of loops), representing due to the grid-like pattern loop aggregates/rosettes. A grid-like pattern is also visible in the interactions between the domains and corresponds to the interactions of loops and loop bases of interacting domains. Near the diagonal the aggregation into a chromatin quasi-fibre and loop internal structures are visible (zooming in and out the images can make this clearer). Between different cell types and functional states only some local differences are visible resulting in a consensus architecture and allowing simulation of the 3D architecture (middle; resolution <~1 kbp). Note that the simulation is driven by the dominant consensus architecture. **d** The interaction matrix of a 380 kbp subchromosomal domain in the mouse 12qF1–F2 region at high resolution clearly shows the regular rosette-like picture with a detailed structure of the loop base with in- and outgoing loop fibre stretches as seen in simulations (**e**, **f**). **e** Simulated Multi-Loop-Subcompartment (MLS) model with an averaged spatial distance map for exact spatial distances 〈*R*_*S*_〉 (*left*) and on the diagonal normalized interaction maps for interaction radii 〈*d*_*i*_〉 of 50 nm, 70 nm, and 150 nm (*right*), for an MLS model with 126 kbp loops and linkers [16 Mbp upper and 1.2 Mbp zoom-in (*z*) lower row), showing clearly the formation of domains connected by a linker, their interaction, and the underlying loop aggregates/rosette architecture, with (anti-)parallel fibre stretches at the loop base. The dependence on the interaction radii corresponding to different crosslink probabilities is also clearly visible. **f** Sketch of the different structures visible on different scales in the experimental and simulated interaction matrices (spatial distance matrix: *left*; simulated interaction matrix: *upper*) (from **e**): On the smallest scale, near the diagonal the compaction of nucleosomes into the quasi-fibre (*yellow line*) and the fibre regime (*dark blue line*) can be found. On the largest scale the domains are clearly bordered (*pink lines*) and connected by a linker. On medium scales the loop aggregate/rosette like structure is characterized by the loop bases (*red circles*: within domains, *blue circles*: between domains) as well as the loop interactions (*green triangles*). The fine structure of the loops representing the (anti-)parallel loop stretches at the base (*red crosses*) and within loops (*green stretches near diagonal*)

*T2C* is a selective high-resolution high-throughput chromosome interaction capture approach [84, 85] which we developed to design interaction capture studies with respect to their purpose—here efficient, high resolution/quality, and cost effective “architectural genome sequencing”. Briefly, *T2C* in this setup involves (Fig. 1a, details in Additional file 1: Supplemental Methods): (i) Starting with ~10^7^ cultured/prepared cells, (ii) the cells are formaldehyde-fixed (i.e. all kinds of combinations of nucleic and protein crosslinks are formed), (iii) permeabilized to allow intra-nuclear cutting with a 1st restriction enzyme, (iv) extensively diluted to promote mono-molecular re-ligation reactions, before (v) de-crosslinking, purification, and final shortening of the DNA chimeric fragments to sizes <500 bp by a 2nd high-frequency restricting enzyme or by sonication. Then, (vi) a region-specific DNA library of interacting fragments is produced using hybridization to region specific arrays of DNA oligonucleotides, representing the end of each restriction fragment produced by the 1st restriction enzyme. With ~10^9^ molecules of each hybridization-optimized oligonucleotide the capture is always in the linear range well below saturation relative to e.g. ~10^7^ input cells. (vii) After elution, the hybridized fragments are paired-end sequenced, and (viii) each sequence pair is trimmed up to the 1st restriction enzyme and mapped to the whole reference genome. Only uniquely mapped sequences are used (eventually only between the two restriction enzymes). No other correction or cleaning resulting in information loss is performed due to the very nature of this method (see below).

Thus, *T2C* has clearly several advantages with respect to studying genome architecture in depth: (i) It provides a choice between costs, resolution, interaction frequency range, size of the captured region, and multiplexing of samples in a study-specific manner. E.g. a ~500 bp average fragment resolution, in a 2 Mbp region, with six orders of magnitude interaction frequency range, and multiplexing of ten samples can be easily achieved sequencing 5 lanes. (ii) The design of the oligonucleotide position ensures optimized data cleanness and high signal-to-noise ratio, allowing maximum interaction information with a minimum amount of sequencing (Fig. 1b–d; Additional file 2: Table S1, Additional file 3: Table S2, Additional file 4: Table S3; Additional file 5: Figure S1, Additional file 6: Figure S2, Additional file 7: Figure S3). (iii) Additionally, the process has been optimized for structure, and thus architectural preservation [5, 59], minimal DNA loss during the procedure, and no use of signal amplification until sequencing when a limited number of PCR cycles could be performed (Additional file 1: Supplemental Methods, Additional file 2: Table S1).

To investigate the chromatin fibre conformation and the 3D genome architecture at the required resolution we chose the human chromosome 11p 15.5–15.4 IGF/H19 locus and the mouse chromosome 7qE3–F1 β-globin region (Additional file 3: Table S2). Both ~2.1 Mbp regions have been well studied by FISH and other capture techniques. Bgl II or Hind III as 1st and Nla III as 2nd restriction enzyme yields average fragment sizes of 3–6 kbp with many fragments, however, near the principle limit of the technique of a few base pairs (Additional file 5: Figure S1, Additional file 6: Figure S2, Additional file 7: Figure S3, Additional file 8: Figure S4, Additional file 9: Figure S5, Additional file 10: Figure S6; average nucleosomal repeat length ~195 bp; 3–6 kbp correspond to ~15–30 nucleosomes). To determine the general chromatin fibre conformation at still higher resolution and to gain further insights into small scale architectural features, we also investigated 15 other regions (Additional file 3: Table S2) covering in total 99.5 Mbp distributed over 10 different mouse chromosomes using Apo I as 1st restriction and sonication instead of a 2nd restriction leading to average fragment length of 549 bp (with many much smaller). This is even more at the technical limit and at nucleosomal/molecular resolution (Additional file 5: Figure S1, Additional file 6: Figure S2, Additional file 7: Figure S3, Additional file 8: Figure S4). To investigate architectural and functional differences between species, cell lines, functional, and architectural differences, the human breast endothelial 1–7HB2 cell line (HB2), the HEK293T TEV/HRV RAD21-eGFP cell lines allowing cleavage of cohesin [83], and mouse fetal brain and fetal liver [β-globin (in)active] cells were used. To investigate the chromatin fibre conformation at high resolution undifferentiated murine erythroleukemia (MEL) cells were used.

### *T2C* reaches the fundamental resolution limits where “genomic” statistical mechanics and uncertainty principles apply

Since for “architectural sequencing” resolution is key, designing *T2C* using short fragment lengths down to even a few base pairs applying frequently cleaving restriction enzymes (Additional file 3: Table S2; Fig. 1b–d; Additional file 4: Table S3, Additional file 5: Figure S1, Additional file 6: Figure S2, Additional file 7: Figure S3, Additional file 8: Figure S4) not only molecular resolution (mind e.g. also the persistence length of free DNA ~50 nm, i.e. ~140 bp; typical protein/nucleosome binding regions are ~100–500 bp) is reached and thus the fundamental limits of crosslinking techniques, but also the mechanism of observation is now, however, on the same scale as the observables (in *analogy* to classic and quantum mechanics). Actually due to the stochastics following the bias of the system behaviour, the observables, the observation, and thus the measured values are constrained by what we call “genomic” statistical mechanics with corresponding uncertainty principles. This originates from the individual complexity of each highly resolved interaction with a unique but coupled individual probabilistic fragment setting in each cell at a given time, e.g.: (i) The cell population has a distribution of cell states and functional differences, (ii) each fragment has a more or less dynamic individual DNA, RNA, protein, restriction association and length, and hence (iii) a different crosslinking, restriction, re-ligation, oligonucleotide capture, sequencing, and mapping efficiency. The actual conditions and components can be determined only partially with high accuracy while with low accuracy otherwise and are eventually even entirely destroyed by the measurement. In essence, the entire *T2C* measurement process is highly quantitative but the local origin of this (including biases e.g. due to the oligonucleotide sequence or position), and thus its comparability, remains elusive due to its local individuality and our present incapability to determine all parameters linked in a complex network in detail and simultaneously as well as the attached biased system noise. Thus, the central limit theorem applies with an overlap of system inherent and real noise stochastics, and hence in the end only probabilistic analyses and statements can be drawn as hitherto is well known from classic mechanics, and more so from quantum (mesoscopic) systems. Consequently, population based or multiple single-cell experiments have to be interpreted and understood in a “genome” statistical mechanics manner with uncertainty principles due to the inseparability of factors/parameters also seen there. Thus, in practical terms, valid results are obtained when the statistical limit is reached, i.e. when scaling up the experiment does not narrow down the distribution any further and does not lead to fundamental (overall) changes anymore in observables. Due to the complexity involved, this has the immediate consequence that there are currently no means for adequate corrections. Even if certain biases might be known, the effect of a correction in terms of the many *T2C* steps remains illusive. This is the case for any interaction capture technique, although the effects of the individual complexity are partly averaged out by the lower resolutions mostly used in previous studies. This is no longer the case at the fundamental resolution limits. Nevertheless, if the statistical limit is reached and if the quality parameters like resolution, frequency range, and signal-to-noise ratio are sound, conclusions could be drawn as in the many cases of classic mechanics, and more so of quantum (mesoscopic) systems within the discussed boundaries.

### *T2C* reproducibly detects rare genomic interactions at the statistical limit with unprecedented signal-to-noise ratio

For the above mentioned experimental systems, with ~10^7^ input cells, the corresponding samples (e.g. two different states) were multiplexed on the capture array to guarantee identical conditions (Additional file 4: Table S3). Only sequences unique in the entire genome with a reasonably small mismatch rate (accounting for sequencing differences to and errors in the reference genome; see Additional file 1: Supplemental Methods) and cleaned for sequences only mapping between the 1st and 2nd restriction sites were analysed. Approximately ~60–380 million paired-end sequencing reads were produced of which ~10–65 % could be mapped uniquely (Additional file 4: Table S3). The regional interactions (after normalization for the total counts within the region) sorted and plotted in an upright squared interaction matrix/map with a logarithmic and rainbow colour-coded frequency range [86], including the diagonal (non- or self-ligation), show directly the quality of the experiments and the unprecedented frequency range spanning 5–6 orders of magnitude (Fig. 1b–d; Additional file 5: Figure S1). Thus, also rare interactions with a frequency of 10^−4^–10^−6^ can be found and visualized under these conditions of region size, fragment resolution, and sequencing depth. We estimate an overall/cumulative (i.e. from cells to interaction matrix) efficiency of *T2C* of ~0.1–1.0 % from the ratio of cumulated counts per fragment to the number of input cells of ~10^7^. The interaction patterns show, that the level of the stable statistical mechanical limit is reached, since data from different sequencing lanes or experiments (whether multiplexed or not) only show visually minor statistical deviations (Fig. 1b–c; Additional file 5: Figure S1). Quantitatively, the statistical measures we used (e.g. frequency distributions) also hardly show a change upon e.g. a twofold increase of input cells or sequencing. At the statistical level reached, such a change leads only to an increase in novel interactions <0.1 %, mostly in the lowest interaction frequency regime. In contrast, a tenfold sequencing decrease results in a massive interaction loss of >25 %. Most importantly, all the interaction matrices of different experiments are reproducibly mostly empty. Only ~5–15 and 1.0–1.5 % of the possible interactions show a signal for the IGF/H19 and for the β-globin, respectively (Additional file 4: Table S3). Thus, there is no obvious uniform noise/background, despite the high number of sequence reads and the high number of diagonal elements showing entries of non- or self-ligated fragments. The “emptiness” is also not arbitrary, but structured, and appears virtually the same in replicates, different cell types or functional states (Fig. 1b–d; Additional file 5: Figure S1, Additional file 6: Figure S2, Additional file 7: Figure S3). Moreover, interactions neither suddenly appear statistically nor cluster statistically somewhere near other or more prominent interactions. The signal-to-noise ratio is >10^5^–10^6^, even though noise could in principle appear at any step of the procedure, and even when assuming a highly unlikely biased distortion of a normal distributed noise signal towards e.g. interactions. A shot-noise (e.g. Poisson-like) analysis confirms this, in agreement with the change being <0.1 % during experimental scale-up (see above). Consequently, these values show that an analysis of these data with respect to genome architecture can be conducted within the limits of the above mentioned genome mechanical statistics constraints.

### The chromatin quasi-fibre forms stable loops clustered into aggregate/rosette like subchromosomal domains connected by a linker

The interaction patterns (Fig. 1b–d; Additional file 5: Figure S1, Additional file 6: Figure S2, Additional file 7: Figure S3) can also be recognized clearly on all scales (within and between domains), including their re-emergence as attenuated repetition on other scales since genomes are scale-bridging systems [5, 15]. This behaviour shows once more the sensitivity of *T2C* allowing 3D architecture investigations despite the numerous and nonlinear parameters involved, since the probability that such repetitive patterns arise stochastically and even reproducibly is negligibly small relative to the number of those potentially formed combinatorially by hundreds of fragments. Additionally, *T2C* reveals agreement with other interaction techniques, e.g. 4C-seq, but with much cleaner and sharper interaction patterns for the same fragment setting (Additional file 6: Figure S2, Additional file 7: Figure S3). The interaction patterns are next interpreted on the scales associated with the chromatin fibre, subchromosomal domains, and within the subchromosomal domains.

(i)  On the smallest genomic scale (Fig. 1b, c; Additional file 5: Figure S1, Additional file 6: Figure S2, Additional file 7: Figure S3), a dense and high interaction frequency pattern is observed in the region from 3 to 10 kbp (i.e. <~5–15, and ~50 nucleosomes, respectively; for quantification, see scaling analysis below) along each point of the diagonal. This pattern varies independently of the local fragment size with distinct interactions and non-interacting “gaps” in-between. This is different from a homogenous random-walk or Rayleigh-like interaction “smear” decreasing uniformly and monotonously with increasing genomic separation. Additionally, the extension of the band of interactions is also smaller than that a random-walk of nucleosomes would predict. A structurally uniform fibre like that seen in the (solenoid-like) helical chromatin fibre model [21] would result in a highly regular and defined pattern, which is also not observed. Thus, the pattern suggests, that there are defined stable interactions at the scale of DNA/nucleosomes forming an irregular yet locally defined and compacted structure. Hence, nucleosomes must form an irregular fibre, which we refer to as a “quasi-fibre” due to its inherent variation with average properties (e.g. an average linear mass density). While reading along the diagonal local interactions, compaction of nucleosomes, as well as other local properties of the chromatin quasi-fibre can be determined. In contrast to a basically uncompacted sea of nucleosome like organization [26–28], the formation of such a quasi-fibre is in agreement with previous experimental results [21, 22], as well as simulations [32, 33]. This is also consistent with a variety of compacted structures described throughout the literature (see e.g. [29, 30, 32, 33]), the absolute nucleosome concentration distributions [35, 36], the dynamic and functional properties such as the architectural stability and movement of chromosomes [3, 5, 39, 62, 64], chromatin dynamics [38], as well as the diffusion of molecules inside nuclei (e.g. [5, 39, 64]). Moreover, recent genome-wide *in vivo* FCS measurements of the chromatin quasi-fibre dynamics [11] also suggest such a chromatin quasi-fibre with variable, function-dependent properties. (See below for a quantification of *T2C* for the quasi-fibre properties.)
(ii)  On the largest scale, stable square-like domains (TADs; [72]) are visible in the range of several hundred kbp to ~1–1.5 Mbp with clear borders and interactions with other domains (Fig. 1b–d; Additional file 5: Figure S1). They are more prominent e.g. in the IGF/H19 region, which shows two complete and two incomplete domains (Fig. 1b), when compared to the β-globin region with its single full domain and only two partially visible domains at the borders of the captured region (Fig. 1c). The domains feature several general properties: Firstly, the interaction frequency within domains has in general a higher average uniform height compared to interactions between domains, with a sharp drop at the edge of domains. The exact position of the border can be deducted from the folding within the domain and can therefore be respectively assigned exactly (see below). Thus, there is a cascade-like (average) behaviour of interactions with increasing genomic separation as predicted before [3, 5, 15, 59], in contrast to the often expected general monotonous interaction decrease with growing genomic separation. Moreover, the interactions to other domains are clearly defined also in detail. Secondly, between the borders of the domains there is a clear transition or linker region, which again can be determined with respect to the folding of the chromatin quasi-fibre within the domain (see below). In and around these linker regions especially strong and complicated interactions are present depending on the specific domains. Such interactions originate from a combination of the chromatin quasi-fibre possibly not being shielded as is the case within the domains as well as the folding of the chromatin quasi-fibre itself (see below). A closer inspection of the interactions in the vicinity of the linker actually allows several interpretations in terms of the underlying domain architecture folding giving rise to these patterns. We favour that the genetic regions of the domains next to the linker interact more frequently compared to other domain parts due to the breaking of spatial isotropy. Two other possibilities that this is due to allelic differences (i.e. the patterns arise from two different allelic domain architectures), or that the linker being a very small linker domain consisting e.g. of a single or a few loops, are much less likely (see below). A closer inspection of interactions near the linker in combination with the dynamic behaviour of subchromosomal domains (see dynamics below and Additional file 11: Movie S1, Additional file 12: Movie S2, Additional file 13: Movie S3, Additional file 14: Movie S4) points also to a directionality along the “back-bone” (the combination of several linkers of several subchromosomal domains), which is breaking the spatial isotropy of single unconnected subchromosomal domains. Consequently, these results confirm the existence of structurally stable subchromosomal domains which by (de-)condensation or (de-)looping explain the (de-)condensation of chromosomes through the cell cycle [4, 5, 17, 47, 51–54, 59–62]. The interaction pattern between subchromosomal domains and at their borders points already to a loop aggregate/rosette like architecture, since neither a free random-walk, an encaged random-walk, a random or a fractal globule like folding, nor a Random-Walk/Giant-Loop architecture would lead to sharp and defined borders. Instead, they would lead to gradual/soft transitions instead. Constantly changing and thus very dynamic architectures with an average topology of these models or even that of a highly dynamic loop aggregate/rosette like architecture would also not result in the observed patterns. This is in agreement with previous predictions on subchromosomal domains [4, 5, 7, 8, 16, 17, 51–54, 59, 62]. Moreover, these patterns are also in agreement with *in vivo* FCS measurements of the nucleosome concentration distribution [35, 36], the dynamic and functional properties such as the architectural stability and movement of chromosomes [5, 39, 62], chromatin dynamics [39], as well as the diffusion of molecules inside nuclei (e.g. [5, 39, 64]). Moreover, recent genome-wide *in vivo* FCS measurements of the dynamics of the chromatin quasi-fibre come to the same conclusion with characteristic functional differences [11]. The intrinsic chromatin fibre dynamics with movements on the millisecond scale (Additional file 11: Movie S1, Additional file 12: Movie S2, Additional file 13: Movie S3, Additional file 14: Movie S4) also points to the fact that the subchromosomal domains must have a stable architecture since otherwise they would dissolve immediately (see simulations below [11]). The break of the spatial isotropy of sequentially adjacent subchromosomal domains visible in the linker region is also linked to this stability.
(iii) At intermediate scales within the subchromosomal domains, the interaction pattern is characterized by clearly distinct gaps and a crossed linear (grid-like) arrangement of interactions (Fig. 1b–d; Additional file 5: Figure S1, Additional file 6: Figure S2, Additional file 7: Figure S3). Interestingly, the linear pattern continues outside the subchromosomal domain and “crosses” with the linear pattern originating from the sequentially subsequent domain. Furthermore, the pattern outside is much simpler/clearer since it lacks the extra interactions originating inside the domain from e.g. the chromatin quasi-fibre, or its higher-order structure like e.g. intra-loop or loop-loop interactions (for illustration see Fig. 1e, f). This grid of interactions can also be quantified by projecting the interactions vertically and horizontally over the entire matrix, resulting in a peak-like pattern along the chromosome sequence (Additional file 15: Figure S7; see also [11], for details). These peaks coincide with the grid-like pattern (Additional file 15: Figure S7). Projections within or outside the domains lead in essence to the same patterns with nevertheless subtle characteristic differences (see also [11]). Since interactions on scales of tens of kilo base pairs can only be due to chromatin looping, the conclusion must be that several consecutive loops have a coinciding loop base and hence form a loop aggregate/rosette like architecture. Hence, the interactions between subchromosomal domains result from the interactions of (i) loops from domains next to each other, (ii) loop bases of subsequent loop aggregates/rosettes when there is a relatively low density of loops, and (iii) mitotic chromosomes present in the cell population. The borders of the domains seen on the medium scale (see above) are determined by the loops, and thus also the linker between subchromosomal domains is given by the end and start of loops of two subsequent subchromosomal domains. The border behaviour of domains near the linker was already discussed (see above). Determination of the loop positions and sizes (Additional file 16: Table S4, Additional file 17: Table S5) visually as well as by projections (Additional file 15: Figure S7; for further details see also [11]) with an error on the level of corresponding local fragment resolution and with respect to the loop base structure of ~3 kbp, reveals a consensus architecture independent of cell type or functional state with loop sizes of 48.6 ± 14.5 ± 2.4 kbp (average, StDev, StErr) and linker sizes of 46.7 ± 15.1 ± 8.7 kbp in the mouse β-globin region. In the human IGF/H19 locus the values are 57.8 ± 16.2 ± 2.9 kbp and 69.2 ± 19.2 ± 13.6, respectively. The subchromosomal domain sizes can now be calculated in detail for those subchromosomal domains which are completely covered by the *T2C* array: excluding the linker, the size is 1343.6 ± 3 kbp for the single complete subchromosomal domain in the β-globin region, as well as 728.5 ± 3 and 403.4 ± 3 kbp for the two complete subchromosomal domains of the IGF/H19 locus.

Although the Apo I *T2C* experiment was designed to elucidate the details of the chromatin fibre conformation only, one finds e.g. a 380 kbp subchromosomal domain region showing this pattern in greater detail (Fig. 1d). In addition to showing the same stable loop aggregate/rosette like architecture with 37.0 ± 9.9 ± 3.3 kbp loops (Additional file 18: Table S6), and a subchromosomal domain size of 333.3 ± 3 kbp, part of the detailed loop base fine structure with in- and outgoing loop fibres spanning a region of ~6 kbp can be seen (see simulations below; Fig. 1f; Additional file 19: Figure S8, Additional file 20: Figure S9).

The observation that the linear grid-like pattern outside of the domains is also not a homogeneous smear, shows that the loops and their arrangements into loop aggregates/rosettes are stable and not very variable. Once more the gaps between interactions as well as the grid-like pattern inside and outside the domains show that a free random-walk, an encaged random-walk, a fractal globule like folding, nor a Random-Walk/Giant-Loop architecture would lead to the patterns we find. Constantly changing and thus very dynamic architectures with an average topology of these or even that of a highly dynamic loop aggregate/rosette like architecture would also not result in these patterns. Finally, a non-compacted chromatin quasi-fibre, which a sea of nucleosome like organization predicts [26–28], would result in hugely homogeneous and very dynamic interaction possibilities, and thus patterns we do not find. Of course, the relatively simple notion of a quasi-fibre forming loop aggregates/rosettes connected by a linker becomes more complex due to the variation along the quasi-fibre, the variation of loop size and structure (e.g. super-helical topologies), and their arrangement either at the loop base or core of the loop aggregates/rosettes. Consequently, also on this architecture level the aggregate/rosette architecture also links interphase with metaphase very nicely and shows the architectural persistence during (de-)condensation within the replication process in agreement with experimental data (see [51] and thereafter). Moreover, this agrees with previous predictions on the internal structure of subchromosomal domains [4, 5, 7, 8, 16, 17, 51–54, 59, 61] and again is also in agreement with *in vivo* FCS measurements of the nucleosome concentration distribution [35, 36] and the dynamic and functional properties such as the architectural stability and movement of chromosomes [5, 39, 62], chromatin dynamics [39], as well as the diffusion of molecules inside nuclei (e.g. [5, 39, 64]). Most importantly, the analysis of recent *in vivo* FCS measurements [11] shows similar loop sizes and loop numbers per subchromosomal domain. Thus, both *T2C* and the FCS *in vivo* measurements are in excellent agreement even though we investigate a number of specific regions with *T2C*, opposed to averaging over several regions in the FCS *in vivo* measurements, which suggests that this architecture occurs genome wide. We would like to stress again that the intrinsic chromatin fibre dynamics (on the millisecond scale) point to stable subchromosomal domains since the structure would otherwise dissolve immediately (see also simulations below; Additional file 11: Movie S1, Additional file 12: Movie S2, Additional file 13: Movie S3, Additional file 14: Movie S4; and [11]).

### Comparison with the consensus 3D genome architecture shows small differences between species, cell type, or functional state

To investigate how the genome architecture depends on species, cell type, functional or structural differences due to regulation or deliberate system distortion, we investigated the human IGF/H19 11p 15.5–15.4 region in human HB2, HEK293T TEV (intact cohesin), and HEK293T HRV (proteolytically cleaved cohesin) cells [83], and the mouse β-Globin 7qE3-F1 locus in mouse fetal brain (FB; inactive β-globin) and fetal liver (FL; active β-globin) cells: As has been seen before (see introduction for any a 3C-type assay) the subchromosomal domains are clearly very similar under different conditions (Fig. 1b, c; Additional file 5: Figure S1). The denser interaction pattern found in the HB2 cells when compared to the HEK293T cells may be due to differences in the level of crosslinkability. Comparing mouse FB to FL cells only shows subtle differences often belonging to single or a small group of interactions resulting from activation of the β-globin locus (Fig. 1c; Additional file 5: Figure S1, Additional file 7: Figure S3). Cleaving cohesin, which is thought to play a major constitutive role in genome architecture, does not lead to dramatic changes on all scales despite some clear interaction losses and gains. Visual or quantitative determination of the loop positions also shows only minor differences (Additional file 15: Figure S7), which nevertheless might be functionally important. This might suggest that once formed, cohesin may not be required anymore to maintain the overall subchromosomal domain architecture. Thus, the detailed role of cohesin (as well as other factors like CTCF) in interphase chromatin remains unclear and needs to be clarified.

Consequently, these and other experiments from various laboratories as already mentioned show that organisms rely on a consensus architecture (overview in [4, 17]). This architecture has small functional variations on all scales from the chromatin quasi-fibre to the subchromosomal domains within the genomic regions. Between the subchromosomal domains, the architecture obviously varies more than within domains in agreement with the FCS *in vivo* measurements [11], where differences were found for different genomic regions or functional states such as eu- and hetero chromatin, or during massive changes by (de-)compacting the chromatin quasi-fibre by Trichostatin A or Azide treatment. The dynamics of the chromatin quasi-fibre on the millisecond scale in comparison with the size of the differences stresses again how stable this architecture is (see also simulations below; Additional file 11: Movie S1, Additional file 12: Movie S2, Additional file 13: Movie S3 and Additional file 14: Movie S4; and [11]). Hence, this illustrates the notion of the variation of a theme and points to the evolutionary balance between flexibility and stability of genome architecture in agreement with other findings/predictions [4–10, 15–17, 51–54, 59–61]. The biological implications of this are discussed below.

### Simulated polymer models *in silico* predict and confirm the genome organization in detail found by *T2C*

To better understand the above results we developed polymer models with preset conditions (i.e. without attempting to fit data; [3, 5, 7, 8, 15, 59, 87, 88])—briefly (see Additional file 21: Supplemental Results; Additional file 22: Table S7): We simulated the Random-Walk/Giant-Loop and the Multi-Loop Subcompartment (Additional file 23: Figure S10) including their dynamics and stability with sufficient information/aspects of free random-walks, random, or fractal globules. The two-dimensional spatial distance and interaction maps (Fig. 1e, f; Additional file 19: Figure S8, Additional file 20: Figure S9) calculated from this not only reflect the underlying models even in subtle details (such as the (anti-)parallel neighbouring of the chromatin quasi-fibre at loop bases; Fig. 1d–f) but also show that only an MLS and thus loop aggregate/rosette like genome architecture could explain all the above observations and thus confirm previous predictions (see introduction; [4, 5, 7–10, 15, 17, 47, 51–54, 59, 61, 87, 88]). The simulations also show large emptiness of interaction matrices and its link to the existence of a dedicated chromatin quasi-fibre as well as the appearance of non-equilibrium effects hinting on the behaviour of domain borders near the linker (see above). The stability of the architecture can also be illustrated by e.g. the decondensation from a mitotic chromosome into interphase (Additional file 11: Movie S1): Any 3D architecture would dissolve within seconds if it would not be stable which agrees with the analytical polymer models developed recently to describe both structure and dynamics of the chromatin quasi-fibre [11]. Moreover, using this simulation approach we also visualized the 3D organization and its dynamics using the experimental interaction matrices as input. Since *in vivo* chromosomes are adiabatic systems (they never fold from scratch), we used here the consensus loop and domain positions (Additional file 16: Table S4, Additional file 17: Table S5, Additional file 18: Table S6) as input starting conditions, rather than dropping a free linear polymer chain into the interaction landscape expecting it to fold in a defined knot-free 3D architecture. The outcome (Fig. 1b–d, middle) confirms that the chromatin quasi-fibre forms rosette-like subchromosomal domains with a high degree of agreement with the experiments and the analytical model mentioned above [11].

### Simulations and experimental *T2C* show a fine-structured multi-scaling behaviour revealing general aspects and the detailed aggregate/rosette 3D genome organization/architecture

**Fig. 2:**
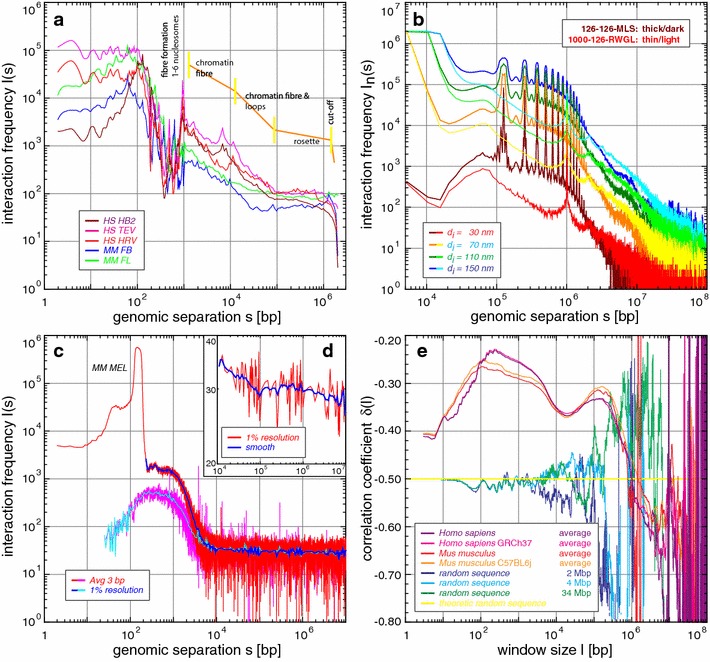
Scaling analysis of experiments, simulations, and the DNA sequence showing the formation of a chromatin quasi-fibre and the loop aggregate/rosette genome architecture: **a** The fine-structured multi-scaling resulting from the *T2C* interaction frequency as a function of the genomic separation for the human IGF/H19 11p 15.5–15.4 region and the mouse β-globin locus 7qE3–F1 (3 bp average (1–200 bp) and thereafter a grouping with a 1 % resolution per order of magnitude which for clarity is smoothed by a running window average for >10^3^ bp; see also Additional file 27: Figure S14; the values <10 bp are due to the algorithm used and for transparency not discarded since they nevertheless show the extrapolation from values >10 bp), shows: (i) The structure of the nucleosome, (ii) the formation of a plateau from 195 to ~1000 bp, indicating the formation of a chromatin quasi-fibre with a density of 5 ± 1 nucleosomes per 11 nm, (iii) the chromatin quasi-fibre regime, (iv) a mixed chromatin fibre/loop regime with a slightly higher interaction decrease, (v) the plateau indicating the loop aggregate/rosette state, and (vi) in principle the linker regime (not visible in **a** but in **d**). **c**, **d** The fine-structured multi-scaling is even clearer for the average of 15 loci covering in total ~99 Mbp in mouse MEL cells with subnucleosomal fragment resolution: After an initial increase a plateau is reached from ~50 to ~100 bp, followed by a sharp peak from ~110 to 195 bp (width at plateau level ~85 bp), followed by a second ~10 % decreasing plateau up to 1.0–1.2 kbp, which after a sharp decent until ~10^4^ bp transits to the known multi-scaling behaviour (**d**, compare with **a**). With this resolution the fine structure visible (Additional file 28: Figure S15), can be associated with the binding of the DNA double helix to the nucleosome, since up to ~195 bp many of the small peaks (the most prominent at 145 bp) can be associated with the fine structure in the fine-structured multi-scaling behaviour of DNA sequence correlations (**e**; Additional file 28: Figure S15, Additional file 29: Figure S16). Whereas the structure of the nucleosome vanishes using “secured” interactions (**c**, *pink* and *light blue*), above 195 bp the plateau and multi-scaling behaviour remain. Again the values <10 bp are due to the algorithm used and for transparency not discarded since they nevertheless show the extrapolation from values >10 bp. **b** The interaction scaling of a simulated Multi-Loop-Subcompartment model with 126 kbp loops and linkers as well as a Random-Walk/Giant-Loop model with 1 Mbp loops and 126 kbp linkers consistently shows for different interaction radii a multi-scaling behaviour. The MLS model shows the characteristic rosette plateau, followed by the random scaling regime of the linker conducting a random-walk. The peaked fine structure originates from the loops forming the rosettes. In contrast, the RWGL model is characterized by random-walk regime and only one major fine structure attributable to the single loops. At greater scales the limit of the entire chromosome is seen in the cut-off. The MLS model agrees in detail with experiments (**a**, **c**–**d**) and the DNA sequence organization (**e**). **e** The fine-structured multi-scaling long-range correlation behaviour of each of two human and mouse strains shows clearly again the architectural features: a general increase until a plateaued maximum (including the 145 bp peak), a first plateau area until ~1200 bp, transition to a sharper decrease at ~3.6 kbp (the sweet point used in the calculation of the persistence length) until a minimum ~10–20 kbp and a second statistically significant maximum at ~100 kbp, followed by a random regime and a final cut-off. The first maximum and plateau are characteristic for the nucleosome and formation of the quasi-fibre (**c**; Additional file 28: Figure S15, Additional file 29: Figure S16) which then transits to chromatin loops and their clustering into loop aggregates/rosettes which are connected by a random-walk behaving linker. Thus, due to the higher statistics here, the architectural features and their tight representation within the DNA sequence organization are even clearer

To comprehensively investigate and quantify the general behaviour of interactions as a function of genomic separation in a unified scale-bridging manner, we already used scaling analysis to understand genome organization and showed its capabilities (see Additional file 1: Supplemental Methods; Additional file 24: Figure S11; [5, 16, 59]). Again the scaling of the interaction frequency for the different simulated models (see Additional file 1: Supplemental Methods, Additional file 21: Supplemental Results; Fig. 2b; Additional file 25: Figure S12, Additional file 26: Figure S13) represents all model parameters in detail (which holds for other scaling measures, Additional file 24: Figure S11) and predicts again that chromosomes show clear long-range power-law scaling, with a multi-scaling behaviour and a fine structure on top in excellent agreement with the alternative analytical model [11]. Determination of the experimental scaling behaviour (see Additional file 1: Supplemental Methods, Additional file 21: Supplemental Results) of the IGF/H19 locus, the β-globin region (Fig. 2a; Additional file 27: Figure S14), and that of the average of 15 regions in MEL cells (Fig. 2c, d; Additional file 28: Figure S15), which has a higher (nucleosomal) resolution, for scales >10^4^ bp, all interactions clearly show fine-structured multi-scaling long-range power-law behaviour (Fig. 2a; Additional file 27: Figure S14), the details of which are only in agreement with the multi-loop aggregate/rosette like architecture (Fig. 2b; Additional file 25: Figure S12, Additional file 26: Figure S13) as predicted by us [5, 16, 59]. In agreement with the simulations this represents (i) the general interaction decrease of the chromatin quasi-fibre up to ~3 × 10^4^–10^5^ bp, (ii) the stable loop and aggregated loop/rosette like structure in the subchromosomal domains from ~3 × 10^4^ up to 10^5^–10^6^ bp, (iii) the subchromosomal domain like structure from ~10^5^ to 10^6^ bp, and (iv) the random-walk behaviour of the subchromosomal domain linkers above ~0.8 × 10^6^ bp (i.e. the “backbone” behaviour of the entire chromosome). As before the differences between species, cell type, or functional states are again small, and the behaviour again shows the stability and functional variability of the system. We also found this scaling behaviour for Hi–C experiments of others (e.g. [71, 73, 74, 76]), suggesting the same 3D architecture (Imam et al., in prep.).

### High-resolution *T2C* scaling analysis reveals the detailed nucleosome structure and proves the formation of a chromatin quasi-fibre

Interestingly, we also get a dedicated fine-structured multi-scaling behaviour on scales from the base pair level up to 10^4^ bp [5, 15, 16]. This is especially true for the combined scaling curves of the 15 Apo I restricted regions due to the high resolution of a few base pairs and the high statistical validity (Fig. 2c, d; Additional file 27: Figure S14, Additional file 28: Figure S15). The dedicated fine structure (Additional file 28: Figure S15) suggests clearly that this general multi-scaling behaviour up to ~195 bp (Additional file 28: Figure S15C) is associated with the nucleosome (Additional file 21: Supplemental Results; [14]) and with the polymer behaviour of the nucleosomal chain thereafter—all features we found earlier by DNA sequence pattern analysis (see below; [5, 15, 16]). We also find multiples of the 145.5 bp and the 195 bp nucleosomal repeat length, e.g. at 290 bp as well as at 385 bp the peaks are exactly where di-nucleosomal features are expected (Fig. 2c; Additional file 28: Figure S15B). From a detailed analysis (Additional file 21: Supplemental Results) we conclude that nucleosomes N4–N6 see the first nucleosome N1 with nearly the exact same probability, but the interaction decreases dramatically for N7 and thereafter. Thus, each individual nucleosome has on average 4–6 clearly distinct nearest neighbour nucleosomes, suggesting the formation of a chromatin quasi-fibre with an average (!) density of 5 ± 1 nucleosomes per 11 nm (see Additional file 21: Supplemental Results for the detailed calculation). Moreover, the genome-wide *in vivo* FCS measurements of the dynamics of the chromatin quasi-fibre [11] show similar average quasi-fibre densities.

### Apparent and average persistence length *L*_p_ of the chromatin quasi-fibre

To gain insight into the average mechanical properties of the chromatin quasi-fibre, we calculated the average apparent persistence length *L*_p_ from the interaction scaling behaviour between 10^3^ and 10^4^ bp—briefly (for details Additional file 21: Supplemental Results): At the so called sweet point at ~3.6 kbp (Fig. 2e; see below) where the nucleosome composition transitions to an average fibre for 4–6 nucleosomes per 11 nm, *L*_p_ ranges from ~80 to 120 nm, respectively. This is in agreement with earlier values (see introduction; [32, 33]), with values derivable from spatial distance measurements between genetic markers [5, 7, 8, 87], and again with values for *L*_p_ extractable from genome-wide *in vivo* FCS measurements [11]. Importantly, this average stiffness predicts that the average loop sizes will have to be on the scale seen above to ensure e.g. their stability, strongly supporting the experimental findings.

### The DNA sequence organization shows fine-structured multi-scaling long-range correlations tightly entangled with the 3D architecture

Since what is near in physical space should also be near (i.e. in terms of similarity) in DNA sequence space and this presumably genome wide [5, 15, 16, 40], and because evolutionary surviving mutations of all sorts will be biased by the genome architecture itself and vice versa, we also investigated the correlation behaviour of the DNA sequence (see Additional file 1: Supplemental Methods; [5, 16, 40]; and references therein) for two different human and mouse strains (Fig. 1e; Additional file 29: Figure S16, Additional file 30: Figure S17, Additional file 31: Figure S18, Additional file 32: Figure S19, Additional file 33: Figure S20, Additional file 34: Figure S21)—briefly (see Additional file 21: Supplemental Results): Again we found species-specific multi-scaling behaviour long-range power-law correlations with a fine structure representing the (i) the nucleosome, (ii) the compaction into a quasi-fibre, (iii) the chromatin fibre regime, (iv) the formation of loops, (v) subchromosomal domains, and (vi) their connection by a linker. On all scales this is equivalent for the different scaling measures used (Fig. 2b; Additional file 21: Supplemental Results, Additional file 24: Figure S11, Additional file 25: Figure S12, Additional file 26: Figure S13). Moreover, the transition from the basic nucleosomal compaction into the quasi-fibre regime (“sweet” point) can be easily seen at ~3.6 kbp. Additionally, on the fine-structural level, the already previously proven association to nucleosomal binding [5, 16, 40] is not only found again (Additional file 29: Figure S16), but also is in agreement with the fine structure found in the interaction scaling (Additional file 28: Figure S15). Also the loop aggregated/rosette structure is present, predicting loop sizes from ~30 to 100 kbp and subchromosomal domain sizes from ~300 kbp to ~1.3 Mbp (see also [5, 16, 40]). All this does not only hint that, in contrast to the regional *T2C* data, the genome folding is a genome-wide phenomenon, but additionally that this architecture is stable and persistent, since sequence reshuffling or other destructive measures would result in a loss of this pattern. This would also be the case for an unstable architecture, which would not leave a defined footprint within the sequence. Once more this agrees with our simulations of the dynamics as well as the genome-wide *in vivo* FCS measurements [11]. Moreover, thus the 3D architecture and DNA sequence organization are indeed co-evolutionary tightly entangled (review of previous notions in [5, 16]). Consequently, in the future from the DNA sequence and other higher-order codes (e.g. the epigenetic code) most architectural genome features can be determined, since also most structural/architectural features and vice versa left a footprint on the DNA sequence and other code levels as one would expect from a stable scale-bridging systems genomic entity.

## Discussion and conclusions

Here we present the much debated 3D genome architecture and its entanglement with the DNA sequence from a few to the megabase pair level of the eukaryotic human and mouse genomes based on combining a novel selective high-throughput high-resolution chromosomal interaction capture (*T2C*), with a scaling analysis of the architecture as well as the DNA sequence organization, and polymer simulations. *T2C* has many a significant advantage, ranging from cost effectiveness, via a huge signal-to-noise ratio, to reaching the level of the “genomic” statistical mechanics with uncertainty principles. The latter is of major importance since here fundamental limits are reached with consequences for the setup and interpretation of experiments involving the architecture and dynamics of genomes. Actually, we face a situation very similar to the revolution in quantum mechanics brought about at the beginning of the twentieth century. Thus, an entirely new way of thinking will be needed to further determine and understand the organization and function of genomes.

With this background, we show here (i) the association of the DNA to the structure of the nucleosome core in detail and the existence of a chromatin quasi-fibre with an average of 5 ± 1 nucleosomes per 11 nm with an average persistence length *L*_p_ from ~80 to 120 nm, (ii) the existence of stable chromatin loop aggregates/rosettes connected by a linker with loops and linkers ranging from ~30 to 100 kbp (with details of the fibre folding at loop bases), (iii) the existence of a consensus architecture with only small differences between species, cell type, or functional states likely to persist through the cell cycle, (iv) the existence of fine-structured multi-scaling behaviour of the architecture, and last but not least that (v) the genome architecture is closely linked to the fine-structured multi-scaling long-range behaviour of the DNA sequence. This is a consistent scale-bridging systems picture of the 3D architecture, its dynamics, and functional variation of two mammalian genomes from the single base pair to the megabase pair level. All this is in agreement with many observations about the architecture, its dynamics, the diffusion of molecules, as well as the replication, storage, and expression of genetic information which have been made in the field (see “Background”). Most interestingly, this is in agreement with novel genome wide *in vivo* FCS measurements of the chromatin quasi-fibre dynamics [11]. Inevitably, there are still many an open question, such as the identification of all the molecule complexes (proteins, RNA, etc.) involved in looping, their dynamics, the inherent variability in the system, but our results provide now a framework for “architectural and dynamic sequencing” and the detailed analysis after all major architectural components in the human and mouse genome have been determined.

The implications of the architecture presented here are many-fold, of which we would like to mention a few: (i) The balance between stability and flexibility of the whole system ensures that the overall genome integrity is maintained when local disturbance/damage takes place due to its modular build, while at the same time it allows fine adjustment of the architecture to enable the development of different gene expression programs/cell types. (ii) The signals due to functional interactions do not stand out above those due to proximity, which is an intrinsic property of the loop aggregate/rosette like folding of the genome. This suggests that the interaction of functional elements (both with respect to transcription as well as to replication) is achieved between fragments that are already in close proximity before their function is required. This proximity and being “tethered” in a subchromosomal domain increase the probability of interaction. (iii) This architecture is open enough to allow the rapid diffusion of molecules such as transcription factors and also allows the movement of sequences to self-organize and form active and inactive units of the genome. These (and other) aspects together form an inseparable system giving rise to a functional genome.

## Additional files

**Additional file 1: Supplemental Methods.**

**Additional file 2: Table S1.** Comparison between different chromosome interaction capture methods, showing their different application potential with respect to scientific aims and their signal-to-noise ratio which could function as an intrinsic quality statement (O: one; M: many; A: all; O<->O: one-to-one; P: primer; PCR: polymerase chain reaction; RE: restriction enzyme; Sel.PCR: selection with PCR; Seq: sequencing).

**Additional file 3: Table S2.** The quality and multiplexability of *T2C* is shown by a detailed overview of the regions investigated (grouped) on one capture array, of the Homo sapiens (HS) and Mus musculus (MM) genomes, with their chromosome and chromosomal position and size, the use of which 1st and 2nd restriction enzyme or in case of very high-resolution sonication, as well as the average fragment size calculated from $\left\langle {L_{Fragment} } \right\rangle = L_{Region} /N_{Fragment}$, and the number of oligos per region. The “name” of the region gives the borders with respect to the ideogram bands.

**Additional file 4: Table S3.** Sequencing and interaction statistics of the experiments done with *T2C* for the regions investigated (grouped) for the Homo sapiens (HS) and Mus musculus (MM) genomes, with respect to the number of capture arrays used, whether and how the multiplexing was done, results in sequenced reads of which a sub-fraction could be uniquely mapped, and finally sorted into square interaction matrices (notably, the matrix is mirrored at the diagonal), which can be analysed in total or according to whether the interactions are within the matrix or on the matrix diagonal concerning the number of existent interactions, their fraction of in total possible interactions, and the frequency distribution of the frequency of the interactions. For the high-resolved regions using Apo I as restriction enzyme and sonication as 2nd “restriction” due to the low number of sequence reads with respect to the total number of interactions no analysis concerning the interactions within the region was performed.

**Additional file 5: Figure S1.** Reproducibility, variation, and statistical limit in *T2C*: Whereas in the case of the human IGF/H19 11p 15.5–15.4 region in HB2 cell (A; HB2 1.1) only one experiment in one sequencing lane was done, in the HEK293T TEV (B) and HEK293T HRV (C) cell system one capture array was used and two lanes were sequenced (TEV 2.1 & 2.2; HRV 2.1 & 2.2). In the case of the mouse β-globin region 7qE3-F1 for fetal brain (D) and fetal liver (E) cells, one capture array was used and two lanes were sequenced (FB 2.1 & 2.2; FL 2.1 & 2.2) with an additional single capture array and one sequenced lane for fetal liver cells (E; FL 1.1). The matrices clearly show the reproducibility in different sequence lanes (B-E) as well as between different arrays (E) and that in different cell types the basic structural organization is the same (A-C; D-E). The variation between different lanes and arrays is extremely small and shows, that summing up the different lanes only leads to minor additional interactions (compare totals to individuals), thus, in principle the statistical limit is reached. Note: The images have a logarithmic frequency range with rainbow-coloured visualization and the matrices were normalized for comparability.

**Additional file 6: Figure S2.** The interaction network of the IGF/H19 region HS 11p 15.5–15.4 resulting from *T2C* shows not only its high quality but also the agreement and differences between different capture techniques: Obviously, *T2C* leads to much more detailed and clearer results (B, C, E) compared to HiC for IMR90 cells (A; data from [72]), and shows interactions of loops at the domain borders or possibly hint at two domain borders (B), suggesting that either i) neighbouring loops interact at domain borders, i.e. there are two larger interaction domains whose borders interact with the entire other interaction domain, ii) there are two sets of interaction domains with a different border either due to the two alleles always present or any other subpopulation of cells, or iii) there is one large interaction domain followed by a very small interaction domain, which is followed again by a larger interaction domain. On the fragment level *T2C* shows not only a clearer dedicated pattern of complex interaction networks (B), but also detailed visualization of *T2C* interactions from one viewpoint (linear frequency range: C; logarithmic frequency range: E) shows these interaction networks not only in much more detail and clearer compared to 4C-seq (D, F), but also identifies more novel, i.e. previously unknown interactions. Also immediately the advantage of a logarithmic frequency range with a rainbow-coloured visualization becomes clear as well as the fact that a squared matrix representation is much easier to understand in terms of relating interactions either with structural or annotational features due to the perception of the human visual cortex trained to horizontal and vertical analysis.

**Additional file 7: Figure S3.** *T2C* interaction network of the β-globin region MM 7qE3-F1: Again *T2C* leads to interaction matrices with high-resolution, a high-frequency range, and unseen quality (fetal brain: A; fetal liver: B; again the matrices are normalized to each other for comparability), with all the known interactions, as e.g. between the β-globin promoter and the local control region (LCR) and between the LCR-3’HS1 sites, and the increased interaction degree of the active β-globin gene. These *T2C* data can be further annotated by other data (bottom), e.g. restriction enzyme sites, transcription factor binding sites, histone modifications, and other data, where again the high resolution and quality of *T2C* will allow for the first time to make sound statements. And again immediately the advantage of a logarithmic frequency range with a rainbow-coloured visualization becomes clear as well as the fact that a squared matrix representation is much easier to understand in terms of relating interactions either with structural or annotational features due to the perception of the human visual cortex trained to horizontal and vertical analysis.

**Additional file 8: Figure S4.** The normalized frequency distribution of the fragment sizes for Bgl II, Hind III, and Apo I, shows the high resolution with many fragments being at the limit of what can be captured, i.e. ~50 bp. Thus, the resolution reached for many fragments even with relatively infrequent restriction enzymes as Bgl II and Hind III, is near or at the fundamental limits of crosslink techniques (persistence length of free DNA on average ~50 nm or ~140 bp; typical protein/nucleosome binding region ~200–500 bp). Additionally, the normalized frequency distributions within the region are a very good representation of the general restriction distribution of the enzymes, with minor local variances. In the case of Apo I, due to the many regions and their size, i.e. the high degree of representation (~1/30 of the entire genome) no difference was found here, so that only the Apo I frequency of the regions is shown.

**Additional file 9: Figure S5.** The high-quality optimization of *T2C* in the 1st restriction, ligation, and 2nd restriction of the human HB2, HEK293T TEV, and HEK293T HRV samples: A, Agarose gel (0.6% wt/vol) showing the primary enzyme restriction by Bgl II (six-cutter enzyme) for the H2B samples, which typically produces a smear of DNA fragments between 0.4–12 kbp (two replicates, HB2-1 and HB2-2 are shown). B, Agarose gel (1.5% wt/vol) showing that after ligation for the H2B samples, the DNA smear has returned to a sharp band around 12 kbp (two replicates, HB2-1 and HB2-2 are shown). Ligated samples were loaded undiluted and diluted 1:10. C, Agarose gel (1.5% wt/vol) showing the secondary enzyme restriction by Nla III (four-cutter enzyme) for the H2B samples, which results in a DNA smear of 0.1–2 kbp (the first replicate HB2-1 was used for the array). D, Agarose gel (0.6% wt/vol) showing the primary enzyme restriction by Bgl II (six-cutter enzyme) for the TEV and HRV samples, which typically produces a smear of DNA fragments between 0.4–12 kbp. E, Agarose gel (1.5% wt/vol) showing that after ligation for the TEV and HRV samples, the DNA smear has returned to a sharp band around 12 kbp. Ligated samples were loaded undiluted. F, Agarose gel (1.5% wt/vol) showing the secondary enzyme restriction by Nla III (four-cutter enzyme) for the TEV and HRV samples, which results in a DNA smear of 0.1–2 kbp.

**Additional file 10: Figure S6.**The high-quality optimization of *T2C* in the 1st restriction, ligation, and 2nd restriction of the mouse fetal liver (FL) and fetal brain (FB) using Hind III as 1st and Nla III as 2nd restriction enzyme, as well as fetal liver using Apo I as 1st restriction enzyme and sonication: A, Agarose gel (0.6% wt/vol) showing the primary enzyme restriction by Hind III (six-cutter enzyme) for the fetal liver and brain samples, which typically produces a smear of DNA fragments between 0.4–12 kbp (two replicates are shown). B, Agarose gel (1.5% wt/vol) showing that after ligation for the fetal liver and brain samples, the DNA smear has returned to a sharp band around 12 kbp for different amounts of DNA. C, Agarose gel (1.5% wt/vol) showing the secondary enzyme digestion by Nla III (four-cutter enzyme) for the fetal liver and brain samples, which results in a DNA smear of 0.1–2 kbp. D, Agarose gel (0.6% wt/vol) showing the primary enzyme digestion by Apo I (five-cutter enzyme) for a fetal liver sample, which typically produces a smear of DNA fragments between 0.2–5 kbp. E, Agarose gel (1.5% wt/vol) showing that after ligation for a fetal liver sample, that the DNA smear has returned to a sharp band around 12 kbp. F, Agarose gel (1.5% wt/vol) showing the sonication efficiency of the ligated material for a fetal liver sample for different amounts of DNA (1–4 µl of DNA).

**Additional file 11: Movie S1.** Brownian Dynamics simulated decondensation from a metaphase starting configuration of a simulated Multi-Loop-Subcompartment model with 126 kbp loops and linkers with segment length of 50 nm (~5.2 kb). The whole movie is 750 ms long and shows how abruptly the metaphase chromosome expands explosively due to its high density while opening the linker which is constrained to a loop in metaphase. Nevertheless, the rosettes form distinct chromatin territories in which the loops do not intermingle freely (see also Figure S10) in contrast to other models such as the RW/GL model. The final shape and form in a whole nucleus would be determined by the limitations the other adjacent chromosomes provide. The difference densities during decondensation also resemble nicely the conditions of shorter linkers, general genome regions with higher densities, or also the variation of nuclear volumes. Notably, the intrinsic movement of the chromatin fibre is clearly taking place on the millisecond scale, and hence, obviously a topological preformed architecture would dissolve within seconds if it would not be stable.

**Additional file 12: Movie S2.** Brownian Dynamics simulation of the consensus architecture of the IGF/H19 region at HS 11p 15.5–15.4, with a segment length of 20 nm (~2.0 kbp; colours of loops like in Fig. 1b middle, with additional linkers at the beginning and end of the region in red). The whole movie encompasses 146 ms and shows the high intrinsic dynamics of the loops and the loop aggregates/rosettes. Obviously, the single subchromosomal domains are constrained by the subsequent subchromosomal domains as for the β-globin locus (Movie S3) and in contrast to the Igh locus (Movie S4), which are connected directly by the linker. Hence, obviously a topological preformed architecture would dissolve within seconds if it would not be stable. Nevertheless, the loop aggregates/rosettes form distinct subchromosomal domains in which the loops do not intermingle freely (see also Figure S10) in contrast to other models such as the RW/GL model. The final shape and form in a whole nucleus would be determined by the limitations adjacent chromosomes provide.

**Additional file 13: Movie S3.** Brownian Dynamics simulation of the consensus architecture of the β-globin locus at MM 7q E3-F1, with a segment length of 20 nm (~2.0 kbp; colours of loops like in Fig. 1c middle, with additional linkers at the beginning and end of the region in red). The whole movie encompasses 146 ms and shows the high intrinsic dynamics of the loops and the loop aggregates/rosettes. Obviously, the single subchromosomal domains are constrained by the subsequent subchromosomal domains, which are connected directly by the linker. Hence, obviously a topological preformed architecture would dissolve within seconds if it would not be stable. Besides, the constrains resulting from the relatively large loop number of the relatively big subchromosomal domain are also obvious compared to the IGF/H19 region (Movie S2) or the single subchromosomal domain of the Igh locus. Nevertheless, the loop aggregates/rosettes form distinct subchromosomal domains in which the loops do not intermingle freely (see also Figure S10) in contrast to other models such as the RW/GL model. The final shape and form in a whole nucleus would be determined by the limitations adjacent chromosomes provide.

**Additional file 14: Movie S4.** Brownian Dynamics simulation of the consensus architecture of the Igh locus at MM 12q F1-F2, with a segment length of 20 nm (~2.0 kbp; colours of loops like in Fig. 1d middle, with an additional linker in red). The whole movie encompasses 146 ms and shows the high intrinsic dynamics of the loops and the loop aggregate/rosette, including the fact that this single subchromosomal domain can freely rotated since it is now not constrained by other subchromosomal domains compared to the β-globin locus (Movie S2) or the IGF/H19 region (Movie S3). Hence, obviously a topological preformed architecture would dissolve within seconds if it would not be stable. Nevertheless, the loop aggregates/rosettes form distinct subchromosomal domains in which the loops do not intermingle freely (see also Figure S10) in contrast to other models such as the RW/GL model. The final shape and form in a whole nucleus would be determined by the limitations adjacent chromosomes provide.

**Additional file 15: Figure S7.** The similarity of *T2C* combined maximum/average projections of the IGF/H19 region HS 11p 15.5–15.4 HB2, HEK293T TEV (intact cohesin), and HEK293T HRV (cleaved cohesin), cell types and functional states shows that i) clearly dedicated architectural loops exist, whose ii) locations show only minor differences between the cell type or functional state, despite varying interaction frequencies whose origin (e.g. due to different crosslink-influencing binding of proteins, which even might not have a structural relevance, but influence the experimental crosslinking) and functional relevance, e.g. in terms of loop formation e.g. due to an enhancer-gene looping interaction remain still unclear. Consequently, the genome has a clear basic structural consensus architecture between different cell types or states, which is functionally altered or fine-tuned. The combination also shows, in comparison with the interaction matrices (Fig. 1b–d; Figures S1–3), that construction of an automatic loop detection algorithm is highly dependent on local conditions, loop architecture, and inter- and intra-loop interactions as thus presumably also on the local chromatin quasi-fibre compaction and architecture—all including their dynamics and functional alterations/dependencies. Thus, a one-for-all algorithm with a one-for-all parameter set to detect the loops might not exist for a genome-wide analysis.

**Additional file 16: Table S4.** General consensus loop sizes and thus position relative to the start of the first loop at the first loop base determined for human HB2, as well as HEK293T TEV (intact cohesin) and HRV (cleaved cohesin) cells of the IGF/H19 region at HS 11p 15.5–15.4. The subchromosomal domain size is calculated for domains with defined borders only from the sum of the loop sizes present.

**Additional file 17: Table S5.** General consensus loop sizes and thus position relative to the start of the first loop at the first loop base determined for mouse fetal brain (FB; inactive β-globin) and fetal liver (FL; active β-globin) cells of the β-globin locus at MM 7q E3-F1. The subchromosomal domain size is calculated for domains with defined borders only from the sum of the loop sizes present.

**Additional file 18: Table S6.** General consensus loop sizes and thus position relative to the start of the first loop at the first loop base determined for mouse MEL cells of one part of the Igh locus at MM 12q F1-F2. The subchromosomal domain size is calculated for domains with defined borders only from the sum of the loop sizes present.

**Additional file 19: Figure S8.** Simulated Multi-Loop-Subcompartment model averaged spatial distance maps with exact spatial distance $\left\langle R_{S}\right\rangle$ (first column left) and on the diagonal normalized interaction maps (all other columns) for interaction radii $\left\langle d_{i}\right\rangle$ of 30 nm, 50 nm, 70 nm, 90 nm, 110 nm, 130 nm, and 150 nm, for different model parameters ([model name]-[loop length]-[linker length]; [3, 4, 7, 8, 15, 16, 59, 87, 88]) with a resolution of ~520 bp. Visual inspection immediately reveals on a large scale clearly the formation of distinct subchromosomal domains with a clear edge and inter-domain interactions, as well as on intermediate scales the loop and rosette-like structure of the MLS model in agreement with the experiment. Again the low overlap of chromosome territories and subchromosomal domains can be seen as in general one of intrinsic MLS model properties [3, 5, 15, 55]. Thus, already all the effects seen in experimental interaction maps are in agreement with the simulations, and additionally the interactions are a function of all model parameters even in slight details considering that no nucleosomes were modelled here: i) In general the interaction degree depends on the interaction and crosslink probability, ii) the domain size, domain separation, and spacing of loops are proportional to their size (A-H), iii) the interactions between the domains depend on the linker size, the size, and number of loops, i.e. density of the rosettes (A-H). Thus, the subtle combination of density of rosettes due to loop size, loop number, chromatin fibre persistence, and the thus resulting exclusion effects, lead eventually for high numbers to spread out and shielding effects of rosettes, as well as the subtle influence on the interaction pattern between entire domains. The linker between domains and its proportionality to inter-domain interactions is as clearly visible as well as non-equilibration effects, which we deliberately show here to create an understanding of the interactions of loops at aggregate/rosette borders and similar effects. The in general large emptiness of experimental interaction maps depends on the interaction radii and thus also interaction and crosslink frequency. Since the simulations have no nucleosomal resolution, but instead a preset homogeneous chromatin fibre compaction and thus density, it becomes also clear that a random-walk of nucleosomes cannot generate such a pattern as the distinct loops would be smeared out and at least be not as clear (Fig. 1; Figures S1–3, S7). Thus, this also proves that the experimental crosslink probability, radius, and frequency can be estimated to be relatively low although since the relation contains a too complex parameter set not unambiguously fittable. Furthermore, by zooming in, one can see clearly the loop base structure within a rosette and the pattern created there in agreement with experimental findings at highest resolution.

**Additional file 20: Figure S9.** Simulated Random-Walk/Giant-Loop model averaged spatial distance maps with exact spatial distance $\left\langle R_{S}\right\rangle$ (first column left) and on the diagonal normalized interaction maps (all other columns) for interaction radii $\left\langle d_{i}\right\rangle$ of 30 nm, 50 nm, 70 nm, 90 nm, 110 nm, 130 nm, and 150 nm, for different model parameters ([model name]-[loop length]-[linker length]; [3, 4, 7, 8, 15, 16, 59, 87, 88]) with a resolution of ~520 bp. In the RW/GL model the large loops of several megabase pairs do not form distinct structures but intermingle freely in contrast to the Multi-Loop-Subcompartment model and in disagreement with the experiments (Fig. 1; Figures S1–3). This is even the case for 126 kbp loops and 63 kbp linkers although there distinct low-overlapping chromatin territories are formed. Again the general properties are found: i) In general the interaction degree depends on the interaction and crosslink probability, ii) the spacing of loops is proportional to their size (A-H), iii) the interactions between loops depend on the loop size, linker size, and the number of loops in proximity (A-H). Subtle combinations of loop size, linker size, and thus resulting exclusion effects leading eventually for high numbers to spread out and shielding effects of loops, as well as the subtle influence on the interaction pattern between entire loops appears or increases with at loop sizes smaller than 508 kbp. The linker between loops and its proportionality to inter-loop interactions is as clearly visible as well as non-equilibration effects, which we deliberately show here to create an understanding of the interactions of loops and similar effects. The in general large emptiness of experimental interaction maps depends on the interaction radii and thus also interaction and crosslink frequency. The general influence of homogenizing random-walk topologies becomes also obvious and again although due to the non-nucleosomal resolution, but instead the preset homogeneous chromatin fibre compaction and thus density it becomes also clear that a random-walk of nucleosomes cannot generate such a pattern as originating from the loops. Again the simulations prove that the experimental crosslink probability, radius, and frequency can be estimated to be relatively low although since the relation contains a too complex parameter set not unambiguously fittable. Besides, by zooming in, one can see clearly that the loop base structure of large loops is not as defined as within a rosette, since the interaction probability in a loop aggregate/rosette is reduced and thus more defined (Figure S8).

**Additional file 21: Supplemental Results.**

**Additional file 22: Table S7.** Simulated chromosome models with their physical properties (as in detail described Knoch [3, 4, 87, 88]): The band number is the number of subcompartments or loops per chromosome. The average theoretic loop size is $\left\langle R_{L}\right\rangle = \sqrt {\left( {300nm} \right)^{2} \cdot L_{S} /\left( {2 \cdot 300nm} \right)}$ and was determined from simulated position-dependent (PD) and position-independent (PI) spatial distances for genomic separations at half the loop size. The average simulated band size is the average extension of the mass distribution. The average theoretic band distance is $\left\langle R_{B}\right\rangle = \sqrt {\left( {300nm} \right)^{2} \cdot LI_{L} /\left( {300nm} \right)}$ and was simulated from the average spatial distance averages between succeeding subcompartments. The average theoretic territory size is $\left\langle R_{Ltotal}\right\rangle = \sqrt {\left( {300nm} \right)^{2} \cdot \left( {NB - 1} \right) \cdot LI_{L} /\left( {300nm} \right)}$ and was simulated from the average mass distribution extension. Naming of models: [model name]-[loop length]-[linker length].

**Additional file 23: Figure S10.** Simulated chromatin models description and relation/evaluation of spatial distances between genomic markers, in the Immunoglobulin Heavy Chain locus and the Prader-Willi/Angelmann Syndrome region [3, 4, 7, 8, 59]: A, Volume-rendered images of simulated Random-Walk/Giant-Loop and Multi-Loop-Subcompartment models. As a starting conformation with the form and size of a metaphase chromosome (top), rosettes were stacked (α). From such a starting configuration, interphase chromosomes in thermodynamic equilibrium were decondensed by Monte Carlo and relaxing Brownian Dynamics steps. A volume rendered image of the simulated Random-Walk/Giant-Loop model containing large loops (5 Mbp) is shown (left; β). Note that the large loops do not form distinct structures but intermingle freely (left; β). In contrast, in a volume-rendered image of the simulated Multi-Loop-Subcompartment model, containing 126 kbp sized loops and linkers, the rosettes form distinct chromatin territories in which the loops do not intermingle freely (middle; γ). In an image of the simulated RW/GL model containing 126 kbp loops and 63 kbp linkers, again distinct chromatin territories are formed but in contrast to the MLS model no subcompartments form (right; δ). B, Strategy for position-dependent and position-independent virtual spatial distance measurements in the simulations: For position-dependent virtual distance measurements, the first marker was placed close to the base of the loop (marker 1). The virtual spatial distances were measured from this “viewpoint” to other makers on the chromatin fibre, e.g. in the rosette (1–7) and to a linker (8–10). For position-independent measurements a set of markers separated by the same genomic distance were randomly positioned (x, y, z). C, Comparison between simulated position-dependent (dotted lines) and position-independent (solid lines) spatial distances. The curves (A-D) indicate simulated MLS models with 126 kbp loops and different linker sizes. RW/GL is shown for comparison (A). Position-dependent distances (dotted lines) show a stepwise increase in the region where a linker is connecting two chromatin subcompartments, while position-independent distances (solid lines) do not show the stepwise increase in spatial distances as a function of genomic separation. D, Random-Walk/Giant-Loop and Multi-Loop-Subcompartment models: α indicates the RW/GL model in which large loops are attached to a non-DNA backbone. β shows the simulated model containing a chromatin linker between loops. The MLS model is shown containing 126 kbp loops and linkers with individual rosettes spanning 1–2 Mbp. E, The simulated spatial distances as a function of genomic separation are shown for a fixed loop structure. The simulated loop size was 126 kbp. Two virtual genomic markers were chosen that were separated by 252 kbp. The coloured spatial distance map indicates the frequency distribution of simulated spatial distances. F and G, Comparison between experimental data and computer-simulated data obtained from spatial distance measurements in the Igh locus [7] and the Prader-Willi/Angelmann Syndrome region [8] as a function of genomic separation (resolution of the simulation model is 5.2 kbp, i.e. the base pair size of the polymer segments from which the simulations are setup). Nomenclature is loop size [kbp]-linker size [kbp]-topology. Experimental spatial distance measurements [mm] were plotted as a function of genomic separation [Mbp] in the Igh locus for pre-pro-B cells (blue dots and green circles) and pro-B cells (red squares and pink triangles), and in the Prader-Willi/Angelmann Syndrome region for fibroblasts for either structure preserving para-formaldehyde fixation (FAA; black full circles and triangles) or structure destroying methanol acetic acid fixation (MAA; black open circles and triangles) using λ-probes (black full circle and triangles) and BAC-probes (black open circle and triangles). The comparison shows clearly, best agreement for a multi-loop aggregate/rosette like model, and even clearly structure destroying MAA fixation (see the λ-probes data for very low genomic separation), even for genomic separations at 650 kbp can only anticipate RW-GL models with loops smaller than ~500 kbp.

**Additional file 24: Figure S11.** Description of the measurement process (A), and spatial-distance *D*_*SD*_ and yard-stick dimensions *D*_*Y*_ of simulated single chromosomes (B-E). The spatial-distance dimension was determined from position-independent spatial distances as function of randomly positioned genetic markers with a genomic/curvature separation *c*_*SD*_. Thus, markers could reside both on the same loop (**a-b**), on different loops (**a-c**), on a loop and in a linker (**b-d**), both in the linker (**d-e**), or on loops belonging to different rosettes (**b-f**). The exact yard-stick dimension *D*_*Y*_ was calculated by walking along the fibre using a yard-stick *l*_*Y*_. Thus, the start and end of a small *l*_*Y*_ mostly reside in the same loop (**1–16**) in contrast to large *l*_*Y*_ often lying in different loops (**1–6**) or rosettes (**1–3**). The end point of *l*_*Y*_ was determined exactly by finding the chain segment, where $L_{1} < l_{Y } < L_{2}$, before solving the corresponding vector equation. The spatial distance function $R_{SD} \left( {c_{SD} } \right)$ (B, C) and exact yard-stick curve length function *C*_*SD*_(*l*_*SD*_) (D, E) shows power-law behaviour as expected for fractal self-similar polymer foldings. The slopes are the spatial distance dimension $D_{SD}$ and the exact yard-stick dimension *D*_*Y*_. The finite size of chromosomes generates a cut-off > ~80 Mbp or > ~8 μm after which the power-law behaviour breaks down. Additionally, non-trivial power-law behaviour due to the deviation of $D_{SD}$ and *D*_*Y*_ from 1.0 (a stiff linear segment) or ~2.0 (a random-walk), four major scaling regions exist. The detailed dimension behaviour is given by the local dimensions $D_{SD} \left( {c_{SD} } \right)$ and *D*_*Y*_(*l*_*y*_) (C, E) with fluctuations the bigger the closer to the cut-off. The general multi-scaling behaviour of $D_{SD}$ and *D*_*Y*_ is characterized by an increase from an initial 1.0 for small $c_{SD}$ and *l*_*Y*_ and characterizing the stiff chromatin segments, over values ~2.0 as for the random-walk of the segments to a maximum of 3.0 stating the ring-shaped loops of both the MLS and the RW/GL model and globular state of the rosettes of the MLS models according to the $c_{SD}$ and *l*_*Y*_. In the MLS model thereafter again local dimensions ~2.0 are reached describing the random organization of the rosettes relative to each other. Within the general behaviour a fine structure attributable to the loops aggregated in rosettes is present for MLS models, better measured with *D*_*SD*_. The maximum position and height are proportional to the loop and linker size in the MLS and the RW/GL model, and all the inherent model properties are visible. Naming of models: [model name]-[loop length]-[linker length].

**Additional file 25: Figure S12.** Simulated Multi-Loop-Subcompartment interaction scaling curves *I*(*s*) shown here for clearness only for interaction radii $\left\langle d_{i}\right\rangle$ of 30 nm, 70 nm, 110 nm, and 150 nm for different model parameters ([model name]-[loop length]-[linker length]; [3, 4, 7, 8, 15, 16, 55, 82, 83]) with a resolution of ~520 bp. The slopes would be the interaction coefficient *ι*(*s*). Due to the high resolution the general behaviour and fine-structural features of long-range interaction scaling as a function of the genetic separation *s* becomes, however, immediately clear in contrast to the spatial-distance function $R_{SD} \left( {c_{SD} } \right)$ (B, C) and exact yard-stick curve length function *C*_*SD*_(*l*_*SD*_) (Figure S8). *I*(*s*) shows long-range power-law behaviour as expected for fractal self-similar polymer foldings. The finite size of chromosomes generates a cut-off > ~1 to ~10 Mbp depending on the interaction radius after which the power-law behaviour breaks down. The detailed power-law behaviour is characterized by different behaviours on different scales attributable to the rosette-like subcompartments in the MLS model and the scale above where the arrangement of subcompartments into the chromosome territory by a random linker walk is visible. Within the general behaviour the fine structure attributable to the loops aggregated in rosettes is clearly visible in detail for MLS models. The pronouncedness of the fine structure is averaged out by higher interaction radii and thus shows also what happens if experimental fragment sizes are in- or decreased. Thus, already all the effects seen in simulated interaction maps are here again a function of all model parameters even in slight details considering that no nucleosomes where modelled here: i) In general the scaling degree depends on the interaction and crosslink probability, ii) the domain size, domain separation, and spacing of loops are proportional to their size, iii) the interactions between the domains depend on the linker size, the size and number of loops, i.e. density of the rosettes.

**Additional file 26: Figure S13.** Simulated Random-Walk/Giant-Loop interaction scaling curves *I*(*s*) shown here for clearness only for interaction radii $\left\langle d_{i}\right\rangle$ of 30 nm, 70 nm, 110 nm, and 150 nm for different model parameters ([model name]-[loop length]-[linker length]; [3, 4, 7, 8, 15, 16, 57, 87, 88]) with a resolution of ~520 bp. The slopes would be the interaction coefficient *ι*(*s*). Due to the high resolution the general behaviour and fine-structural features of long-range interaction scaling as a function of the genetic separation *s* becomes, however, immediately clear in contrast to the spatial-distance function $R_{SD} \left( {c_{SD} } \right)$ (B, C) and exact yard-stick curve length function *C*_*SD*_(*l*_*SD*_) (Figure S11). *I*(*s*) shows long-range power-law behaviour as expected for fractal self-similar polymer foldings, with mainly one general behaviour due to the arrangement of loops into the chromosome territory by a random linker walk. The finite size of chromosomes generates a cut-off > ~1 to ~ 10 Mbp depending on the interaction radius after which the power-law behaviour breaks down. Within the general behaviour the fine structure attributable to the loops which are clearly not clustered in rosettes is clearly visible in detail for RWGL models. Thus, again already all the effects seen in simulated interaction maps are here again a function of all model parameters even in slight details considering that no nucleosomes where modelled here: i) In general the scaling degree depends on the interaction and crosslink probability, ii) the domain size, domain separation, and spacing of loops are proportional to their size, iii) the interactions between the domains depend on the linker size, the size and number of loops, i.e. density of the rosettes.

**Additional file 27: Figure S14.** Experimental interaction scaling curves derived from *T2C* for the human IGF/H19 11p 15.5–15.4 region in HB2, HEK293T TEV, and HEK293T HRV (A-C) cell systems, as well as the mouse β-globin locus 7qE3-F1 for fetal brain and liver cells using a 3 bp average of the raw data from 1 to 200 bp (red) and thereafter in the polymer regime a combination of a grouping with a 1% resolution per order of magnitude and an added running window smoothing average to get more general characteristics for raw (blue) as well as “secured” (i.e. only 100% non-neighbouring or restricted and re-ligated interactions are used; light blue). The values <10 bp are in principle due to the algorithm used and for transparency not discarded since they nevertheless show the extrapolation from existing values >10 bp. In all cases the interactions show long-rang scaling with multi-scaling on different scales as well as a fine structure on top (Fig. 2a). From 1–195 bp the behaviour and fine structure is associated with the nucleosomal winding of the DNA around the nucleosome, i.e. the 1.7 and 145 bp (the peak of interactions) winding of the DNA double helix around the nucleosomal core and the nucleosomal repeat length of 195 bp (Fig. 2d; Figure S15) and its dedicated fine structure as seen also in the DNA sequence correlation multi-scaling power-law correlations with its fine structure (Fig. 1e; Figures S16–21). This is despite the used resolution of the here used restriction enzymes visible in the unsecured data and also dependent on the used restriction enzyme. Thereafter, i.e. for scales >195 bp there is a clear plateau-like increase up to a “peak” at ~10^3^ bp, which is clearly indicating that the nucleosomes interact equally or even more up to a scale of ~10^3^ bp, i.e. that on average a chromatin quasi-fibre is formed with 5±1 nucleosomes per 11 nm, since a nucleosome has a dimension on that scale and we see a clear decrease of interactions after a scale of 6 nucleosomes, i.e. ~1.0–1.2 bp. Whether the fluctuations within this regime can be associated with the order of nucleosomes within the fibre conformation is yet hard to say. In the case of “secured” interactions the plateau and increase to the peak are seen, thus this is a real effect and not e.g. due to neighbouring or unrestricted DNA fragments. Thereafter, there is a multi-scaling behaviour with three regimes: i) The first regime until ~10^4^ bp still shows the chromatin quasi-fibre formation before it goes over into the ii) regime of chromatin fibre and chromatin loops with a slightly higher decrease in interactions, which then transits iii) to a nearly flat plateau indicating the formation of an aggregated state of rosettes in agreement with polymer simulations (Fig. 2b; Figures S9–13). Thereafter, we see a sharp decrease due to the limits of the regions for which the experiments were done, i.e. ~2.1 Mbp. This entire multi-scaling behaviour is not only in agreement with polymer models (Fig. 2b; Figures S10–15), but furthermore there is a fine structure which can be associated with the loop sizes already determined in the interaction matrices (of course, the loops are not as clearly visible as in the simulations due to the variation of the loop sizes, but clear peaks around the average experimental loop sizes is clearly visible). Even beyond that, the entire behaviour is also in agreement with the fine-structured multi-scaling long-range power-law correlations of the DNA sequence itself (Fig. 2e; Figure S16–21).

**Additional file 28: Figure S15.** Experimental interaction scaling curves derived from *T2C* for the high-resolution data derived from mouse MEL cells for 15 loci covering in total ~99 Mbp with a subnucleosomal fragment resolution (Table S2) show clearly a fine structure associated with the nucleosome as shown for the average over all chromosomes (A-C): In general there is an increase of the interaction until a plateau is reached from ~50 bp to ~100 bp (A, B), thereafter there is a sharp peak which is ~1.5 orders of magnitude higher from ~110 to 195 bp with a width of ~85 bp, followed by a slight decent to a second “dip/plateau” from ~230 bp up to the transit to a new descent at ~10^3^ bp which then obviously transits at ~10^4^ bp to the known multi-scaling behaviour seen in the lower resolved human and mouse cases (Figure S14). On this behaviour there is a fine structure (A-C) which can be associated with the binding of the DNA double helix to the nucleosomal core (Figure S14): in the first plateau many peaks are agreeing with those (Figure S16) of the fine structure found in correlations of the DNA sequence itself, on top of the peak a clear fine structure at 145 bp can be found and again many of the features agree with correlations of the DNA sequence itself (Figure S16; and [5, 15, 16]). Additionally, the plateau from 195 bp to 1200 shows also characteristic features, e.g. at 290 bp as well as at 385 bp the peaks are exactly where di-nucleosomal features are expected. This is logical since neighbouring nucleosomes might see each other most likely/often. Astonishingly, the plateau itself only decreases by ~10%, i.e. nucleosomes 4–6 see the first nucleosome with nearly the exact same probability, which suggests an average quasi-fibre, with a packing density of on average 5±1 nucleosomes per 11nm, since the nearest proximity of the 4–6 or any other subsequent nucleosome cannot be smaller than the nucleosome core itself. Since there is a slight dependency with respect to the used (Figure S14) restriction enzyme with lower resolution, there might be also dependencies here, which might, however, be much smaller due to the high resolution achieved here.

**Additional file 29: Figure S16.** The fine-structural features of *Homo sapiens* and *Homo sapiens GRCH37* as well as *Mus musculus* and *Mus musculus C57BL6j*, survive averaging over all chromosomes (as previously shown (Figure S17; and [3, 5, 15, 16, 40, 59]). The very pronounced local maximum at 11 bp is related to the double helical pitch in both species, whereas the local minima and maxima are very clearly related to the nucleosome in the much more pronounced human case, which is e.g. obvious for 146 bp, but less obvious for 172 bp, 205 bp, 228 bp and 248 bp. This fine structure present in all human sequences is in agreement with the pattern found in simulations using a consensus nucleosomal binding sequence organized in a block/gene fashion, and the positions of the local maxima are mostly the same as in the human genome, whereas the similarity of the position of the local minima is difficult to compare as they smear out in the human sequence due to the block structure of genomes [5, 59]. In mouse, the general behaviour of the fine structure is different and not as pronounced compared to the human case, a close inspection reveals that many of the fine structural peaks although small are also present, at least in individual chromosomal sequences. Thus consequently, the concentration of nucleosomal binding sites seems to be less and differently distributed in mouse compared to human sequences and also might have an evolutionary different survival time within the DNA sequence [3, 5, 15, 16, 40, 59].

**Additional file 30: Figure S17.** Introduction to the correlation function *C*(*l*) and the correlation coefficient *δ*(*l*) for the averages over all chromosomes for each “strain-specific” sequencing of *Homo sapiens*, *Homo sapiens GRCH37*, *Mus musculus*, and *Mus musculus C57BL6j* (from the http://www.ebi.ac.uk/genomes/eukaryota.html genome list; [3, 5, 15, 16, 40, 59]): A: The correlation function *C*(*l*) of random sequences shows power-law behaviour as expected for a fractal self-similar sequence. All results are numerically exact. The slope is the correlation coefficient *δ*(*l*) whose value in the linear region is -0.5 (yellow line), which resembles the theoretic value and thus indicating random correlations. The finite sequence length generates a cut-off after which the power-law behaviour breaks down, thus concatenation of two sequences creates a double cut-off. Sequences of *Homo sapiens*, *Homo sapiens GRCH37*, *Mus musculus*, and *Mus musculus C57BL6j* exhibit even after averaging over all chromosomes for each strain not only a positively correlated power-law behaviour due to a *δ*(*l*) bigger than -0.5 (B), but also four regions (numbers 1–4) with different degree of correlation for 10^8^ bp. B: The detailed correlation behaviour is given by the local correlation coefficient *δ*(*l*), which fluctuates around -0.5 for random sequences. The fluctuations become bigger as the window size approaches the cut-off. *Homo sapiens*, *Homo sapiens GRCH37*, *Mus musculus*, and *Mus musculus C57BL6j* again for the averages over all chromosomes per strain, reveal a distinct positively correlated pattern with less fluctuations. In general, *δ*(*l*) increases from a starting value until a plateaued maximum, before a decrease and a second statistically significant maximum. Finally, *δ*(*l*) decreases to values characteristic for random sequences and enters the region of fluctuation. Within this general behaviour, a distinct fine structure is visible more dominantly in the human compared to the mouse case, which survives averaging over different locations within the chromosomal sequences and even over all chromosomes of each strain. The very pronounced local maximum at 11 bp is related to the double helical pitch, whereas the local minima and maxima are related to the nucleosome, which is obvious for 146 bp, but less obvious for other positions e.g. at 172 bp, 205 bp, 228 bp, and 248 bp (Figure S16). The second maximum around 10^5^ is related to chromatin loops of the three-dimensional genome organization and its grouping in aggregates/rosettes. Thus, the 4 regions in *C*(*l*) (A) can be associated with i) the nucleosome, ii) the compaction of the nucleosome chain into a compacted fibre, iii) the formation of loops of the chromatin fibre, and iv) the arrangement of subchromosomal domains in the entire chromosomes. The differences between mouse and human are mainly an earlier ascent in the case of mouse and a lower plateau in comparison with human sequences (A, B). The differences between the strains within one species are mainly due to differences in the quality of sequences, e.g. unfinished/partial sequencing of the Y-chromosome, and also, but to a less degree, due to real sequence differences as genome rearrangements. C, D: To distinguish real from statistical correlations, the standard deviation was computed from 20 random sequences with similar base pair distribution as in *Homo sapiens* for *C*(*l*) (c) and *δ*(*l*) (D). The standard deviation of *δ*(*l*) shifts only to higher window sizes depending on the sequence length. E, F: For the real sequences the standard deviation for *C*(*l*) (E, in absolute (thin lines) and relative (thick lines) terms, i.e. dividing the standard deviation $StDev C\left( l \right)$ by the average over all chromosomes of a strain 〈*C*(*l*)〉 according to $StDev C\left( l \right)_{relative} = StDev C\left( l \right)/\left\langle {C\left( l \right)} \right\rangle$), and *δ*(*l*) (f) does not increase so much with growing window sizes as in the case of random sequences due to the fact that real genomes have never an entire random sequence organization due to their evolutionary construction.

**Additional file 31: Figure S18.** A-H: General scaling behaviour of the correlation function *C*(*l*) in *Homo sapiens* and *Homo sapiens GRCH37* strains showing clear power-law behaviour with four clearly distinct regions (Figure S11) of different correlation degree, while approaching the finite sequence length generates a cut-off after which the power-law behaviour breaks down. In most cases differences between chromosomes are bigger than between strains, despite the obvious differences in length of several chromosomes (e.g. *Homo sapiens GRCH37* Y-chromosome) mainly due to differences in the quality of sequences, e.g. unfinished/partial sequencing of the Y-chromosome, and also, but to a less degree, due to real sequence differences as genome rearrangements. Apparently, the differences grow with growing window size *l* (and thus the scale), but appear mainly for *l* >10^6.5^–10^7^ bp due to approaching the upper cut-off, with the exception of chromosomes 22, X, and Y, where the differences appearing at *l* already >10^3^ bp pointing to a general bigger difference in the general sequence organization with respect to the other chromosomes. This special behaviour as well as the general scaling behaviour is nearly identical with the two mouse strains *Mus musculus* and *Mus musculus C57BL6j*, thus this is a general feature of those chromosomes across species.

**Additional file 32: Figure S19.** Detailed multi-scaling behaviour of the correlation coefficient *δ*(*l*) and its fine-structural features for *Homo sapiens* and *Homo sapiens GRCH37*: The correlation coefficient *δ*(*l*) shows strong positive correlations for human chromosomes (A-H). In general, *δ*(*l*) increases from a starting value until a plateaued maximum from 10^2^–10^3.6^ bp, before a decrease and a second statistically significant maximum at ~10^5^ bp for all chromosomes of both strains. Finally, *δ*(*l*) decreases to values characteristic for random sequences and enters the region of fluctuation. The differences between the strains within one species are mainly due to differences in the quality of sequences, e.g. unfinished/partial sequencing of the Y-chromosome, and also, but to a less degree, due to real sequence differences as genome rearrangements. Within this general behaviour, a distinct fine structure visible more dominantly in the human compared to the mouse case is present in all chromosomes and survives averaging (Figures S16, S17B). The very pronounced local maximum at 11 bp is related to the double helical pitch, whereas the local minima and maxima are related to the nucleosome, which is obvious for 146 bp, but less obvious for other positions e.g. at 172 bp, 205 bp, 228 bp and 248 bp. The second maximum around 10^5^ shows also a fine structure which is due to the individual chromatin loops of the three-dimensional genome organization and their grouping in aggregates/rosettes.

**Additional file 33: Figure S20.** A-G, General scaling behaviour of the correlation function *C*(*l*) in *Mus musculus* and *Mus musculus C57BL6j* showing clear power-law behaviour with four clearly distinct regions (Figure S17) of different correlation degree, while approaching the finite sequence length generates a cut-off after which the power-law behaviour breaks down. In most cases differences between chromosomes are bigger than between strains, despite the obvious differences in length of several chromosomes (e.g. *Mus musculus C57BL6j* Y-chromosome) mainly due to differences in the quality of sequences, e.g. unfinished/partial sequencing of the Y-chromosome, and also, but to a less degree, due to real sequence differences as genome rearrangements. Apparently, the differences grow with growing window size *l* (and thus the scale), but appear mainly for *l* >10^6.5^–10^7^ bp due to approaching the upper cut-off, with the exception of chromosomes 22, X, and Y, where the differences appearing at *l* already >10^3^ bp pointing to a general bigger difference in the general sequence organization with respect to the other chromosomes. This special behaviour as well as the general scaling behaviour is nearly identical for the two human strains *Homo sapiens* and *Homo sapiens GRCH37*, thus this is a general feature of those chromosomes across species.

**Additional file 34: Figure S21.** Detailed multi-scaling behaviour of the correlation coefficient *δ*(*l*) and its fine-structural features for *Mus musculus* and *Mus musculus C57BL6j*: The correlation coefficient *δ*(*l*) shows strong positive correlations for human chromosomes (A-G). In general, *δ*(*l*) increases from a starting value until a plateaued maximum from 10^2^–10^3.6^ bp, before a decrease and a second statistically significant maximum at ~10^5^ bp for all chromosomes of both strains. Finally, *δ*(*l*) decreases to values characteristic for random sequences and enters the region of fluctuation. The differences between the strains within one species are mainly due to differences in the quality of sequences, e.g. unfinished/partial sequencing of the Y-chromosome, and also, but to a less degree, due to real sequence differences as genome rearrangements. Within this general behaviour, a distinct fine structure visible less dominantly in the human compared to the mouse case is present in all chromosomes and survives averaging (Figures S16, S17B). The very pronounced local maximum at 11 bp is related to the double helical pitch, whereas the local minima and maxima are related to the nucleosome, which is obvious for 146 bp, but less obvious for other positions. The second maximum around 10^5^ shows also a fine structure which is due to the individual chromatin loops of the three-dimensional genome organization and their grouping in aggregates/rosettes.

**Additional file 35: Figure 1.** *T2C* description, interaction mapping, and direct determination of the chromatin quasi-fibre and the aggregated loop/rosette 3D architecture of the human and mouse genomes: **a** Cell nuclei in a population of cells (transmission light and fluorescence microscopy, [89]) have an underlying chromatin architecture (simulated cell nucleus containing 1.2 million polymer segments; resolution 5.2 kbp, i.e. ~50 nucleosomes; Multi-Loop-Subcompartment (MLS) rosette model with 126 kbp loops and linkers; [5]). After crosslinking the DNA is restricted within the nucleus by a 1st restriction enzyme, before the crosslinked fragments are extracted and diluted such that intra-fragment re-ligation is favoured. After de-crosslinking, the re-ligated material is shortened by a 2nd restriction enzyme or sonication and purified by a capture array with oligos designed next to the 1st restriction enzyme, before paired-end-sequencing over the ligation position. After alignment to the reference genome, this results in interactions frequency matrices (b–d) and scaling curves (Fig. 2). **b**, **c** Interaction matrices (logarithmic and colour-coded scale; *left* and *right*) of the human IGF/H19 11p 15.5-15.4 region (**b**) in HB2, HEK293T TEV (intact cohesin) and HEK293T HRV (cleaved cohesin) as well as the mouse β-globin 7qE3-F1 region (**c**) for fetal brain (inactive β-globin) and liver cells (active β-globin) show the formation of subchromosomal domains separated by a linker (borders: *pink lines, right*; D1s, D1e: start and end of domains), which consist of loops (*red lines*; 8L: number of loops), representing due to the grid-like pattern loop aggregates/rosettes. A grid-like pattern is also visible in the interactions between the domains and corresponds to the interactions of loops and loop bases of interacting domains. Near the diagonal the aggregation into a chromatin quasi-fibre and loop internal structures are visible (zooming in and out the images can make this clearer). Between different cell types and functional states only some local differences are visible resulting in a consensus architecture and allowing simulation of the 3D architecture (middle; resolution <~1 kbp). Note that the simulation is driven by the dominant consensus architecture. **d** The interaction matrix of a 380 kbp subchromosomal domain in the mouse 12qF1–F2 region at high resolution clearly shows the regular rosette-like picture with a detailed structure of the loop base with in- and outgoing loop fibre stretches as seen in simulations (**e**, **f**). **e** Simulated Multi-Loop-Subcompartment (MLS) model with an averaged spatial distance map for exact spatial distances 〈*R*_*S*_〉 (*left*) and on the diagonal normalized interaction maps for interaction radii 〈*d*_*i*_〉 of 50 nm, 70 nm, and 150 nm (*right*), for an MLS model with 126 kbp loops and linkers [16 Mbp upper and 1.2 Mbp zoom-in (*z*) lower row), showing clearly the formation of domains connected by a linker, their interaction, and the underlying loop aggregates/rosette architecture, with (anti-)parallel fibre stretches at the loop base. The dependence on the interaction radii corresponding to different crosslink probabilities is also clearly visible. **f** Sketch of the different structures visible on different scales in the experimental and simulated interaction matrices (spatial distance matrix: *left*; simulated interaction matrix: *upper*) (from **e**): On the smallest scale, near the diagonal the compaction of nucleosomes into the quasi-fibre (*yellow line*) and the fibre regime (*dark blue line*) can be found. On the largest scale the domains are clearly bordered (*pink lines*) and connected by a linker. On medium scales the loop aggregate/rosette like structure is characterized by the loop bases (*red circles*: within domains, *blue circles*: between domains) as well as the loop interactions (*green triangles*). The fine structure of the loops representing the (anti-)parallel loop stretches at the base (*red crosses*) and within loops (*green stretches near diagonal*).

**Additional file 36: Figure 2.** Scaling analysis of experiments, simulations, and the DNA sequence showing the formation of a chromatin quasi-fibre and the loop aggregate/rosette genome architecture: **a** The fine-structured multi-scaling resulting from the *T2C* interaction frequency as a function of the genomic separation for the human IGF/H19 11p 15.5–15.4 region and the mouse β-globin locus 7qE3–F1 (3 bp average (1–200 bp) and thereafter a grouping with a 1 % resolution per order of magnitude which for clarity is smoothed by a running window average for >10^3^ bp; see also Additional file 27: Figure S14; the values <10 bp are due to the algorithm used and for transparency not discarded since they nevertheless show the extrapolation from values >10 bp), shows: (i) The structure of the nucleosome, (ii) the formation of a plateau from 195 to ~1000 bp, indicating the formation of a chromatin quasi-fibre with a density of 5 ± 1 nucleosomes per 11 nm, (iii) the chromatin quasi-fibre regime, (iv) a mixed chromatin fibre/loop regime with a slightly higher interaction decrease, (v) the plateau indicating the loop aggregate/rosette state, and (vi) in principle the linker regime (not visible in **a** but in **d**). **c**, **d** The fine-structured multi-scaling is even clearer for the average of 15 loci covering in total ~99 Mbp in mouse MEL cells with subnucleosomal fragment resolution: After an initial increase a plateau is reached from ~50 to ~100 bp, followed by a sharp peak from ~110 to 195 bp (width at plateau level ~85 bp), followed by a second ~10 % decreasing plateau up to 1.0–1.2 kbp, which after a sharp decent until ~10^4^ bp transits to the known multi-scaling behaviour (**d**, compare with **a**). With this resolution the fine structure visible (Additional file 28: Figure S15), can be associated with the binding of the DNA double helix to the nucleosome, since up to ~195 bp many of the small peaks (the most prominent at 145 bp) can be associated with the fine structure in the fine-structured multi-scaling behaviour of DNA sequence correlations (**e**; Additional file 28: Figure S15, Additional file 29: Figure S16). Whereas the structure of the nucleosome vanishes using “secured” interactions (**c**, *pink* and *light blue*), above 195 bp the plateau and multi-scaling behaviour remain. Again the values <10 bp are due to the algorithm used and for transparency not discarded since they nevertheless show the extrapolation from values >10 bp. **b** The interaction scaling of a simulated Multi-Loop-Subcompartment model with 126 kbp loops and linkers as well as a Random-Walk/Giant-Loop model with 1 Mbp loops and 126 kbp linkers consistently shows for different interaction radii a multi-scaling behaviour. The MLS model shows the characteristic rosette plateau, followed by the random scaling regime of the linker conducting a random-walk. The peaked fine structure originates from the loops forming the rosettes. In contrast, the RWGL model is characterized by random-walk regime and only one major fine structure attributable to the single loops. At greater scales the limit of the entire chromosome is seen in the cut-off. The MLS model agrees in detail with experiments (**a**, **c**–**d**) and the DNA sequence organization (**e**). **e** The fine-structured multi-scaling long-range correlation behaviour of each of two human and mouse strains shows clearly again the architectural features: a general increase until a plateaued maximum (including the 145 bp peak), a first plateau area until ~1200 bp, transition to a sharper decrease at ~3.6 kbp (the sweet point used in the calculation of the persistence length) until a minimum ~10–20 kbp and a second statistically significant maximum at ~100 kbp, followed by a random regime and a final cut-off. The first maximum and plateau are characteristic for the nucleosome and formation of the quasi-fibre (**c**; Additional file 28: Figure S15, Additional file 29: Figure S16) which then transits to chromatin loops and their clustering into loop aggregates/rosettes which are connected by a random-walk behaving linker. Thus, due to the higher statistics here, the architectural features and their tight representation within the DNA sequence organization are even clearer.
