# Supplementary material for: The detailed 3D multi-loop aggregate/rosette chromatin architecture and functional dynamic organization of the human and mouse genomes
Source: Epigenetics Chromatin. 2016 Dec 24;9:58. doi: 10.1186/s13072-016-0089-x (PMC5192698; doi:10.1186/s13072-016-0089-x)
Supplement: Supplementary file 1 — Additional file 1: Supplemental Methods. [file 13072_2016_89_MOESM1_ESM.docx]

**Supplemental Methods:**

*HB2 Cell Line and Cell Culture*

HB2 cells (1-7HB2, a clonal derivative of the human mammary luminal epithelial cell line MTSV1-7 [S3] were cultured in DMEM supplemented with 0.2 mM L-glutamine, 100 units/ml penicillin, 100 mg/ml streptomycin, 10% FCS, 5 µg/ml hydroxycortisone, and 10 µg/ml human insulin. In a previous 3C study we confirmed the karyotype and the DNA methylation of several regions [S4].

*Cohsin Cleavage HEK293T TEV/HRV Cell Line System and Cell Culture*

The cleavable HEK293T TEV/HRV RAD21-eGFP stable cell line system (Schockel et al., 2011) is a HEK293T cell line system, which was transfected with a pRTS-1 vector [S5] encoding for a cleavable RAD21-eGFP fusion protein and an siRNA for endogenous RAD21 knock-down ([83,S6] and references in there). Both are expressed by doxycycline induced activation of a bidirectional promoter in-between and thus simultaneously. For the RAD2-eGFP fusion-protein, a cleavable RAD21, where the first RAD21-separase cleavage site is replaced by that of the 3C protease of the human rhinovirus (HRV protease) using a PCR-based mutagenesis (the second cleavage site remained unchanged to ensure less cell cytotoxicity) was inserted before eGFP. The tobacco etch virus protease (TEV protease), does not recognize the HRV cleavage site and thus can act as a control. The endogenous RAD21 knock-down sequence allows knock-down with the following 3’UTR-directed siRNA’s:

5′-ACUCAGACUUCAGUGUAUA-3′ (Scc1-1),

5′-AGGACAGACUGAUGGGAAA-3′ (Scc1-2).

For generating the HEK293T TEV/HRV RAD21-eGFP cell line system the original HEK293T cell line was cultured in DMEM supplemented with 0.2mM L-glutamine, 100 units/ml penicillin, 100 mg/ml streptomycin, 10% FCS, and was grown at 37°C and 5% CO_2_. For the transfection Lipofectamine 2000 (Invitrogen) according to the instructions of the manufacturer was used. Cells carrying the vector were selected by growth in 150 µg/mL hygromycin containing medium. Single clones were picked and analysed for expression of RAD21cv and RAD21wt constructs and depletion of the endogenous RAD21 three days after induction with 2 µg/ml doxycycline. The resulting HEK293T TEV/HRV RAD21-eGFP cell line was as well cultured in DMEM supplemented with 0.2mM L-glutamine at 37°C and 5% CO_2_.

To activate transgene expression with HRV (or TEV which serves as a control, thus a transfection takes place, but no cleavage) the cells were cultured for 3 days in the presence of 2 µg/ml of doxycycline. Thereafter, cells were split, reseeded until 50% confluency and transfected with HRV or TEV vectors using Lipofectamine 2000 (Invitrogen) again according to the instructions of the manufacturer. 24 hours after protease transfection the cells were used for the experiment.

*Cell Preparation from Mice*

For mouse fetal liver and fetal brain cells, ~10 embryos on day 12.5 of pregnancy from one to two transgenic FVB/N mice were used for the ~10 million cells required by the experiment to have a complex enough cell population and enough DNA in the end to be sequenced: Mice were cleaned with 70% EtOH and the abdomen was opened to remove the cervix containing the embryos, before cutting them lose and removing them from the yolk sac and placenta. Small and underdeveloped embryos were discarded. The embryos are collected in petri dishes on ice with 0.5 ml 10% FCS/PBS. Then the fetal liver and brain were dissected from the embryos and collected in tubes (1 ml) on ice containing again 500 µl 10% FCS/PBS. The cells were then resuspended with a P1000/1ml plastic pipette tip and connective tissue was digested by adding 25 μl of a 2.5% Collagenase stock (0.125% end concentration) and incubated for ~ 45 min at 37°C. Thereafter, the cell suspension was transferred to falcon tubes with 12 ml 10% FCS/PBS at room temperature and then was gently squeezed through a scraper mesh which was placed inside a 6-well dish using again a P1000/1ml plastic pipette tip. The mesh was washed with 2ml 10% FCS/PBS at room temperature to get all the cells from the mesh. The resulting single cell suspension was again collected in falcon tubes with an end volume of 12 ml 10%FCS/PBS at room temperature. Notably, we tried to keep the stress of the cells to the minimum, to avoid any damage to the cell nuclei. Both after the resuspension, the Collagenase treatment, and/or after the scraping of fetal liver and brain material/cells were spotted on glass slides to check for cellular and especially nuclear integrity by microscopy, with or without staining of the nucleus with DAPI (or eventually any other immunofluorescence or fluorescence *in situ* hybridization).

*T2C Crosslinking/Fixation of Cells*

For crosslinking/fixation of the genome and the entire cells, the cells were first counted and their concentration adjusted to 10 million in 12 ml 10% FCS/PBS at room temperature and put into 15ml polypropylene tubes (used for cell culture and thus not excessively absorbing/fixing cells to the tube wall, Greiner Bio One). Then 650 µl of a 37 % formaldehyde PBS solution were added, i.e. an end-concentration of 1.9 % of formaldehyde was used for crosslinking/fixation, at room temperature for 10 min while gentle shaking to avoid cell aggregation. Note: the concentration of formaldehyde for crosslinking/fixation at this stage is ideal for the following steps and in respect to cell/nuclear integrity for the human and mouse cells we used here; although this might hold in general, cases are known where other concentrations and incubation times achieve better results. Thus, the tube was put on ice (from now on we kept everything on ice up to the 1st restriction of the DNA (see below) to avoid any damage of the material) and 1.6 ml of cold 1M Glycine in PBS were added to quench the crosslink/fixation. Thereafter, the cells were spun down for 8 min at 1300 rpm at 4°C, the resulting pellet was washed in ice-cold PBS, and taken up first in 1 ml before adding up to 14 ml of ice-cold PBS, followed again by spinning down for 8 min at 1300 rpm at 4°C. After discarding the supernatant, the pellet could now also be frozen for storage, but we advice to continue straight away with lysis and the 1st restriction. Again cells were spotted on glass slides to check for cellular and especially nuclear integrity by microscopy, with/without staining of the nucleus with DAPI (Note: again the cells could now also be used eventually for any other immunofluorescence or fluorescence in situ hybridization experiment).

*T2C Preparation of Cell Nuclei and 1st Nuclear Genomic DNA Restriction*

For cell lysis and preparation of cell nuclei, 5 ml of an always freshly prepared (for full activity) lysis buffer on ice (!) consisting of 10 mM Tris pH 8.0 (50 μl 1M), 10 mM NaCl (10 μl 5M), 0,2% NP-40 (100 μl 10%), 100 μl 50x complete Prot. Inhib. mix (50X = 1 tablet in 1 ml PBS), and filled up with up to 5 ml MilliQ (4.74 ml) was used. The pellet prepared in the final step of crosslinking/fixation was taken up in 1 ml of this lysis buffer, resuspended and filled up with another 4 ml to a total of 5 ml and incubated for 10 min on ice. The now free cell nuclei were spun down for 5 min at 1800 rpm at 4°C, the pellet was taken up in 0.5 ml of ice-cold PBS in a safe-lock tube, and spun for 1 min at 2600 rpm a at 4°C. Again it is possible after removal of the supernatant to snap-freeze, and store the nuclei at -80°C. For a check we always spotted nuclei on glass slides to check for nuclear integrity by microscopy and/or staining of the nucleus with DAPI.

For the 1st restriction the nuclei were now resuspended in 0.5 ml/tubes with 1.2x restriction buffer (60 μl restriction buffer, 440 μl MilliQ and adjusted for BSA if necessary) and transferred to a 1.5 ml safe-lock tube. Then to gently permeabilize the nuclear lamina the tubes were put at 37°C and 7.5 μl of 20% SDS (0.3% endconcentration) were added, and incubated at 37°C for 1 h, while shaking at 900 rpm. After adding 50 μl of Triton-X-100 (2% endconcentration) for further gentle permeabilzing of the nuclear lamina, the nuclei were again incubated at 37°C for 1 h, while shaking at 900 rpm. Note: both the SDS and Triton-X-100 step need to be carried out with great care to avoid any decrosslinking – again we checked that by checking the nuclei microscopically with and/or without DAPI staining. For future controls of the undigested material (the so called 1st unrestricted control) now a 5 μl aliquot was taken and stored at -20°C. Then 400 units of the selected restriction enzyme was added and incubated over night (~20 h) at 37°C. For the human cells in all cases the restriction enzyme BglII (Roche) was used. For the mouse cells we used either HindIII (Roche) or ApoI (New England Biolabs) was used. Note: even though its optimal temperature is 50°C, for ApoI 37°C should be used to prevent partial decrosslinking of the sample ;-). And again for future controls of the restriction now a 5 μl aliquot was taken and stored at -20°C (the so called 1st restriction control). After the 1st restriction 40 μl of 20% SDS (endconcentration 1.6%) was added to the remaining sample to stop the restriction and for further breakdown of the nuclear lamina by incubation at 65°C for 20-25 min, while being shaken at 900 rpm.

*T2C Dilution, Re-ligation and De-Crosslinking of Restricted Genomic DNA*

Thereafter, the fully digested nuclear material was diluted by transferal to a 50 ml falcon tube and addition of 6.125 ml 1.15x ligation buffer (6.125ml: 5.421 ml MilliQ water + 704 μl ligation buffer). Then 375 μl of 20% Triton-X-100 (endconcentration 1.0%) was added and incubated in a 37°C water bath for 1 h, while shaking every 10 min by hand. Then 20 μl Ligase HC 5U/μl (100 U in total, Roche) was added and incubated at 16°C over night (~20 h) followed by an additional 30 min of incubation at room temperature. To de-crosslink the non-ligated and ligated DNA 30 μl 10 mg/ml Proteinase K (in total 300 μg) was added and incubated at 65°C in a water bath over night (~20 h). Again for future controls of the relegation and de-crosslinking now a 5 μl aliquot was taken and stored at -20°C (so called re-ligation/de-crosslink control).

*T2C DNA Purification and 2nd (Re-ligated-)DNA Restriction/Sonication*

For further treatment of the sample, first the DNA was purified by adding 30 ml 10 mg/ml RNAse (300 µg in total) and incubating at 37°C for 30-45 min, followed by brief cooling to room temperature and addition of 7 ml phenol-chloroform and vigorous shaking. Then the sample was centrifuged at 4,000 rmp (2200xg) for 15 min, before the upper phase was put in a new 50 ml tube and 7 ml of MilliQ was added as well as 1 μl of glycogen per ml, 1.5 ml of 2M Sodium Acetate pH 5.6, and add 35 ml of 100% ethanol to enhance the purification, gently but thoroughly mixed and thereafter put at -80°C for 1.5-3 h. This was followed by direct centrifugation at 4,000 rmp (2200xg) for 15 min, supernatant removal, addition of 10 ml of 70% EtOH, resuspension, and again centrifugation at 4,000 rmp (2200xg) at 4°C for 15 min. After supernatant removal, the pellet was dried for 20 min and was dissoluted in 150 μl of 10 mM Tris pH 7.5 at 37°C for 30 min. Again for future controls of the relegation and de-crosslinking now a 5 μl aliquot was taken and stored at -20°C (so called 1st purification control).

Thereafter, the resulting re-ligated and de-crosslinked purified material was shortened by a 2nd restriction: First, to control the amount of DNA at this stage an aliquot of 1 μl was run alongside a reference sample of species-matched genomic DNA of known concentration on a 2% agarose gel. Then the DNA was adjusted in 0.5 ml/tubes to a 100 ng/µl concentration and restricted with the 2nd restriction enzyme by adding 1 U per µg of DNA of the selected restriction enzyme and incubated over night (~20 h) at 37°C. For the human cells in all cases the restriction enzyme NlaII (New England Biolabs) was used. For the mouse cells we used either if HindIII was used as 1st restriction enzyme DpnII (New England Biolabs) or if ApoI was used as 1st restriction enzyme sonication with 10 cycles of 15 sec on and 45 sec off. Again for future controls now a 5 μl aliquot was taken and stored at -20°C (so called 2nd restriction control – this can be combined with the purification control from above).

*T2C Treatment of the Various DNA Controls*

For controls of the integrity of the DNA at the different stages the following controls were used: i) 1st unrestricted control, ii) 1st restricted control, iii) re-ligation/de-crosslink control, iv) 1st purification control, and v) 2nd restriction/final purification control. These samples were controlled on a 2% agarose gel with corresponding plasmid DNA, which was restricted alongside, re-ligated and purified as external restriction control. For controls i)-iii) the aliquots were incubated with 10 µl Proteinase K (10 mg/ml) in 90 μl 10 mM Tris pH 7.5 at 65°C for at least 1 h. The DNA was purified by adding 3 µl 10 mg/ml RNAse and incubation for at 37°C for 30-45 min, followed by brief cooling to room temperature and addition of MilliQ up to 500 µl (~400 ml) as well as 500 µl phenol-chloroform and vigorous shaking. Then the controls were centrifuged at 13,200 rmp for 15 min, 2 µl of glycogen per ml, 50 µl of 2M Sodium Acetate pH 5.6, and add 850 µl of 100% EtOH were added, gently but thoroughly mixed and snap-frozen before direct procession to centrifugation at 13,200 rmp for 20 min, followed by supernatant removal, addition of 1 ml 70 EtOH, centrifugation at 13,200 rpm at 4°C, renewed supernatant removal, pellet drying for 20 min and dissolution in 20 µl of 10 mM Tris pH 7.5 at 37°C for 30 min.

*T2C General DNA Whole Genome Sequencing Library Preparation*

In general the *T2C* DNA fragment library was prepared for sequencing analysis on the Illumina Cluster Station and HiSeq 2000 Sequencer according to the Illumina TruSeq DNA protocol with *enhancing modifications from us* ([www.illumina.com](http://www.illumina.com), TruSeq DNA sample prep LS protocol; part#15026489 Rev. C): i) purification of the DNA fragments, ii) end-repair to reach blunt end status, iii) 3’-end adenylation to avoid chimera, iv) sequencing adapter ligation including eventual multiplexing step, and finally v) purification of the *T2C* whole genome sequencing DNA fragment library.

Therefore, first the concentration of the *T2C* DNA fragment library was measured again for fine tuning using 1 µl of material using Quant-it dsDNA broad range assay kit. Then the samples were split into 4 sets of 5 µg each of the *T2C* DNA fragment library and the following complete procedure done for each of these 4 sets of material:

i) To purify the *T2C* DNA library after the 2nd restriction AMPure XP beads (Beckman Coulter) were used by adding 1.8 µl AMPure XP beads per 1.0 µl of digested DNA. This was incubated at room temperature for 5 minutes, placed on the magnetic stand and incubated at room temperature for 5 minutes, and the supernatant was discarded without disturbing the beads. The beads were washed 2 times with freshly prepared 70% ethanol, placed at 37°C for 5 minutes to let the beads dry. Then the beads were resuspended in 50 µl PCR grade water and incubated at room temperature for 5 minutes, placed on the magnetic stand for 5 minutes, and finally 50 µl supernatant was transferred to a new tube. One microliter was finally loaded on an Agilent Technologies 2100 bioanalyzer using a DNA 1000 assay to determine the quality of the purified digested DNA.

ii) For end-repair of the *T2C* library, DNA fragments, since they were restricted or sonicated before with overhanging ends, 4 material sets were each in 50 µl transferred to a 96 well plate. Since no in-line control reagent to avoid contamination of the material was used, 10 µl of resuspension buffer were added, followed by 40 µl of end repair mix, and mixed thoroughly but gently pipetting the entire volume up and down 10 times. Then the plate was covered with a Microseal ‘B’ adhesive seal and placed on the pre-heated thermal cycler at 30°C for 30 min. After removing the adhesive seal from the plate, first the AMPure XP beads were vortexed until they were well dispersed, and 160 µl (consisting of 136 µl of AMPure XP beads mixed with 24 µl of PCR grade water) were added to the wells and the entire volume was again pipetted thoroughly but gently up and down 10 times. After 15 min of incubation, the plate was put on the magnetic stand at room temperature for another 15 min until the liquid appeared clear. Then twice 127.5 µl of the supernatant was removed, and thereafter 200 µl of freshly prepared 80 % EtOH was filled into the well of the plate without disturbing the beads, incubated at room temperature for 30 sec and discarded again without disturbing the beads. This was repeated twice before drying of the plate for 15 min. Only thereafter the plate was removed from the magnetic stand and the pellets resuspended with 17.5 µl of resuspension buffer, followed by 10 times thorough but gentle mixing by pipetting 10 times up and down. After incubation at room temperature for 2 min, the plate was put back on the magnetic stand at room temperature for 5 min again until the liquid appeared clear, and then 15 µl of the clear supernatant was removed containing the end-repaired material ready for the Adenylation of the 3’-ends in the next step.

iii) For 3’-end adenylation of the end-repaired *T2C* DNA fragment libraries, i.e. to prevent the blunt ends from ligating to one another, and thus to ensure a low rate of chimera (concatenated template) formation during the adapter ligation reaction in step iv), Klenow exo enzyme in the presence of ATP was used. A corresponding single ‘T’ nucleotide on the 3’ end of the adapter provided a complementary overhang for ligating the adapter to the fragment.

Therefore, 15 µl of the end-repaired *T2C* DNA fragment library were transferred to a new 0.3 ml PCR plate. Since the in-line control reagent to avoid contamination of the material was again not used 2.5 µl of the resuspension buffer was added, followed by 12.5 µl of thawed A-tailing mix, pipetted thoroughly but gently up and down 10 times. Then the plate was sealed with a Microseal ‘B’ adhesive seal, and the plate was placed on a pre-heated thermal cycler at 37°C for 30 min. Immediately after removal of the plate from the thermal cycler, the adapter ligation took place.

iv) To ligate the sequencing adaptors using the Illumina provided indexed adapters #6 and #12, DNA adapter tubes and stop ligation buffer tubes were used, and centrifuged to 600 xg for 5 seconds. Immediately before use, the ligation mix containing tube was removed from the -25°C storage as recommended by Illumina. Since the in-line control reagent to avoid contamination of the material was again not used 2.5 µl of the resuspension buffer was added to the wells of another PCR plate, and 2.5 µl of the ligation mix was added as well. Then 2.5 µl from the appropriate adaptor tubes was added and thoroughly but gently pipetted up and down 10 times. Then the plate was sealed again with a Microseal ‘B’ adhesive seal and the plate centrifuged to 280xg for 1 min. Thereafter, the plate was incubated on a pre-heated thermal cycler at 30°C for 10 min, the plate was taken down from the cycler, the adhesive seal removed, 5 µl of the stop ligation buffer was added, and thoroughly but gently pipetted up and down 10 times.

v) To purify the sequencer adapted *T2C* DNA fragment libraries again AMPure XP beads were used. Therefore, AMPure XP Beads were centrifuged until they were well dispersed and 42.5 μl of mixed AMPure XP Beads were added to the wells and thoroughly but gently pipetted up and down 10 times, before incubation at room temperature for 15 min. Then the plate was placed on the magnetic stand at room temperature for minimum 5 min or longer until the liquid appeared clear. Then 80 μl of the supernatant were removed from each well of the plate and while the plate remained on the magnetic stand, 200 μl of freshly prepared 80% EtOH were added without disturbing the beads, and incubated at room temperature for 30 sec. The complete supernatant was then removed. This EtOH wash was done twice, before the still on the magnetic stand resting plate was air-dried at room temperature for 15 min. After removal from the magnetic stand, the dried pellet was resuspended using 52.5 µl of resuspension buffer, and thoroughly but gently pipetted up and down 10 times. After incubation for 2 min, the plate was put back to the magnetic stand at room temperature for minimum 5 min or longer until the liquid appeared clear. Then 50 µl of the clear supernatant was transferred to a new 0.3. PCR plate for a second cleanup, and 50 µl of vortexed AMPure XP beads added, and thoroughly but gently pipetted up and down 10 times. Then the plate was again incubated at room temperature for 15 min, the plate was placed again on the magnetic stand at room temperature for minimum 5 min or longer until the liquid appeared clear. 95 µl of the supernatant were removed, while the plate still remained on the magnetic stand, 200 μl of freshly prepared 80% EtOH was added to each well without disturbing the beads, incubated at room temperature for 30 sec. The complete supernatant was then removed. This EtOH wash was done again twice, before the still on the magnetic stand resting plate was air-dried at room temperature for 15 min. After removal from the magnetic stand, the dried pellet was resuspended using 22.5 µl of resuspension buffer, and thoroughly but gently pipetted up and down 10 times. Again after incubation for 2 min the plate was put back to the magnetic stand at room temperature for minimum 5 min or longer until the liquid appeared clear. Finally 20 μl of the clear supernatant from each well of the plate were collected and the material from each of the 4 in parallel treated *T2C* DNA fragment libraries splits pooled.

*T2C Regional DNA Sequencing Capture Microarray Design*

To achieve high-resolution and allow for high-throughput multiplexed sequencing and thus to achieve a highly relevant local interaction mapping, i.e. to achieve a high quality *T2C*, special capture arrays were designed to select specifically for genome regions of interest avoiding sequencing of unnecessary background, i.e. to create a region specific DNA sequencing library optimized for selection of the re-ligated DNA pieces after the 1st restriction, i.e. directly for interactions only in specific and relatively small genomic regions. Therefore, in a close cooperation with NimbleGen, we designed DNA oligos for 2.1M capture microarrays, i.e. capture microarrays capable of in principle fishing 2.1 million different genomic sequences with the same amount of different oligos. To achieve a real high quality result *T2C* only (!) one oligo was placed up- and one downstream as near as possible to the 1st restriction site used in the nuclear whole genome restriction, since the interest lies in sequencing just each side after re-ligation of this 1st restriction. The oligos were designed by NimbleGen and us for the selected regions of the human and mouse genomes using genome builts mm9 and HG19 with oligo length of 72±3 bp, unique appearance (no mismatch allowed) in the entire genome, and with respect to best and similar capturing, i.e. similar hybridization,on a microarray. Then the oligos were further selected: in the case of using a 2nd restriction enzyme to shorten the re-ligated DNA library for sequencing the oligo had to be situated between the 1st and 2nd restriction site. In the case of using sonication to shorten the re-ligated DNA only oligos within 150 bp of the 1st restriction site were chosen. If only one oligo was present, which crossed either the 1st or 2nd or even both restriction sites, only cuts at the oligo beginning or end of in total not more than 10 % were allowed, i.e. that the oligos could definitely capture DNA pieces with a minimum of 65 bp to guaranty specificity and similar hybridization efficiency. The same condition was applied in the sonication case, for the 1st restriction side. Thereafter, we mapped the oligos on the genome and controlled by hand whether the conditions were fulfilled and whether the oligos were properly placed in respect to other genome features. For production of the microarray the number of the 2.1 million possible different oligos $O_{array}$ was divided by the number of selected oligos $O_{selected}$ and then each selected oligo spotted $N_{spotted}$times, with $N_{spotted}=Abs (\frac{O_{array}}{O_{selected}})$, on the actual capturing array during the production process by NimbleGen. Thus, with the number of oligos used for capturing (see below) using a 1st and 2nd restriction enzyme we can be sure to have ~10^10^ oligo molecules for each different oligo on the microarray, and thus with the 10^7^ cells we use as input, we are fare away from saturating the array by a factor of > 10^5^ to 10^6^. In the case of the experiment using sonication with ~250 times more oligos and in total ~50 times more genomic regions covered that is still > 10^2^, if considering the losses in the experimental procedures up to the capture array.

Concerning the experiments using a 1st and 2nd restriction enzymes, the balance between the region size chosen, the resulting size of the interaction matrix, i.e. the all possible interactions between all restriction fragments within this region, and the sequencing capabilities to achieve a high frequency range of a minimum of 4 to 5 orders of magnitude for each possible interaction (we assume an average of 2 to 3 orders of magnitude, which results in a spread of 4 to 5 orders of magnitude) was calculated. Thus, for a sequencing capability in two sequencing lanes of ~300 and 500 million sequences, i.e. 300 and 500 million sequencings of possible interaction events, with the aim of achieving on average of 100 to 1,000 sequencing events per interaction 500 to 1,000 oligos and thus interaction fragments are optimal. The genomic region then covered depends only on the resolution, i.e. average spacing of the 1st restriction enzyme within the genome.

In the case of a 1st and 2nd restriction enzyme we chose the oligos and capture arrays as follows: In the human case this was done for the H19/IGF2 region on chromosome 11 from base pair position ~1,110,650 to ~3,216,350, i.e. a 2,105,700 bp sized region and 525 oligos. In the mouse case this was done for the β-globin region on chromosome 7 from base pair position ~109,876,350 to 111,966,600, i.e. a 2,090,250 bp sized region and 800 oligos.

*T2C Regional DNA Sequencing Library Preparation - Microarray Capturing*

To produce a subselected regional *T2C* DNA fragment sequencing library from the *T2C* whole genome DNA fragment sequencing library, the pooled DNA library after ligation of the sequencing adapters was subject to subselection with the above described newly and specifically developed capturing microarrays using the NimbleGen Array capture protocol and hybridization system with *enhancing modifications from* ([www.nimblegen.com/seqcapez](http://www.nimblegen.com/seqcapez), NimbleGen Arrays User’s Guide, Sequence Capture Array Delivery version 3.2): The entire procedure consisted of i) microarray hybridization, ii) washing before iii) elution of the captured regional DNA library from the microarray.

i) Therefore, 3 h before the capturing, the hybridization system was set to 42°C, a first heat block was set to 95°C, and another one to 70°C, to equilibrate. Then the hybridization mixture was prepaired by adding 300 µl of 1 mg/ml Cot-1 DNA to the pooled DNA library after ligation of the sequencing adapters. In the case of using multiplexed samples not only the 4 sets of material were pooled but also the multiplexed samples were pooled. This saves microarray capacity and since the amount of DNA to be captured is far from the saturation of the microarray this leaves room for multiplexing up to 10 to 100 samples depending on the DNA amount, concentrations, and method to be used. Here multiplexing was only down by pooling 2 different materials. Then the sample was dried in a SpeedVac at 60°C for around 30 to 45 min, 11.2 µl of VWR water was added for rehydration, vortexed and centrifuged at maximum speed for 30 sec, before placement on the 70°C heat block for 10 min to fully solubilize the DNA. After a second vortexing and again centrifugation at maximum speed for 30 sec, 18.5 µl of 2X SC hybridization buffer and SC hybridization component A are added, followed again by vortexing and again centrifugation at maximum speed for 30 sec. Then to denature the DNA the sample was placed on the 95°C heat block for 10 min before another centrifugation at maximum speed for 30 sec. Thereafter, the sample was placed at 42°C and from there immediately loaded on the microarray hybridization chamber (the complete microarray system was prepared in parallel) and hybridized at 42°C for 64 h.

ii) To wash the captured regional *T2C* DNA library on the microarray first the elution chamber was assembled according to the NimbleGen array user guide. Therefore, the microarray slide was removed from the 42°C NimbleGene hybridization system and placed directly into the disassembly basin containing 100 ml of SC wash buffer II heated to 47.5°C. After ~10 sec used for equilibration the mixer was peeled of and the slide was transferred to a second wash tube containing SC wash buffer II at 47.5°C, the sealed wash tube was inverted 10 times at a rate of 1 inversion per second. Then the slide was transferred to a new wash tube containing 32 ml of stringent wash buffer at 47.5°C, and the sealed tube was inverted 10 times at a rate of 1 inversion per second, before resting at 47.5°C for 5 min, and again inverted 10 times at a rate of 1 inversion per second. Then the slide was again transferred to a new tube containing 32 ml of stringent wash buffer at 47.5°C, and the closed tube was inverted 10 times at a rate of 1 inversion per second, before resting at 47.5°C for 5 min, and again inverted 10 times at a rate of 1 inversion per second. Then the slide was again transferred to a new tube containing 32ml of SC wash buffer I at room temperature, and the closed tube was inverted at a rate of 1 inversion per second for 2 min. Then the slide was again transferred to a new tube containing 32 ml of SC wash buffer II at room temperature, and the closed tube was inverted at a rate of 1 inversion per second for 1 min. Then the slide was again transferred to a new tube containing 32 ml of SC wash buffer III at room temperature, and the closed tube was inverted 10 times at a rate of 1 inversion per second.

iii) To elute the captured regional *T2C* DNA fragment sequencing library from the microarray the slide was transferred to the NimbleGen EL1 elution system at room temperature. Then ~900 µl of 125 mM NaOH were added to the elution chamber until it is full, and incubated for 10 min. The eluted regional *T2C* DNA fragment sequencing library was pipetted to a 1.5 ml tube and filled up to 900 µl of 125 mM NaOH, followed by division equally in two new tubes containing 516 µl of a well mixed solution of 16 µl 20% acetic acid solution and 500 µl Qiagen Buffer PBI prepared beforehand in a 1.5ml tube. Then the mixture was transferred to a single MinElute column on a centrifuge to draw the solution through the column in several steps of 700 µl each. Then 750 µl buffer PE was put the column and centrifuged through. Then the MinElute column was put into a 2ml collection tube and centrifuged at maximum speed for 1 min. to remove any residual buffer PE. The flow-through was discarded, before placement of the MinElute column in a clean 1.5ml tube, 25 µl of buffer EB was added to the column, incubated for 1 min, and centrifuge at maximum speed for 1 min.

*T2C Amplification, Cluster Generation, and Paired-end High-Throughput Sequencing*

First for paired-end sequencing the *T2C* regional DNA fragment sequencing library was enriched for sequencing first by PCR using Fusion polymerase using 30 sec at 98°C, 12 cycles of (10 sec at 98°C, 30 sec at 60°C, 30 sec at 72°C), 5 min at 72C final extension. For each 1 µg of *T2C* regional DNA fragment library 5 µl of the PCR primer cocktail and 25 µl PCR master mix was added to the PCR plate. For purification AMPure XP beads (Beckman Coulter) were used by adding 1.8 µl AMPure XP beads per 1.0 µl of DNA. This was incubated at room temperature for 5 minutes, placed on the magnetic stand and incubated at room temperature for 5 minutes, and the supernatant was discarded without disturbing the beads. The beads were washed 2 times with freshly prepared 70% ethanol, placed at 37°C for 5 minutes to let the beads dry. Then the beads were resuspended in 30 µl resuspension buffer and incubated at room temperature for 5 minutes, placed on the magnetic stand for 5 minutes, and finally 50 µl supernatant was transferred to a new tube. One microliter was finally loaded on an Agilent Technologies 2100 Bioanalyzer using a DNA 1000 assay to determine the quality of the purified digested DNA.

Cluster generation was performed according to the Illumina cBot User Guide ([www.illumina.com](http://www.illumina.com), part#15006165 RevE). Briefly, 1 μl of a 10 nM TruSeq DNA library stock DNA was denatured with NaOH, diluted to 10 pM and hybridized onto the flowcell. The hybridized fragments are sequentially amplified, linearized and end-blocked according to the Illumina Paired-end Sequencing user guide protocol. After hybridization of the sequencing primer, sequencing-by-synthesis was performed using the HiSeq 2000 sequencer with a 101 cycle protocol according to the instructions of the manufacturer. The sequenced fragments were denaturated with NaOH using the HiSeq 2000 and the index-primer was hybridized onto the fragments. The index was sequenced with a 7-cycle protocol. The fragments were denaturated with NAOH, sequentially amplified, linearized and end-blocked. After hybridization of the sequencing primer, sequencing-by-synthesis of the third read was performed using the HiSeq 2000 sequencer with a 101-cycle protocol.

*T2C Sequence Mapping and Classification*

The raw sequence reads were checked for the existence of the first restriction enzyme recognition sequence in the sequencing direction. The sequence after the first enzyme recognition site was removed. If the bases of the recognition site after the overhang were not unambiguously, the read was further trimmed by removing all the bases after the end of the overhang. These trimmed sequences were aligned using the Burrows-Wheeler Alignment (BWA) tool [S7] to the whole human genome NCBI36/hg18 assembly and to the mouse NCBI37/mm9 assembly. Therefore the following default parameter set was used (with the value of the parameter in [] brackets):

bwa aln [options] <prefix> <in.fq>

-n NUM max #diff (int) or missing prob under 0.02 err rate (float) [0.04]

-o INT maximum number or fraction of gap opens [1]

-e INT maximum number of gap extensions, -1 for disabling long gaps [-1]

-i INT do not put an indel within INT bp towards the ends [5]

-d INT maximum occurrences for extending a long deletion [10]

-l INT seed length [32]

-k INT maximum differences in the seed [2]

-m INT maximum entries in the queue [2000000]

-t INT number of threads [1]

-M INT mismatch penalty [3]

-O INT gap open penalty [11]

-E INT gap extension penalty [4]

-R INT stop searching when there are >INT equally best hits [30]

-q INT quality threshold for read trimming down to 35bp [0]

-f FILE file to write output to instead of stdout

-B INT length of barcode

-L log-scaled gap penalty for long deletions

-N non-iterative mode: search for all n-difference hits (slooow)

-I the input is in the Illumina 1.3+ FASTQ-like format

-b the input read file is in the BAM format

-0 use single-end reads only (effective with -b)

-1 use the 1st read in a pair (effective with -b)

-2 use the 2nd read in a pair (effective with -b)

-Y filter Casava-filtered sequences

In case of using a second restriction enzyme (and thus not in the case of sonication) the unique sequences were aligned in a second step to a masked genome, excluding the sequence parts between second restriction enzymes that did not contain a first enzyme recognition site. Finally, only those sequences were paired using the SAMtools [S8] to generate paired-end Binary Alignment/Map (BAM) files, which showed in both the whole and masked genome reference sequences a unique alignment. Note: the alignments are unique, but nevertheless contain mismatches etc., which are either do to sequencing errors or hint a difference of our cells/mice to the reference genome. Unfortunately, there is also no way of distinguishing false positive or false negative alignments. Consequently, the resulting paired-end sequences then contain the interaction information with an error rate determined by the error rate of sequencing, the quality of the reference sequence, and the difference the DNA sequence of our cells/mice to this reference genome. A rough estimate of the false positive and false negative results for unique sequences without mismatches at the end of this process using known error rates indicate the error to be smaller than 10^4^ after accumulation of errors and the reduction of errors due to our procedure. This can be also deducted from the reduction of sequence pairs from the initial raw sequence throughout the entire process to the final result. Besides, omitting neighbouring interaction fragment pairs, leads to “secured” data sets where we can be sure that the result is not based on a unresctricted or restricted followed by re-ligation event.

*Computer Simulations*

Using a two-step combined Monte Carlo and Brownian Dynamics method, the Random-Walk/Giant-Loop (RW/GL) model [56] and the Multi Loop Subcompartment (MLS) model [3,5,7,8,59,87,88] were simulated for human interphase chromosome 15. Assuming a flexible polymer chain, the chromatin fibre was split into ~3,300 segments of 300 nm (~31 kbp). To each segment a harmonic stretching potential and between two segments a harmonic bending potential were assigned. To avoid self-crossing of the polymer chain, a short ranged excluded volume potential was introduced, whose potential barrier could be changed to facilitate chain dis-entanglement to speed up simulations. *In vivo* this is mediated by Topoisomerase-II especially during chromosome de- or condensation. The simulation of single chromosomes necessitated placement into an embedding potential simulating the surrounding nuclear chromosomes. Different models were simulated (Table S7): In the RW/GL model loops of 5.0, 4.0, 3.0, 2.0, 1.0, 0.504 and 0.252 Mbp were used and connected by a chromatin linker whose length was adjusted such that the global territory behaviour yielded comparable results as in the MLS model. In the MLS model the loop size was 126 kbp with linker sizes of 63, 126, 189 and 252 kb. The number of loops in the rosettes varied, since the DNA content of the rosettes was assumed to be that of the metaphase ideogram banding pattern [S1] divided by three to account for the transition into interphase ideogram bands [S2]. The starting configuration of a chromosome had the approximate form and size of a metaphase-chromosome whose de-condensation resembles the natural process. Typically ~400,000 Monte Carlo steps were needed to generate enough statistically independent configurations at thermodynamic equilibrium. For comparison with experimental spatial distance measurements between genetic markers as a function of their genetic separation, and the here interesting spatial distance and interaction maps, 100 to 150 statistically independent Monte Carlo configurations were taken as starting points for relaxation at higher spatial resolution by Brownian Dynamics methods using a decreased segment length of 50 nm (~5.2 kb), corresponding to 20,000 segments for chromosome 15. 2,000 Brownian Dynamics steps were performed until equilibration was reached again. To simulate the dynamics for illustration purposes, as e.g. for the decondensation from a metaphase starting configuration into interphase, the resolution was set right away to the decreased segment length of 50 nm (~5.2 kb; Movie S1) or 20 nm (~2.0 kbp; Movie S2-4) and only Brownian Dynamics steps of 10 ns were performed.

*Simulated Spatial Distances, Spatial Distance and Interaction Maps*

The simulated spatial distances were determined position dependently, i.e. the marker pairs were placed in respect to the topological folding of the simulated chromosome and position independently, i.e. the pairs of markers were placed randomly and therefore regardless of any folding topology on the chromosome (for the underlying assumption see results). All pairs of positions for one genomic separation were taken, the resulting spatial distance determined and averaged over 100 to 150 statistically independent chromosome configurations. For two-dimensional spatial distance and interaction maps the segments were divided by a factor of 10, i.e. a resolution of ~520bp. For two-dimensional spatial distance maps all distances were calculated between every combination of subdivided segments, i.e. genetic positions, and again averaged for each distance matrix element over 100 to 150 statistically independent chromosome configurations. For two-dimensional interaction maps for each of the subdivided segments all interactions within radii of 30 nm, 40 nm, 50 nm, 60 nm, 70 nm, 80 nm, 90 nm, 100 nm, 110 nm, 120 nm, 130 nm, 140 nm, and 150 nm, respectively where calculated and cumulated into the corresponding interaction matrix, while averaging over 100 to 150 statistically independent chromosome configurations. Notably, the diagonal of the interaction matrix, due to the radii in relation to the segment resolution acts as an intrinsic normalization.

*Scaling Properties of Computer Simulated Genome Architectures*

In order to describe quantitatively the different chromatin topologies and microscopic morphologies resulting from the Multi-Loop-Subcompartment (MLS) or the Random-Walk/Giant-Loop (RW/GL) models of simulated chromosomes and simulated nuclei, their scaling behaviour was investigated (Figure S11-13; and [3,5,15,16,59]). The scaling behaviour describes how a parameter, e.g. the length of the coast of Britain, depends e.g. on the scale of observation, i.e. the length of the ruler used to measure the coastline. Since the coastline is self-similar it follows a power-law. The exponent of the power-law is the scaling or fractal dimension of the coastline. For Britain it values ~1.24. Mathematical lines, surfaces and volumes have fractal dimensions of 1.0, 2.0 and 3.0, which agree with their Euklidian dimension. Thus, the British coast scales like a folded and not a straight line. To determine the scaling behaviour of different chromatin topologies the exact spatial distance and exact yard stick dimensions were determined:

The scaling behaviour of the one-dimensional axis of the simulated 30 nm chromatin fibre was investigated by the exact spatial-distance dimension $D_{SD}$and the exact yard-stick $D_{Y}$ dimension. Neglecting the different measuring process $D_{SD}<=> D_{Y}$ in the limit [S9]. $D_{SD}$ is the inverse exponent in the scaling relation between the distance ${R_{SD}(c}_{SD})$ connecting two chain positions and the contour length $c_{SD}$ e. g. in bp

$R_{SD}(c_{SD})\sim c_{SD}^{(1/D_{SD})}$. (Equation 1)

This corresponds to the measurement of spatial distances between genomic markers as function of their genomic separation $c_{SD}$. Since $R_{SD}$ depends for large $c_{SD}$on the marker position, the average over several randomly placed marker pairs need to be taken. Genomic separations ≥ 5.2 kbp (the base pair content of one chain segment) up to the whole chromosome were used. All possible maker pairs for genomic separations < 25 Mbp and 5000 pairs > 25 Mbp were taken. The average was taken over 100 to 150 chromosome configurations.

The exact yard-stick dimension $D_{Y}$ is the negative exponent in the scaling relation between the curve length ${C_{Y}(l}_{Y})$ and the yard-stick length $l_{Y}$

$C_{Y}\left( l_{Y} \right)=N_{Y}\cdot l_{Y}\sim l_{Y}^{\left( 1-D_{Y} \right)}$and $N(l_{y})\sim l_{Y}^{{-D}_{Y}}$ (Equation 2)

with the number of yard-sticks $N_{Y}$, depending itself on $l_{Y}$. The beginning coordinates of a randomly chosen chain segment defined the initial start point from which the necessary $N_{Y}$ to reach both chain ends was calculated by walking along the chain with $l_{Y}$ sized steps. First the distance to the next segments was determined until it surpassed $l_{Y}$(Figure S11). Hence, the exact chain position and the start point of the next step where the distance equals $l_{Y}$ is a coordinate point within the previous segment. This is given by the corresponding trigonometric vector equation. Near the chain end and/or for big $l_{Y}$, the distance could be > $l_{Y}$, thus $N_{Y}$ was determined by the fraction of $l_{Y}$ to reach the chain end. The resolution of $l_{Y}$ was 5, 25 and 50 nm up to scaling length of 10^2^, 10^3^ and 10^4^, respectively. Since, $N_{Y}$ depends on the initial start point the bigger $l_{Y}$, $S\left( l_{Y} \right)=abs(0.05l_{Y})$ ($l_{Y}$ in nm) start points were averaged, thus the standard error of $D_{Y}$ was always <0.01. The mean was taken over 100 to 150 statistical chromosome configurations.

## Scaling Analysis Genomic Interactions

The analysis of the scaling properties of the interaction frequency $I(s)$ as function of the genetic separation $s$ in base pairs is based on the sum and thus average of all possible interactions $k$ whose genetic separations between two genetic chromosomal base pair locations $p_{i}$ and $p_{j}$ (whith $p_{j}$ > $p_{i}$) are within a window/bin of size $w$ centered around s and thus in the interval $\left[ s-\frac{1}{2}w,s+\frac{1}{2}w \right[$ and thus:

$I\left( s \right)=I(s_{w})= \sum_{0}^{k} s_{k}=\sum_{0}^{k} {(p_{j}-p_{i})}_{k}$ . (Equation 3)

For a fractal self-similar sequence like a random-walk the interaction scaling function $I(s)$ shows power-law behaviour:

$I\left( s \right)\sim s^{\iota}$ with $-1.0\leq\iota\leq0.0$ (Equation 4)

where -1.0 characterizes a negatively, -0.5 a randomly and 0.0 a positively correlated sequence.

To determine the local interaction coefficient for the analysis of the general behaviour and fine-structural features of long-range interaction scaling as a function of the genetic separation , the following asymmetric finite difference quotient of second order can be applied to with :

$\iota\left( s_{i} \right)=\frac{k}{h(h+k)}\tilde{I}\left( \tilde{s}_{i}+h \right)-\frac{k-h}{hk}\tilde{I}\left( \tilde{s}_{i} \right)-\frac{h}{k\left( h+k \right)}\tilde{I}\left( \tilde{s}_{i}-k \right)$ (Equation 5)

with

$k=\tilde{s}_{i}-\tilde{s}_{i-1}=\log s_{i}-\log s_{i-1}$ (Equation 6)

$h=\tilde{s}_{i+1}-\tilde{s}_{i}=\log s_{i+1}-{\log s}_{i}$ (Equation 7)

$\tilde{I}\left( \tilde{s}_{i}-k \right)=\log I\left( s_{i-1} \right)-I_{i-1}$ (Equation 8)

$\tilde{I}\left( \tilde{s}_{i} \right)=\log I(s_{i})=I_{i}$ (Equation 9)

$\tilde{I}\left( \tilde{s}_{i}+h \right)=\log I\left( s_{i+1} \right)=I_{i+1}$ (Equation 10)

## Correlation Analysis of DNA Sequences of Genomes

The analysis of long-range power-law correlations in genetic sequences here was done as described in earlier [5,15,16,40]. Briefly: It is based on the concentration profile of single nucleotides along the DNA sequence: The square root of the mean-square deviation between the concentration of nucleotides in a window of length and the concentration of nucleotides in the entire DNA sequence with length was calculated

(Equation 11)

while averaging over all possible window positions. Nucleotides used were adenine (A), thymine (T), guanine (G), and cytosine (C) as well as their grouping into purines (A+G) and pyrimidines (T+C). “Unknown” nucleotides were accounted for by using their general appearance probabilities. Since purines/pyrimidines are complimentary, the results are equal and their analysis as base versus non-base equals mapping the DNA sequence to the trajectory of a one-dimensional random-walk. In the following, only the results for purines vs. pyrimidines are considered.

For a fractal self-similar sequence like a random-walk the concentration fluctuation function shows power-law behaviour:

with (Equation 12)

where -1.0 characterizes a negatively, -0.5 a randomly and 0.0 a positively correlated sequence. The power-law behaviour of is connected to the power-law behaviour of the minimum and maximum deviation function [S10], the common autocorrelation function , and the power spectrum with frequency via

(Equation 13)

[S11-S14]. is related to the common autocorrelation function by double summation

(Equation 14)

Thus, local random fluctuations are substantially reduced and the analysis leads to a more reliable characterization of the DNA sequence compared to e.g. [S10,S15,S16]. Numerical calculation of by using Eq. 1 in this sequence of operations

(Equation 15)

by means of the probabilities for a nucleotide at a certain position , and e.g. for purines and elsewhere, leads to extreme numerical instabilities [5,15,16]. These instabilities were avoided by expansion to

(Equation 16)

and by exact calculation provided by the GNU multiple precision package GMP. The greater stability is due to the start of deviations from the exact result and becomes especially important for sequences longer than 10^5^ base pairs. To save computer power, the program adjusted automatically the precision (guaranteeing > 8 digits) depending on the sequence length.

To determine the local correlation coefficient for the analysis of the general behaviour and fine-structural features of long-range correlations as a function of the window size , the following asymmetric finite difference quotient of second order was applied to with :

(Equation 17)

with

(Equation 18)

(Equation 19)

(Equation 20)

(Equation 21)

(Equation 22)

To reduce the enormous computer power needed to calculate and for every possible , every from 1 to 10^4^ bp and only 900 logarithmically distributed for every order of magnitude thereafter were chosen. Calculations were performed on our world wide Correlizer@home BOINC grid (http://svahesrv2.bioquant.uni-heidelberg.de/correlizer/), the German D-Grid, the European Grid Initiative EGEE, as well as the Erasmus Computing Grid), the Almere Grid, and all the unnamed computing grids we have access through via these. Therefore the analyses were split into jobs of a few minutes computing a small number of windows each, thus being an extremely efficient ‘gap-filler’ on all these machines.

*Random sequences:* To investigate the error behaviour and to determine the origin of various correlation properties, artificial sequences based on different assumptions about their composition were constructed: They were constructed from a uniform distribution of base pairs using a R250 random number generator based on 16 parallel copies of a linear shift register with a period of [S17]. This is a far greater period compared to the linear congruent generator used normally and thus produces series with no structure resulting from the random number generator. The R250 generator is computationally faster as well [S18]. The base pair composition was biased by the human base pair distribution (A: 30 %, C: 20 %, G: 20 %, T: 30 %). Other biases were not chosen here, since a simple base pair bias does not result in different general, multi-scaling or fine-structure correlation behaviours (for further details and more complicated random sequences see [5,16,40]).

***Supplemental References:***

1. Francke U: Digitized and differentially shaded human chromosome ideograms for genomic applications. *Cytogenet Cell Genet* 65 1994:206-219.
2. Yunis JJ: Mid-prophase human chromosomes. The attainment of 2000 bands. *Hum Genet* 56 1981:293-298.
3. Bartek J, Bartkova J, Kyprianou N, Lalani EN, Staskova Z, Shearer M, Chang S, Taylor-Papadimitriou J: Efficient immortalization of luminal epithelial cells from human mammary gland by introduction of simian virus 40 large tumor antigen with a recombinant retrovirus. *Proc Natl Acad Sci* *USA* 88 1991:3520-3524.
4. Nativio R, Wendt KS, Ito Y, Huddleston, JE, Uribe-Lewis S, Woodfine K, Krueger C, Reik W, Peters JM, Murell A: Cohesin is required for higher-order chromatin conformation at the imprinted IGF2-H19 locus. *PLoS Genet* 5(11) 2009:e1000739.
5. [Bornkamm GW](http://www.ncbi.nlm.nih.gov/pubmed?term=Bornkamm%20GW%5BAuthor%5D&cauthor=true&cauthor_uid=16147984), [Berens C](http://www.ncbi.nlm.nih.gov/pubmed?term=Berens%20C%5BAuthor%5D&cauthor=true&cauthor_uid=16147984), [Kuklik-Roos C](http://www.ncbi.nlm.nih.gov/pubmed?term=Kuklik-Roos%20C%5BAuthor%5D&cauthor=true&cauthor_uid=16147984), [Bechet JM](http://www.ncbi.nlm.nih.gov/pubmed?term=Bechet%20JM%5BAuthor%5D&cauthor=true&cauthor_uid=16147984), [Laux G](http://www.ncbi.nlm.nih.gov/pubmed?term=Laux%20G%5BAuthor%5D&cauthor=true&cauthor_uid=16147984), [Bachl J](http://www.ncbi.nlm.nih.gov/pubmed?term=Bachl%20J%5BAuthor%5D&cauthor=true&cauthor_uid=16147984), [Korndoerfer M](http://www.ncbi.nlm.nih.gov/pubmed?term=Korndoerfer%20M%5BAuthor%5D&cauthor=true&cauthor_uid=16147984), [Schlee M](http://www.ncbi.nlm.nih.gov/pubmed?term=Schlee%20M%5BAuthor%5D&cauthor=true&cauthor_uid=16147984), [Hölzel M](http://www.ncbi.nlm.nih.gov/pubmed?term=H%C3%B6lzel%20M%5BAuthor%5D&cauthor=true&cauthor_uid=16147984), [Malamoussi A](http://www.ncbi.nlm.nih.gov/pubmed?term=Malamoussi%20A%5BAuthor%5D&cauthor=true&cauthor_uid=16147984), [Chapman RD](http://www.ncbi.nlm.nih.gov/pubmed?term=Chapman%20RD%5BAuthor%5D&cauthor=true&cauthor_uid=16147984), [Nimmerjahn F](http://www.ncbi.nlm.nih.gov/pubmed?term=Nimmerjahn%20F%5BAuthor%5D&cauthor=true&cauthor_uid=16147984), [Mautner J](http://www.ncbi.nlm.nih.gov/pubmed?term=Mautner%20J%5BAuthor%5D&cauthor=true&cauthor_uid=16147984), [Hillen W](http://www.ncbi.nlm.nih.gov/pubmed?term=Hillen%20W%5BAuthor%5D&cauthor=true&cauthor_uid=16147984), [Bujard H](http://www.ncbi.nlm.nih.gov/pubmed?term=Bujard%20H%5BAuthor%5D&cauthor=true&cauthor_uid=16147984), [Feuillard J](http://www.ncbi.nlm.nih.gov/pubmed?term=Feuillard%20J%5BAuthor%5D&cauthor=true&cauthor_uid=16147984): Stringent doxycycline-dependent control of gene activities using an episomal one-vector system. *Nucleic Acids Res* 33(16) 2005:e137.
6. Schockel L, Mockel M, Mayer B, Boos D, Stemmann O: Cleavage of cohesin rings coordinates the separation of centrioles and chromatids. *Nat Cell Biol* 13(8) 2011:966-972.
7. Li H, Handsaker B, Wysoker A, Fennell T, Ruan J, Homer N, Marth G, Abecasis G, Durbin R: The Sequence Alignment/Map format and SAMtools. *Bioinformatics (Oxford, England)* 25(16) 2009:2078–2079.
8. Li H, Durbin R: Fast and accurate short read alignment with Burrows-Wheeler transform. *Bioinformatics (Oxford, England)* 25(14) 2009:1754–1760.
9. Mandelbrot BB: The Fractal Geometry of Nature, *W. H. Freeman and Company*, New York, ISBN 0-7167-1186-9, 1983.
10. Peng CK, Buldyrev SV, Goldberger AL, Havlin S, Sciortino F, Simons M, Stanley HE: Long-range correlations in nucleotide sequences. *Nature* 356 1992:168-170.
11. Prabhu VV, Claverie JM Correlations in intronless DNA. *Nature* 359 1992:782.
12. Chatzidimitriou-Dreismann CA, Larhammar D: Long-range correlations in DNA. *Nature* 361 1993:212-213.
13. Borovik AS, Grosberg AY, Frank-Kamenetskii MD: Fractality of DNA Texts. *J Biomol Structure* 12(3) 1994:655-669.
14. Stanley HE, Buldyrev SV, Goldberger AL, Goldberger ZD, Havlin S, Mantegna RN, Ossadnik SM, Peng CK, Simons M: Statistical mechanics in biology: how ubiquitous are long-range correlations? *Physica A* 205 1994:214-253.
15. Li W: The study of correlation structures of DNA sequences: a critical review. *Comp Chem* 21(4) 1997:257-271.
16. Li W, Marr TG, Kaneko K: Understanding Long-range correlations in DNA sequences. *Physica D* 75 1994:392-416.
17. Kirkpatrick S, Stoll E: Implementation of the R250 random number generator. *J. Computational Physics* 40 1981: 517.
18. Maier WL: A fast pseudo random number generator. *Dr. Dobb’s Journal* 176, 1991.
