# Supplementary material for: The detailed 3D multi-loop aggregate/rosette chromatin architecture and functional dynamic organization of the human and mouse genomes
Source: Epigenetics Chromatin. 2016 Dec 24;9:58. doi: 10.1186/s13072-016-0089-x (PMC5192698; doi:10.1186/s13072-016-0089-x)
Supplement: Supplementary file 2 — Additional file 2: Table S1. Comparison between different chromosome interaction capture methods, showing their different application potential with respect to scientific aims and their signal-to-noise ratio which could function as an intrinsic quality statement (O: one; M: many; A: all; O<->O: one-to-one; P: primer; PCR: polymerase chain reaction; RE: restriction enzyme; Sel.PCR: selection with PCR; Seq: sequencing). [file 13072_2016_89_MOESM2_ESM.docx]

*Table S1:*

Comparison between different chromosome interaction capture methods, showing their different application potential in respect to scientific aims and their signal-to-noise ratio which could function as an intrinsic quality statement (O: one; M: many; A: all; O<->O: one-to-one; P: primer; PCR: polymerase chain reaction; RE: restriction enzyme; Sel.PCR: selection with PCR; Seq: sequencing).

| **Method** | **Interact-ion**  **Types** | **Interact-ion**  **Selection** | **1^st^**  **RE** | **Re-Lig.** | **2^nd^**  **RE** | **Ampli-fication** | **Noise Reduction** | **Readout** | **Signal-Noise**  **Ratio** |
| --- | --- | --- | --- | --- | --- | --- | --- | --- | --- |
| **3C** | O<->O | P/PCR | Y | Y | N | while  sel. PCR | none | gel | + |
| **3C-PCR** | O<->O | P/PCR | Y | Y | N | while  sel. PCR | none | qPCR | + |
| **4C** | O/M->A | P/PCR | Y | Y | Y/N | while  sel. PCR | none | fluoresc.  chip | + |
| **3C/4C-seq** | O/M->A |  | Y | Y | Y | Wwile  sel.PCR  and  for seq. | none | sequencing | ++ |
| **5C** | M<->M | P com/PCR | Y | Y | Y/N | while  sel. PCR | none | sequencing | ++ |
| **Hi-C** | A<->A | none | Y | Y | Y | for  seq. | re-ligation  bead selection | sequencing | ++ |
| **T2C** | arbitrary  combination | oligo  capture | Y | Y | Y | for  seq | oligo  placement | sequencing | +++ |
