# Supplementary material for: The detailed 3D multi-loop aggregate/rosette chromatin architecture and functional dynamic organization of the human and mouse genomes
Source: Epigenetics Chromatin. 2016 Dec 24;9:58. doi: 10.1186/s13072-016-0089-x (PMC5192698; doi:10.1186/s13072-016-0089-x)
Supplement: Supplementary file 3 — Additional file 3: Table S2. The quality and multiplexability of T2C is shown by a detailed overview of the regions investigated (grouped) on one capture array, of the Homo sapiens (HS) and Mus musculus (MM) genomes, with their chromosome and chromosomal position and size, the use of which 1st and 2nd restriction enzyme or in case of very high-resolution sonication, as well as the average fragment size calculated from $\left\langle {L_{Fragment} } \right\rangle = L_{Region} /N_{Fragment}$LFragment=LRegion/NFragment, and the number of oligos per region. The “name” of the region gives the borders with respect to the ideogram bands. [file 13072_2016_89_MOESM3_ESM.docx]

*Table S2:*

The quality and multiplexability of *T2C* is shown by a detailed overview of the regions investigated (grouped) on one capture array, of the homo sapiens (HS) and mus musculus (MM) genomes, with their chromosome and chromosomal position and size, the use of which first and second restriction enzyme or in case of very high resolution sonication, as well as the average fragment size calculated from $<L_{Fragment}> = L_{Region}/N_{Fragment}$ , and the number of oligos per region. The “name” of the region gives the borders with respect to the ideogram bands.

| **Capture**  **Array**  **Grouping** | **Region**  **“Name”** | **Chromosome**  **[N]** | **Chromosomal**  **Position** | | **Region Size**  **L_Region_**  **[bp]** | **Restriction Enzymes**  **or**  **Sonication** | | **Number**  **of**  **Frag-ments**  **N_Fragment_**  **[N]** | **Average**  **Frag-ment**  **Size**  **L_Fragment_**  **[bp]** | **Number**  **of**  **Capt-uring**  **Oligos**  **N_Oligos_**  **[N]** |
| --- | --- | --- | --- | --- | --- | --- | --- | --- | --- | --- |
|  |  |  | **Start**  **[bp]** | **End**  **[bp]** |  | **1st RE** | **2 nd RE/**  **Sonication** |  |  |  |
| single  capture array | 11p 15.4-15.5 | HS 11 | 1,110,650 | 3,216,350 | 2,105,700 | BglII | NlaIII | 344 | 6121 02612 | 525 |
| single  capture array | 7q E3-F1 | MM 7 | 109,876,350 | 111,966,600 | 2,090,250 | HindIII | NlaIII | 719 | 2915 | 800 |
|  | | | | | | | | | | |
| single  capture array | 3q A3-B | MM 3 | 32,548,927 | 36,548,927 | 4,000,000 | ApoI | sonication | 7,079 | 565 | 10,827 |
|  | 3q F1-F2.2 | MM 3 | 92,363,599 | 97,863,599 | 5,500,000 |  |  | 9,045 | 608 | 12,353 |
|  | 4q B1-B3 | MM 4 | 50,792,682 | 57,292,682 | 6,500,000 |  |  | 12,296 | 528 | 18,776 |
|  | 6q C1 | MM 6 | 64,999,977 | 70,999,977 | 6,000,000 |  |  | 13,466 | 445 | 13,737 |
|  | 6q F1-F3 | MM 6 | 119,932,440 | 125,432,440 | 5,500,000 |  |  | 9,292 | 591 | 11,780 |
|  | 7q C | MM 7 | 62,627,388 | 69,127,388 | 6,500,000 |  |  | 14,438 | 450 | 18,746 |
|  | 7q E3-F1 | MM 7 | 106,961,038 | 117,461,038 | 10,500,000 |  |  | 19,405 | 541 | 26,024 |
|  | 10q A2-A4 | MM 10 | 17,344,736 | 23,844,736 | 6,500,000 |  |  | 11,613 | 559 | 17,360 |
|  | 10q B4-B5.3 | MM 10 | 63,148,728 | 69,148,728 | 6,000,000 |  |  | 11,135 | 538 | 17,382 |
|  | 10q D3 | MM 10 | 125,635,645 | 130,135,645 | 4,500,000 |  |  | 7,437 | 605 | 10,773 |
|  | 11q A3.3-A5 | MM 11 | 29,683,511 | 37,183,511 | 7,500,000 |  |  | 11,726 | 639 | 18,387 |
|  | 12q F1-F2 | MM 12 | 110,657,161 | 118,657,161 | 8,000,000 |  |  | 12,108 | 660 | 16,156 |
|  | 14q B-C2 | MM 14 | 43,002,529 | 52,502,529 | 9,500,000 |  |  | 17,216 | 551 | 19,788 |
|  | 16q A2-B1 | MM 16 | 16,061,845 | 22,061,845 | 5,000,000 |  |  | 9,377 | 533 | 13,444 |
|  | 17q B1-B2 | MM 17 | 33,143,007 | 41,143,007 | 8,000,000 |  |  | 15,621 | 512 | 19,308 |
|  |  |  |  |  | ========  total  99,500,00 |  |  | =======  total  181,254 | ====  average 549 | ======  total  244,841 |
