# Supplementary material for: The detailed 3D multi-loop aggregate/rosette chromatin architecture and functional dynamic organization of the human and mouse genomes
Source: Epigenetics Chromatin. 2016 Dec 24;9:58. doi: 10.1186/s13072-016-0089-x (PMC5192698; doi:10.1186/s13072-016-0089-x)
Supplement: Supplementary file 4 — Additional file 4: Table S3. Sequencing and interaction statistics of the experiments done with T2C for the regions investigated (grouped) for the Homo sapiens (HS) and Mus musculus (MM) genomes, with respect to the number of capture arrays used, whether and how the multiplexing was done, results in sequenced reads of which a sub-fraction could be uniquely mapped, and finally sorted into square interaction matrices (notably, the matrix is mirrored at the diagonal), which can be analysed in total or according to whether the interactions are within the matrix or on the matrix diagonal concerning the number of existent interactions, their fraction of in total possible interactions, and the frequency distribution of the frequency of the interactions. For the high-resolved regions using Apo I as restriction enzyme and sonication as 2nd “restriction” due to the low number of sequence reads with respect to the total number of interactions no analysis concerning the interactions within the region was performed. [file 13072_2016_89_MOESM4_ESM.docx]

*Table S3:*

Sequencing and interaction statistics of the experiments done with *T2C* for the regions investigated (grouped) for the Homo sapiens (HS) and Mus musculus (MM) genomes, in respect to the number of capture arrays used, whether and how the multiplexing was done, results in sequenced reads of which sub-fraction could be uniquely mapped, and finally sorted into square interaction matrices (notably, the matrix is mirrored at the diagonal), which can be analysed in total or according to whether the interactions are within the matrix or on the matrix diagonal concerning the number of existent interactions, their fraction of in total possible interactions, and the frequency distribution of the frequency of the interactions. For the high-resolved regions using ApoI as restriction enzyme and sonication as second “restriction” due to the low number of sequence reads in respect to the total number of interactions no analysis concerning the interactions within the region was performed.

| Region | Cell  Type/  Experiment | Arrays  used  [1] | Multiplex  On  Array  [Y/N] | Seq.  Lane  [N] | Same  flow  cell  [Y/N] | Sequenced Reads  Total  Per  Region  x10^6^ [N] | Mappable Reads  Total  Per  Region  x10^6^ [N] | Number  Of Real Interactions  Per  Region  [N] | Interactions Within Region | | | | | | | | | | | |
| --- | --- | --- | --- | --- | --- | --- | --- | --- | --- | --- | --- | --- | --- | --- | --- | --- | --- | --- | --- | --- |
|  |  |  |  |  |  |  |  |  | Total Region | | | | Within Matrix (Without Diagonal) | | | | On Matrix Diagonal | | | |
|  |  |  |  |  |  |  |  |  | [N] | [%] | Frequency Distribution | | [N] | [%] | Frequency Distribution | | [N] | [%] | Frequency Distribution | |
|  |  |  |  |  |  |  |  |  |  |  | Avg  [N] | σ  [N] |  |  | Avg  [N] | σ  [N] |  |  | Avg  [N] | σ  [N] |
| HS 11p 15.5-15.4 | HB2 #1 | 1 | N | 1 | - | 51.95 | 13.70 | 58996 | 8873 | 14.95 | 1036 | 14433 | 8608 | 14.59 | 720 | 13802 | 256 | 74.4 | 11274 | 26120 |
|  | TEV #2 | 1 | Y  with  HRV | 2 | Y  with  HRV | 112.62 | 40.47 |  | 3355 | 5.65 | 2402 | 16265 | 3087 | 5.23 | 1167 | 12354 | 268 | 77.9 | 16626 | 36588 |
|  | HRV #2 | 1 | Y  with  TEV | 2 | Y  with  TEV | 306.25 | 17.05 |  | 4605 | 7.76 | 4386 | 33698 | 4322 | 7.32 | 1678 | 20999 | 283 | 82.2 | 45729 | 99777 |
|  | | | | | | | | | | | | | | | | | | | | |
| MM 7q E3-F1 | FB #2 | 1 | Y  with  FL #2 | 2 | Y  with  FL #2 | 84.98 | 7.29 | 258121 | 4193 | 1.62 | 1962 | 19189 | 3776 | 1.46 | 1366 | 14550 | 417 | 58.0 | 7355 | 41971 |
|  | FL #1 | 1 | N | 1 | - | 383.22 | 32.33 |  | 2886 | 1.11 | 821 | 6319 | 2506 | 0.97 | 458 | 3376 | 380 | 52.9 | 3212 | 14899 |
|  | FL #2 | 1 | Y  with  FB #1 | 2 | Y  with  FB #1 |  |  |  |  |  |  |  |  |  |  |  |  |  |  |  |
|  | | | | | | | | | | | | | | | | | | | | |
| MM 3q A3-B | FL | 1 | Parallel  Several  Regions | 1 | Y | 61.53 | 45.65 | 1181107456 | N.N. | | | | | | | | | | | |
| MM 3q F1-F2.2 |  |  |  |  |  |  |  |  |  |  |  |  |  |  |  |  |  |  |  |  |
| MM 4q B1-B3 |  |  |  |  |  |  |  |  |  |  |  |  |  |  |  |  |  |  |  |  |
| MM 6q C1 |  |  |  |  |  |  |  |  |  |  |  |  |  |  |  |  |  |  |  |  |
| MM 6q F1-F3 |  |  |  |  |  |  |  |  |  |  |  |  |  |  |  |  |  |  |  |  |
| MM 7q C |  |  |  |  |  |  |  |  |  |  |  |  |  |  |  |  |  |  |  |  |
| MM 7q E3-F1 |  |  |  |  |  |  |  |  |  |  |  |  |  |  |  |  |  |  |  |  |
| MM 10q A2-A4 |  |  |  |  |  |  |  |  |  |  |  |  |  |  |  |  |  |  |  |  |
| MM 10q B4-B5.3 |  |  |  |  |  |  |  |  |  |  |  |  |  |  |  |  |  |  |  |  |
| MM 10q D3 |  |  |  |  |  |  |  |  |  |  |  |  |  |  |  |  |  |  |  |  |
| MM 11q A3.3-A5 |  |  |  |  |  |  |  |  |  |  |  |  |  |  |  |  |  |  |  |  |
| MM 12q F1-F2 |  |  |  |  |  |  |  |  |  |  |  |  |  |  |  |  |  |  |  |  |
| MM 14q B-C2 |  |  |  |  |  |  |  |  |  |  |  |  |  |  |  |  |  |  |  |  |
| MM 16q A2-B1 |  |  |  |  |  |  |  |  |  |  |  |  |  |  |  |  |  |  |  |  |
| MM 17q B1-B2 |  |  |  |  |  |  |  |  |  |  |  |  |  |  |  |  |  |  |  |  |
