# Supplementary material for: The detailed 3D multi-loop aggregate/rosette chromatin architecture and functional dynamic organization of the human and mouse genomes
Source: Epigenetics Chromatin. 2016 Dec 24;9:58. doi: 10.1186/s13072-016-0089-x (PMC5192698; doi:10.1186/s13072-016-0089-x)
Supplement: Supplementary file 16 — Additional file 16: Table S4. General consensus loop sizes and thus position relative to the start of the first loop at the first loop base determined for human HB2, as well as HEK293T TEV (intact cohesin) and HRV (cleaved cohesin) cells of the IGF/H19 region at HS 11p 15.5–15.4. The subchromosomal domain size is calculated for domains with defined borders only from the sum of the loop sizes present. [file 13072_2016_89_MOESM16_ESM.docx]

*Table S4:*

General consensus loop sizes and thus position relative to the start of the first loop at the first loop base determined for human HB2, as well as HEK293T TEV (intact cohesin) and HRV (cleaved cohesin) cells of the IGF/H19 region at HS 11p 15.5-15.4. The subchromosomal domain size is calculated for domains with defined borders only from the sum of the loop sizes present.

| **Loop**  **[#]** | **Loop Size**  **[kbp]** | **Domain/Linker**  **[#]** |
| --- | --- | --- |
| 1 | 15.0 | Domain 1 |
| 2 | 45.9 |  |
| 3 | 56.0 |  |
| 4 | 43.0 |  |
| 5 | 56.7 |  |
| 6 | 48.1 |  |
| 7 | 63.2 | Linker 2 |
| 8 | 45.9 | Domain 2  728.5 |
| 9 | 59.6 |  |
| 10 | 51.7 |  |
| 11 | 37.3 |  |
| 12 | 76.1 |  |
| 13 | 28.7 |  |
| 14 | 46.7 |  |
| 15 | 25.1 |  |
| 16 | 76.8 |  |
| 17 | 25.8 |  |
| 18 | 59.6 |  |
| 19 | 13.6 |  |
| 20 | 42.3 |  |
| 21 | 47.4 |  |
| 22 | 51.7 |  |
| 23 | 40.2 |  |
| 24 | 33.7 | Linker 2 |
| 25 | 48.1 | Domain 3  403.4 |
| 26 | 39.5 |  |
| 27 | 44.5 |  |
| 28 | 63.2 |  |
| 29 | 46.7 |  |
| 30 | 53.8 |  |
| 31 | 48.8 |  |
| 32 | 25.1 |  |
| 33 | 33.7 |  |
| 34 | 43.1 | Linker 3 |
| 35 | 46.7 | Domain 4 |
| 36 | 73.2 |  |
| 37 | 43.8 |  |
| 38 | 63.2 |  |
| 39 | 62.4 |  |
| Average  StdDev  StdErr | 48.6±14.5±2.4  46.7±15.1±8.7 | Loops  Linker |
