# Supplementary material for: The detailed 3D multi-loop aggregate/rosette chromatin architecture and functional dynamic organization of the human and mouse genomes
Source: Epigenetics Chromatin. 2016 Dec 24;9:58. doi: 10.1186/s13072-016-0089-x (PMC5192698; doi:10.1186/s13072-016-0089-x)
Supplement: Supplementary file 17 — Additional file 17: Table S5. General consensus loop sizes and thus position relative to the start of the first loop at the first loop base determined for mouse fetal brain (FB; inactive β-globin) and fetal liver (FL; active β-globin) cells of the β-globin locus at MM 7q E3-F1. The subchromosomal domain size is calculated for domains with defined borders only from the sum of the loop sizes present. [file 13072_2016_89_MOESM17_ESM.docx]

*Table S5:*

General consensus loop sizes and thus position relative to the start of the first loop at the first loop base determined for mouse fetal brain (FB; inactive β-globin) and fetal liver (FL; active β-globin) cells of the β-globin locus at  q E3-F1. The subchromosomal domain size is calculated for domains with defined borders only from the sum of the loop sizes present.

| ***Loop***  ***[#]*** | ***Loop Size***  ***[kbp]*** | ***Domain/Linker***  ***[#]*** |
| --- | --- | --- |
| 1 | 40.6 | Domain 1 |
| 2 | 37.7 |  |
| 3 | 55.6 | Linker 1 |
| 4 | 52.7 | Domain 2  1343.6 |
| 5 | 39.9 |  |
| 6 | 84.1 |  |
| 7 | 50.6 |  |
| 8 | 78.4 |  |
| 9 | 47.3 |  |
| 10 | 88.3 |  |
| 11 | 51.3 |  |
| 12 | 53.4 |  |
| 13 | 59.8 |  |
| 14 | 47.4 |  |
| 15 | 47.7 |  |
| 16 | 57.7 |  |
| 17 | 86.9 |  |
| 18 | 53.4 |  |
| 19 | 72.0 |  |
| 20 | 44.9 |  |
| 21 | 54.2 |  |
| 22 | 93.3 |  |
| 23 | 77.7 |  |
| 24 | 52.7 |  |
| 25 | 49.9 |  |
| 26 | 82.7 | Linker 2 |
| 27 | 48.5 | Domain 3 |
| 28 | 37.8 |  |
| 29 | 45.0 |  |
| 30 | 83.4 |  |
| 31 | 42.7 |  |
| 32 | 62.0 |  |
| 33 | 48.5 |  |
| 34 | 60.6 |  |
| Average  StdDev  StdErr | 57.8±16.2±2.9  69.2±19.2±13.6 | Loops  Linker |
