# Supplementary material for: The detailed 3D multi-loop aggregate/rosette chromatin architecture and functional dynamic organization of the human and mouse genomes
Source: Epigenetics Chromatin. 2016 Dec 24;9:58. doi: 10.1186/s13072-016-0089-x (PMC5192698; doi:10.1186/s13072-016-0089-x)
Supplement: Supplementary file 18 — Additional file 18: Table S6. General consensus loop sizes and thus position relative to the start of the first loop at the first loop base determined for mouse MEL cells of one part of the Igh locus at MM 12q F1-F2. The subchromosomal domain size is calculated for domains with defined borders only from the sum of the loop sizes present. [file 13072_2016_89_MOESM18_ESM.docx]

*Table S6:*

General consensus loop sizes and thus position relative to the start of the first loop at the first loop base determined for mouse MEL cells of one part of the IGH locus at  12q F1-F2. The subchromosomal domain size is calculated for domains with defined borders only from the sum of the loop sizes present.

| ***Loop***  ***[#]*** | ***Loop Size***  ***[kbp]*** | ***Domain/Linker***  ***[#] [kbp]*** |
| --- | --- | --- |
| 1 | 33.8 | Domain 1  333.3 |
| 2 | 47.5 |  |
| 3 | 52.7 |  |
| 4 | 38.7 |  |
| 5 | 22.0 |  |
| 6 | 23.4 |  |
| 7 | 38.6 |  |
| 8 | 38.7 |  |
| 9 | 37.8 |  |
| Average  StdDev  StdErr | 37.0±9.9±3.3 | Loops |
