# Supplementary material for: The detailed 3D multi-loop aggregate/rosette chromatin architecture and functional dynamic organization of the human and mouse genomes
Source: Epigenetics Chromatin. 2016 Dec 24;9:58. doi: 10.1186/s13072-016-0089-x (PMC5192698; doi:10.1186/s13072-016-0089-x)
Supplement: Supplementary file 21 — Additional file 21: Supplemental Results. [file 13072_2016_89_MOESM21_ESM.docx]

**Supplemental Results:**

**Simulated polymer models *in silico* predict and confirm the genome organization in detail found by T2C**

To better understand the above results and to evaluate hypotheses and plan future experiments concerning the 3D genome organization in eukaryotes we developed polymer models with pre-set conditions (i.e. without attempting to fit data; [3,5,7,8,15,59,87,88]). The simulations (see Suppl. Methods) use a stretchable, bendable, and volume excluded polymer (hydrodynamic) approximation of the 30 nm chromatin fibre consisting of individual homogenous segments with a resolution of ~1-2.5 kbp while combining Monte Carlo and Brownian Dynamics approaches. The simulations cover the Random-Walk/Giant-Loop model (Figure S10) in which large individual loops (0.5–5.0 Mbp) are connected by a linker resembling a flexible backbone, as well as the Multi-Loop Subcompartment (MLS) model (Figure S10) with rosette-like aggregates (0.5–2 Mbp) with smaller loops (60–250 kbp) connected by linkers (60–250 kbp). These models also contain enough information/aspects to cover other architectures such as free random-walks, random or fractal globules. They also cover their stability and dynamics. Two-dimensional spatial distance and interaction maps (for different crosslink conditions) were calculated with high statistical validity (Figure 1E, F; Figure S8, S9). Even in the absence of simulated nucleosomes, comparison of the simulations to our experimental results shows, that only an MLS- and thus loop aggregate/rosette-like genome architecture could explain all the above observations. Thus, also these simulations confirm previous predictions (see introduction; [4,5,7-10,15,17,47,51-54,59,61,87,88]). Even slight variations of the simulated topologies (see Suppl. Methods; Table S7) are reflected in the simulation results: i) the interaction frequency in general depends on the spatial proximity and the (cell dependent) crosslink probability (here simulated by the crosslink kinetics in different volumes and thus interaction radii), ii) the appearance of the subchromosomal domains, subchromosomal domain separation, loops, and number of loops in a rosette (and thus density per subchromosomal domain) of the MLS model or loop size and separation in the RW/GL model are proportional to their size and number. The simulations also show more subtle effects for special combinations of parameters: i) high numbers of especially small loops in an MLS rosette result due the high density in steric exclusion and thus stretched loops eventually even “shielding” inner-rosette parts, or ii) inter-domain interactions are influenced by the connecting linker, loop size and numbers, and how non-equilibrium effects would appear (note: we deliberately show here not entirely equilibrated simulations; see also [5,59,87,88]). This also sheds light on the behaviour of domain borders near the linker (see above). The simulations support also the large emptiness of interaction matrices and its link to the existence of a dedicated chromatin quasi-fibre. Additionally, the simulations hint to a relatively low crosslink probability, radius, and frequency in experiments comparing the clearly visible fine-structure (such as the (anti-)parallel neighbouring of the chromatin quasi-fibre at loop bases; Figure 1D-F) and its dependence on parameter changes in simulations.

The stability of the architecture with respect to the intrinsic chromatin fibre dynamics can also be illustrated by e.g. the decondensation from a mitotic chromosome into interphase (Movie S1). This also shows that any 3D architecture would dissolve within seconds if it would not be stable. This agrees with the analytical polymer models developed recently to describe both structure and dynamics of the chromatin quasi-fibre [11]. The subtle excluded volume effects or the fine structure of in/out-going loops at the loop aggregate/rosette core are not yet described to such detail in the analytical polymer model.

The *in silico* simulation approach was also used to visualize the 3D organization and its dynamics using the experimental interaction matrices as input. *In vivo* chromosomes never fold from scratch, they always have a history, and transform from one state to the other continuously. Thus, genomes are adiabatic systems, and hence we used here the consensus loop and domain positions (Table S4-6) as input starting conditions, rather than dropping a free linear polymer chain into the interaction landscape expecting it to fold in a defined knot-free 3D architecture. Only after a first Brownian Dynamics relaxation, the entire interaction matrix is used as additional side condition to introduce individual folding characteristics such as e.g. specific loop-loop interactions, which are not *a priori* contained implicitly in the consensus architecture. The outcome (Figure 1B-D, middle) confirms that the chromatin quasi-fibre forms rosette-like subchromosomal domains with a high degree of agreement with the experiments and the analytic model mentioned above [11].

**Simulations show a fine-structured multi-scaling scaling behaviour revealing general aspects of genome organization**

To comprehensively investigate and quantify the general behaviour of interactions as a function of genomic separation in a unified scale-bridging manner from a few to the mega base pair level, we already used scaling analysis to understand genome organization and showed its capabilities (see Suppl. Methods; Figure S11; [5,16,59]). Scaling analyses condense e.g. the two-dimensional interaction or spatial distance matrices into a one-dimensional scaling plot with higher statistical relevance to find signatures/patterns in a spectroscopy-like manner. For ease of understanding, we first introduce the scaling of the interaction frequency for the different simulated models (see Suppl. Methods; Figure 2B; Figure S12, S13): All MLS and RWGL simulations show clear long-range power-law scaling, with a multi-scaling behaviour and a fine-structure on top which are attributable to i) the general interaction decrease upon increasing spatial distance, ii) the subchromosomal domain or giant loop like structure, iii) the loop structure within the subchromosomal domains and/or the random-walk behaviour within the loops, and iv) the random-walk behaviour of the linker between the subchromosomal domain (i.e. the “backbone” behaviour of the entire chromosome), or that of giant loops. In the MLS models (Figure 2B; Figure S12) the clustered loops form a plateau with an ever steeper slope, which at scales > ~10^6^ bp is dominated by the random-walk behaviour of the linker between the rosettes in agreement with polymer physics. The RWGL models (Figure 2B; Figure S13) are dominated by the random-walk behaviour within the mega-base pair loops and the linker between the loops. Obviously, the visible fine-structure is due to the loops, their size, and aggregation. The interaction itself and the pronounced fine-structure is inversely proportional to the interaction radius, i.e. to the spatial dynamics and kinetics of the crosslink. Again all model parameter variations are represented in the scaling behaviour on all scales in detail. The same holds for other scaling measures such as the spatial distance dimension or exact yard-stick dimensions directly measuring the scaling of the fibre folding (Figure S11). The simulations show that there is no uniform scaling across all scales as e.g. seen in self-similar fractals bridging large orders of magnitude and hence this deviation shows the sub-structuring into loops and subchromosomal domains. Again this is in excellent agreement with the alternative analytical model [11]. Consequently, scaling analysis is a relatively easy and adequate measure to analyse genome topologies in a scale bridging manner with a high degree of detail and statistical relevance.

**T2C shows fine-structured multi-scaling suggesting in detail loop aggregate/rosette 3D architectures**

With this background we determined the experimental scaling behaviour (see Suppl. Methods) of the IGF/H19 locus, the β-globin region (Figure 2A; Figure S14) and that of the average of 15 regions in MEL cells (Figure 2C,D; Figure S15). In contrast to the simulations, the higher resolution of T2C allows a study of nucleosome compaction (see below), and whereas for the ~2.1 Mbp regions the scaling cut-off is already reached at ~10^6^ bp, the T2C design for the 15 regions reaches the cut-off later with a smaller frequency range for medium and large scales. For scales >10^4^ bp, all interactions clearly show fine-structured multi-scaling long-range power-law behaviour (Figure 2A; Figure S14), the details of which are only in agreement with the multi-loop aggregate/rosette like architecture (Figure 2B; Figure S12, S13) as predicted by us [5,15,16,59]. The behaviour represents i) the general interaction decrease, i.e. spatial distance increase, of the chromatin quasi-fibre up to ~3x10^4^ to 10^5^ bp, ii) the stable loop and aggregated-loop/rosette-like structure in the subchromosomal domains from ~3x10^4^ up to 10^5^-10^6^ bp, iii) the subchromosomal-domain-like structure from ~10^5^-10^6^ bp, and iv) the random-walk behaviour of the domain linkers above ~0.8x10^6^ bp (until the cut-offs). As before the differences between species, cell type, or functional states are again small and the behaviour again shows the stability and functional variability of the system. We also found this scaling behaviour for Hi-C experiments of others (e.g. [71,73,74,76]), suggesting the same 3D architecture (Imam et al., in prep.).

**High-resolution T2C scaling analysis reveals the detailed nucleosome structure and proves the formation of a chromatin quasi-fibre**

Interestingly, we also get a dedicated fine-structured multi-scaling behaviour on scales from the base pair level up to 10^4^ bp [5,15,16]. This is especially true for the average of the scaling curves of the 15 regions totalling ~99 Mbp regarding the high resolution of a few base pairs and the high statistical validity (Figure 2C,D; Figure S14, S15). Note that at such molecular scales i) the cleavage probability by the first T2C restriction enzyme depends critically on the local structure(s), ii) the crosslinked and (un)cleaved DNA would still be linked to the nucleosomal core and/or other factors, and iii) that this and the DNA fragment size (below or near the free DNA optimal end-to-end interaction probability), all influence the religation probability as well. All these factors alone or in combination may lead to the exploration of the entire possible spectrum of results. Nevertheless, distinguishing between all and “secured” interactions, i.e. including only sequentially non-adjacent neighbours and leaving out interactions based on uncleaved, i.e. non-religation events, confirms the validity of the results (Figure 2C,D; Figure S14, S15).

From a few base pairs onwards, the interaction frequency increases towards a plateau between ~50 bp and ~100 bp, followed by a sharp peak around 145 bp which is ~1.5 orders of magnitude higher and ranges from ~110 to 195 bp (width ~85 bp). This is followed by a slightly decreasing plateau from ~230 bp up to the transit to a new descent at ~10^3^ bp which then obviously changes to a decay and at ~10^4^ bp to the known multi-scaling behaviour for bigger scales (Figure 2D). In the case of the lower-resolved IGF/H19 locus and the β-globin region, the plateau between ~230 and ~10^3^ bp is more a plateaued valley (Figure 2A; Figure S14) and again independent of species, cell type, or functional state. Taking “secured” interactions leaves out the behaviour below ~195 bp but thereafter results in an increase to the plateau or plateaued valley at ~230 bp up to the transit to the known descent at ~10^3^ bp, with a hardly different behaviour thereafter (Figure 2C; Figure S14).

On top there is a dedicated fine-structure (Figure S15), which up to ~195 bp can be associated with the nucleosome and with the polymer behaviour of the nucleosomal chain thereafter: The most prominent feature is a small peak on the major peak at 145.5 bp (Figure S15C), representing the winding of the DNA around the nucleosome, but other peaks are also where one would expect structural nucleosomal features (see below and [14]) and as we found them already by DNA sequence pattern analysis (see below; [5,15,16]). Actually, the T2C peaks from scales of 10 to 195 bp (the values < 10 bp in Figure 1A, C, D; Figure S14 are due to the algorithm used and for transparency not discarded since they nevertheless show the extrapolation from values > 10 bp) are equal to those found in nucleosomal binding sequences, the first plateau is associated with the nucleosomal linker sequence and the DNA only bound once to the nucleosome, the width of the main peak associates with the DNA sequence double wound around the nucleosome, and its upper border corresponds to the nucleosomal repeat length at 195 bp. On the second plateau from ~230 to ~10^3^ bp there might be a fine-structure as multiples of the 145.5 bp and the 195 bp nucleosomal repeat-length, e.g. at 290 bp as well as at 385 bp the peaks are exactly where di-nucleosomal features are expected (Figure 2C; Figure S15B). Restriction enzyme dependencies might influence this as well although they seem to play only a minor or no role at all.

The plateaued valley at lower resolution and the plateau at high-resolution with a slight decrease of ~10% for the interaction probabilities from nucleosome N1 to nucleosomes N4-N6, suggest that nucleosomes N4-N6 see the first nucleosome with nearly the exact same probability. Furthermore, for N7 the interaction decreases dramatically. Thus, each individual nucleosome has on average 4-6 clearly distinct nearest neighbour nucleosomes. This strongly suggests the formation of a chromatin quasi-fibre with an average density of 5±1 nucleosomes per 11nm, since the maximum distance between N1 and N2 can only be a stretched out DNA linker of ~50 bp equalling ~14 nm and for a crosslink this difference has to come down to a few Ås to form the covalent bond (neglecting the nucleosomal tails and the probability of crosslinking with a “stretched” DNA linker). Moreover, the genome wide *in vivo* FCS measurements of the dynamics of the chromatin quasi-fibre [11] show similar average quasi-fibre densities. We would like to stress once more that the interaction matrices show that this is only an average and thus for calling this a “quasi”-fibre.

**Apparent and average persistence length L_p_ of the chromatin quasi-fibre**

To gain insight into the average mechanical properties of the chromatin quasi-fibre we calculated the average apparent persistence length L_p_ from the interaction scaling behaviour between 10^3^ and 10^4^ bp. For the calculation we assume the analytical model of an average quasi-fibre performing a (self-avoiding) random-walk (see theory in e.g. [5,11,59,87]) and importantly equal crosslinking kinetics for chromatin, and fit this to this region. This results in L_p_ between ~1000 nm at ~10^3^ bp down to 5 nm at ~10^4^ bp. To gain a realistic value, one has additionally to take into account the notion that L_p_ is only defined above ~2-3 times the basic fibre unit of 5±1 nucleosomes and for a free unlooped fibre, and thus well below the chromatin loop size, which in our case with an 10x oversampling means < 4 kbp. From the DNA sequence correlations (see below) this “sweet” point is at ~3.6 kbp (Figure 2E). Thus, at 3.6 kbp for 4-6 nucleosomes per 11nm, L_p_ ranges from ~80 to 120 nm, respectively. This is in agreement with earlier values (see introduction; [32,33]) and with values derivable from spatial distance measurements between genetic markers [5,7,8,87]. This average stiffness predicts that the average loop sizes will have to be on the scale seen above to ensure e.g. their stability, although, we would like to stress that this is only an average L_p_, and that L_p_ also varies with the quasi-fibre. Consequently, the notion of an apparent persistence length is only applicable in the context of a local L_p_, with a local meaning while taking the limit of the “genomic” statistical mechanics and uncertainty into account. This too agrees with values for L_p_ extractable from recent genome wide *in vivo* FCS measurements [11].

**The DNA sequence organization shows fine-structured multi-scaling long-range correlations tightly entangled with the 3D architecture**

Since what is near in physical space should also be near (i.e. in terms of similarity) in DNA sequence space and this presumably genome wide [5,15,16], and because evolutionary surviving mutations of all sorts will be biased by the genome architecture itself and vice versa, we also investigated the correlation behaviour of the DNA sequence (see Suppl. Methods; [5,15,16,40] and references therein). To this end, we used the most likely simplest correlation analysis possible (to avoid information loss or being biased) and calculated the mean square deviation of the base pair composition (purines/pyrimidines) within windows of different sizes. In other words we calculated the function C(l) and its local slope δ(l), which is a measure for the correlation degree, or in more practical lay-men terms similar to a spectral measure (see Suppl. Methods; [5,15,16,40]) for two different human and mouse strains (Figure 1E; Figure S16-21): i) long-range power-law correlations were found on almost the entire observable scale, ii) with the local correlation coefficients showing a species specific multi-scaling behaviour with close to random correlations on the scale of a few base pairs, a first maximum from 40 to 3.6 kbp, and a second maximum from 8x10^4^ to 3x10^5^ bp, and iii) an additional fine-structure in the first and second maxima is present. The correlation degree and behaviour is stronger in human compared to mouse, but within the species nearly identical comparing different chromosomes (with larger differences for the X and Y chromosome). The behaviour on all scales is equivalent concerning the different measures used (Figure 2B; Figure S11-13) to the long-range multi-scaling of the genome architecture with the transitions of behaviours even at similar scaling positions. Consequently, we can associate the behaviour with i) the nucleosome, ii) the compaction into a quasi-fibre, iii) the chromatin fibre regime, iv) the formation of loops, v) subchromosomal domains, and vi) their connection by a linker. Especially the transition from the basic nucleosomal compaction into the quasi-fibre regime at ~3.6 kbp (what we called the sweet point in the calculation of the persistence length) is clearly visible. Additionally, on the fine-structural level, the already previously proven association to nucleosomal binding at the first general maximum [5,15,16,40] is not only found again (Figure S16), but also is in agreement with the fine-structure found in the interaction scaling (Figure S15). This in principle holds now also for the fine-structure visible in the second general maximum as well and now even in the entire chromosome set and in two versions of two species and is associated with the loop aggregated/rosette structure there, predicting loop sizes from ~30 to 100 kbp, and subchromosomal domain size from ~300 kbp to ~1.3 Mbp (see also [5,16]). We have, however, to acknowledge, that the experimental T2C data have only encompassed a small fraction of the genome yet, whereas the sequence correlations encompass the entire genome, which hints clearly that this is a genome wide phenomenon. Moreover, the existence and details of this behaviour show the stability and persistence of the architecture since sequence reshuffling or other destructive measures would result in a loss of this pattern. This would also be the case for an unstable architecture, which would not leave a defined footprint within the sequence. This is again in agreement with our simulations of the dynamics or the genome wide *in vivo* FCS measurements [11]. Consequently, we show not only by two analysis of completely independent “targets” (the T2C interaction experiments and the analysis of the DNA sequence) the compaction into a chromatin quasi-fibre and a loop aggregate/rosette like genome architecture again, but prove here also the long discussed notion, that what is near in physical space is also near, i.e. more similar, in sequence space. Since all chromosome sequences show a highly similar behaviour this clearly shows the genome wide validity. Hence the 3D architecture and DNA sequence organization are coevolutionary tightly entangled (review of previous notions in [5,16]). Thus, in the future from the DNA sequence and other higher-order codes (e.g. the epigenetic code) most architectural genome features can be determined, since also most structural/architectural features and vice versa left a footprint on the DNA sequence and other code levels as one would expect from a stable scale bridging systems genomic entity.
