# Supplementary material for: The detailed 3D multi-loop aggregate/rosette chromatin architecture and functional dynamic organization of the human and mouse genomes
Source: Epigenetics Chromatin. 2016 Dec 24;9:58. doi: 10.1186/s13072-016-0089-x (PMC5192698; doi:10.1186/s13072-016-0089-x)
Supplement: Supplementary file 22 — Additional file 22: Table S7. Simulated chromosome models with their physical properties (as in detail described Knoch [3, 4, 87, 88]): The band number is the number of subcompartments or loops per chromosome. The average theoretic loop size is $\left\langle R_{L}\right\rangle = \sqrt {\left( {300nm} \right)^{2} \cdot L_{S} /\left( {2 \cdot 300nm} \right)}$RL=300nm2·LS/2·300nm and was determined from simulated position-dependent (PD) and position-independent (PI) spatial distances for genomic separations at half the loop size. The average simulated band size is the average extension of the mass distribution. The average theoretic band distance is $\left\langle R_{B}\right\rangle = \sqrt {\left( {300nm} \right)^{2} \cdot LI_{L} /\left( {300nm} \right)}$RB=300nm2·LIL/300nm and was simulated from the average spatial distance averages between succeeding subcompartments. The average theoretic territory size is $\left\langle R_{Ltotal}\right\rangle = \sqrt {\left( {300nm} \right)^{2} \cdot \left( {NB - 1} \right) \cdot LI_{L} /\left( {300nm} \right)}$RLtotal=300nm2·NB-1·LIL/300nm and was simulated from the average mass distribution extension. Naming of models: [model name]-[loop length]-[linker length]. [file 13072_2016_89_MOESM22_ESM.docx]

*Table S7:*

Simulated chromosome models with their physical properties (as in detail described Knoch, 1998; Knoch 2002; Knoch, submitted; Knoch submitted): The band number is the number of subcompartments or loops per chromosome. The average theoretic loop size is $\left\langle R_{L} \right\rangle=\sqrt{\left( 300nm \right)^{2}\cdot L_{S}/(2\cdot300nm)}$ and was determined from simulated position dependent (PD) and independent (PI) spatial distances for genomic separations at half the loop size. The average simulated band size is the average extension of the mass distribution. The average theoretic band distance is $\left\langle R_{B} \right\rangle=\sqrt{\left( 300nm \right)^{2}\cdot{LI}_{L}/(300nm)}$ and was simulated from the average spatial distance averages between succeeding subcompartments. The average theoretic territory size is $\left\langle R_{Ltotal} \right\rangle=\sqrt{\left( 300nm \right)^{2}\cdot\left( NB-1 \right)\cdot{LI}_{L}/(300nm)}$ and was simulated from the average mass distribution extension. Naming of models: [model name]-[loop length]-[linker length].

| **Model** | **Loop properties** | | | | | **Linker properties** | | **Band properties** | | | **Mean band distance** | | **Mean territory size** | |
| --- | --- | --- | --- | --- | --- | --- | --- | --- | --- | --- | --- | --- | --- | --- |
|  | Size  L_S_  [Mbp] | Length  L_L_  [µm] | Theoretic  Spatial  Size  [nm] | Simulated  PI-Spatial  Size  [nm] | Simulated  PD-Spatial  Size  [nm] | Size  LI_S_  [Mbp] | Length  LI_L_  [µm] | # of  bands  per  chrosome  NB  [N] | # of  loops  per  rosette  [N] | Simulated  band size  [nm] | Theoretic  <RB>  [nm] | Simulated  <RB>  [nm] | Theoretic  <RLtotal>  [µm] | Simulated  <RLtotal>  [µm] |
| MLS-84-126 | 0.084 | 0.8 | 490 | 230 | 195 | 0.126 | 1.2 | 96 | 11.8±6.8 | 420 | 600 | 615 | 5.85 | 5.9 |
| MLS-105-126 | 0.105 | 1.0 | 548 | 270 | 240 | 0.126 | 1.2 | 96 | 9.2±5.2 | 500 | 600 | 622 | 5.85 | 6.0 |
| MLS-126-63 | 0.126 | 1.2 | 600 | 320 | 275 | 0.063 | 0.6 | 96 | 7.9±4.3 | 700 | 424 | 520 | 3.13 | 4.0 |
| MLS-126-126 | 0.126 | 1.2 | 600 | 320 | 275 | 0.126 | 1.2 | 96 | 7.5±4.3 | 700 | 600 | 630 | 5.85 | 6.1 |
| MLS-126-189 | 0.126 | 1.2 | 600 | 320 | 275 | 0.189 | 1.8 | 96 | 7.1±4.3 | 700 | 734 | 720 | 7.16 | 7.3 |
| MLS-126-252 | 0.126 | 1.2 | 600 | 320 | 275 | 0.252 | 2.4 | 96 | 6.5±4.3 | 700 | 848 | 870 | 8.27 | 8.4 |
| MLS-158-126 | 0.158 | 1.5 | 671 | 350 | 300 | 0.126 | 1.2 | 96 | 6.1±3.6 | 600 | 600 | 625 | 5.85 | 5.9 |
| MLS-252-126 | 0.252 | 2.4 | 848 | 425 | 375 | 0.126 | 1.2 | 96 | 3.8±2.0 | 860 | 600 | 610 | 5.85 | 5.8 |
|  | | | | | | | | | | | | | | |
| RW/GL-126-63 | 0.126 | 1.2 | 600 | 330 | 285 | 0.063 | 0.6 | 561 | - | - | 424 | 450 | 10.0 | 9.7 |
| RW/GL-252-63 | 0.252 | 2.4 | 848 | 430 | 405 | 0.063 | 0.6 | 338 | - | - | 424 | 440 | 7.79 | 7.3 |
| RW/GL-504-63 | 0.504 | 4.8 | 1200 | 610 | 595 | 0.063 | 0.6 | 187 | - | - | 424 | 439 | 5.79 | 5.6 |
| RW/GL-1000-126 | 1.0 | 10 | 1732 | 885 | 870 | 0.126 | 1.2 | 94 | - | - | 600 | 620 | 5.82 | 6.3 |
| RW/GL-2000-189 | 2.0 | 20 | 2449 | 1320 | 1300 | 0.189 | 1.8 | 48 | - | - | 734 | 740 | 5.09 | 5.8 |
| RW/GL-3000-252 | 3.0 | 30 | 3000 | 1530 | 1515 | 0.252 | 2.4 | 33 | - | - | 848 | 840 | 4.80 | 5.6 |
| RW/GL-4000-315 | 4.0 | 40 | 3264 | 1890 | 1880 | 0.315 | 3.0 | 25 | - | - | 948 | 960 | 4.64 | 6.4 |
| RW/GL-5000-378 | 5.0 | 50 | 3873 | 2240 | 2235 | 0.378 | 3.6 | 20 | - | - | 1000 | 1010 | 4.53 | 7.0 |
